# Supplementary material for: Multi-domain automated patterning of DNA-functionalized hydrogels
Source: PLoS One. 2024 Feb 2;19(2):e0295923. doi: 10.1371/journal.pone.0295923 (PMC10836684; doi:10.1371/journal.pone.0295923)
Supplement: S1 File — (PDF) [file pone.0295923.s001.pdf]

Supporting Information

Multi-domain automated patterning of DNA-functionalized hydrogels

Moshe Rubanov[1], Joshua Cole[1¶], Heon-Joon Lee[2¶], Leandro G. Soto Cordova[1], Zachary Chen[1], Elia Gonzalez[1] and Rebecca Schulman[1,3,4]*

[1] Department of Chemical and Biomolecular Engineering, Whiting School of Engineering, Johns Hopkins University, Baltimore, Maryland, United States of America

[2] Department of Biomedical Engineering, Whiting School of Engineering and the School of Medicine, Johns Hopkins University, Baltimore, Maryland, United States of America

[3] Department of Computer Science, Whiting School of Engineering, Johns Hopkins University, Baltimore, Maryland, United States of America

[4] Department of Chemistry, Krieger School of Arts and Sciences, Johns Hopkins University, Baltimore, Maryland, United States of America

* Corresponding author

E-mail: rschulm3@jhu.edu (RS)

[¶]These authors contributed equally to this work.

Supplemental Information Table of Contents

1. Pycromanager 4
2. Pseudocode and Python code 4
3. Ink vial design 6
4. Protocol for the fabrication of a silicon master mold for the 100 µm tall microfluidic flow chamber . 6
5. Protocol for fabricating PDMS: Glass microfluidic flow chambers 7
6. Single-domain hydrogel fabrication protocol 8
7. Data and analysis for single-domain hydrogel photopatterning in 100 µm tall flow chamber 11
8. Protocol for the fabrication of a silicon master mold of the 20 µm tall microfluidic flow chamber .. 12
9. Protocol, data, and analysis for MAPDH in 20 µm tall flow chamber 12
10. Multi-domain hydrogel fabrication protocol 15
11. Micrograph processing for Figure 2J 18
12. Location mapping from pixel art 19
13. Integration of location maps for use with MAPDH 20
14. Pixel art multi-domain fabrication protocol 21
15. Micrograph Processing for Figure 3 23
16. MAPDH with addressable hybridization protocol 24
17. Figure 4 micrograph processing 27
18. Protocol for fabricating the silicon mold for the sacrificial layer boat 29
19. Protocol for fabricating the sacrificial layer within the PDMS boat 30
20. Protocol for fabricating lift-off flow chambers 31
21. Protocol for single-domain hydrogel fabrication and liftoff 31
22. Analysis of protocol for fabrication of single-domain hydrogels using MAPDH-LC 34
23. Protocol for multi-domain hydrogel fabrication, liftoff, and collection 35
24. Analysis for multi-domain MAPDH-LC 38
25. Protocol for single-domain, DNA-crosslinked hydrogel fabrication, lift-off, and collection 39
26. Protocol for swelling single-domain hydrogels 42
27. Automated measurement of hydrogel lengths 42
28. Analysis for single-domain hydrogel swelling 43
29. Protocol for multi-domain, DNA-crosslinked hydrogel fabrication, lift-off, and collection 44
30. Protocol for swelling of multi-domain hydrogels 47

31. Analysis for multi-domain hydrogel swelling 48
32. MAPDH Equipment 49
33. MAPDH Reagents and components 50
34. Sequences 51
35. Bibliography 51

1. Pycromanager

MAPDH uses open-source software to integrate and control all components. Specifically, MAPDH uses Micromanager [1] to integrate control to the following components: the camera, microscope filter turrets (for patterning or imaging of hydrogels), XY stage, digital micromirror microfluidic flow chamber (DMD), UV LED (for patterning), white LED (for imaging) and an Arduino used for control of electronic solenoids. We additionally use Pycro-Manager to control Micro-Manager using Python scripts [2].

To facilitate quick scripting, we created a package with a set of functions for controlling all of these components. The functions can be broken into 4 different utilities:

1. Micromanager interface
   - This includes initialization: establishing a Python connection to Micromanager and setting microfluidic flow chamber states for the microscope and the digital micromirror microfluidic flow chamber (DMD)) and position lists that can be extracted for patterning.
2. Flow controller functions
   - This includes functions for turning on and off solenoid valves (and thus flowing in new inks / washes) for specified amounts of time.
3. Mask generator functions
   - This includes functions to make digital masks such as those shown in **Figures 2-5**, as well as to resize and upload them to the DMD.
4. Patterning functions
   - This includes functions for exposing a mask for a given set of time *(i.e.*, exposure), as well as more complex patterning functions, such as patterning grid of hydrogels within the same domain.

Not all components were required for each experiment or protocol. For example, for protocols that were done semi-automatically, the flow controller component was not used. In this case, flowing different inks and washes was performed by attaching syringes to the inlets/outlet of the patterning chamber and subsequently flowing in ink by applying pressure to the syringes. The patterning steps – specifying sizes, locations, and shapes of the hydrogels – was done in Python using utilities 1, 3, and 4.

2. Pseudocode and Python code

To simplify the scripting language for MAPDH, we developed a simple syntax for representing MAPDH tasks. The pseudocode from **Figure 1B,** shown below, is an example of a set of instructions in a loop that the MAPDH hardware carries out during the fabrication process represented in this markup.

```
vials = [1, 2, 3, 4]
for i in inks: #Patterning Rounds
Flow(i)
for j in range(5): #5 gels per round
Move(hydrogels[i,j].location)
Flow(i)
Pattern(hydrogels[i,j].mask)
Wash()
```

This markup could also be written as pseudocode, *i.e.*:

1. Flow in ink 1

2. Move to location for hydrogel [i,j]
3. Flow in ink i
4. Pattern hydrogel [i,j] using mask [i,j]
5. Wash with buffer
6. Repeat 1-5 for inks 2, 3 and 4.

Hydrogel[i,j] is a two-dimensional array where each element within the array contains a digital mask and location to photopolymerize one hydrogel. The rows (i) represent the different inks, whereas the columns (j) represent all hydrogels patterned using ink i. For example, hydrogel[2,3] is the 3rd hydrogel patterned with the 2nd ink. The actual Python code that MAPDH used to fabricate the hydrogels in **Figure 1B** is shown below:

```
from pycromanager import Bridge
import time
import patterning_functions_v4 as PF

h = 684
w = 608
radius = 100
bridge = Bridge(convert_camel_case=False)
core = bridge.get_core()
DMD = core.getSLMMicrofluidic flow chamber()
h = core.getSLMHeight(DMD)
w = core.getSLMWidth(DMD)

PF.init() #Initialization for all hardware
pos_list = PF.position_list() #Position list extraction from micromanager

# 1 DMD pixel = 0.45 um
# Square and Objective Parameters
square_side = 50
CF = 0.45
square_conv = square_side / CF
draw_square = PF.square_mask_generator(h,w,ex=square_conv)
square_scaled = PF.mask_rescaler(h, w, draw_square) #scale square mask to pattern 50 um gels

vials = [1,2,3,4] #Vials used
wait = 90 #Wait in seconds
dist = 100 #Distance between each hydrogel
exp = 0.5 #Exposure in seconds

for i in vials:
core.setXYPosition(pos_list[i,0],pos_list[i,1])
time.sleep(3)
PF.valve_timer(i,wait)
for j in range(5):
core.setRelativeXYPosition(-dist,0)
time.sleep(3)
PF.Patterning(exp,square_scaled)
time.sleep(3)
```

To connect an existing Python script directly to the MAPDH hardware, we first installed and connected Micromanager to interface with the hardware (**SI Section 32**). Then, Pycromanager [2] was installed to

connect to Micromanager through a Python script. The code uses a custom *patterning_functions* package to initialize the hardware, draw and upload masks, to inject inks, and to expose and pattern the hydrogels. All code and hardware configuration files can be found at https://github.com/MishaRubanov/MAPDH. We term the code that integrates all the hardware towards fabricating hydrogel architectures the MAPDH script.

3. Ink vial design

The ink vials are air-tight, low dead-volume containers that use air pressure to control the flow of ink from the bottom of a conical vial (**SI Figure 1**). The vials have two holes drilled using a microdrill into their lids: one hole is for a hollow point needle that is connected to an air source that provides input pressure from the flow controller and one hole with a small diameter (0.01") for the ink/wash tubing fed through to the bottom of the vial that feeds the ink/wash into the microfluidic flow chamber where patterning occurs. By opening the solenoid valve, air above the fluid surface is pressurized, thus forcing fluid into the ink/wash tube. We used ink/wash tubing with as small a diameter as possible to minimize the volume of fluid retained in the tube (and thus wasted). Small-diameter tubing also had a high resistance to flow which helped prevent backflow from one inlet back into another through the microfluidic flow chamber.

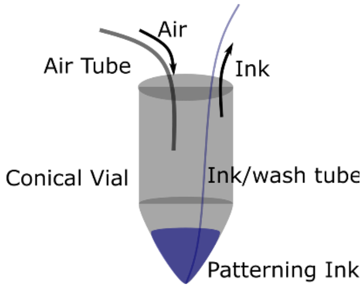

Figure 1. Vial setup and terminology. Pressurized air is fed into the surface of the air-tight vial through the air tube, which forces ink through the ink/wash tube out of the vial and into the microfluidic flow chamber.

4. Protocol for the fabrication of a silicon master mold for the 100 μm tall microfluidic flow chamber

1) Bake 4-inch silicon wafer at 200°C for 10 minutes
2) Plasma treat using Technics PE-II-A Etcher & Stripper at 100W for 5 minutes.
3) Spin coat SU-8 3050 photoresist at 1000 rpm for 30 seconds with an acceleration of 300 rotations/second
4) Bake at 95°C for 45 minutes
5) Using a Teflon mask (**SI Figure 2**) and the double-sided mask aligner, treat the photoresist-covered silicon wafer with 250 mJ/cm^2 UV energy
6) Bake at 95°C for 5 minutes
7) Develop using SU-8 developer until all undeveloped SU-8 is removed
8) Wash with a final rinse of SU-8 developer followed by isopropyl alcohol
9) Dry using compressed air or nitrogen
10) Bake at 200°C overnight

This protocol is adopted from a standard photolithography protocol from the Kayaku Microchem SU-8 3000 Data Sheet[3] (steps 1-10). In addition to following this data sheet at the SU-8 3050 photoresist and 100µm height values (3-7), we treated the wafer with oxygen plasma (2) to ensure we had a clean surface and to increase the bond strength of the photoresist to the wafer. After successful creation of the template, the mold was used for all experiments. The mask to make this mold can be found at

https://github.com/MishaRubanov/MAPDH/tree/main/Supplemental%20protocols%20and%20data. Each mask contains 4 microfluidic flow chambers, and each mold can be reused many times to fabricate new microfluidic flow chambers.

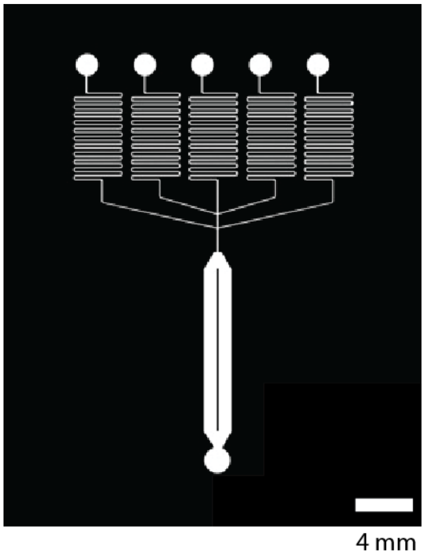

Figure 2. Design of the multi-domain hydrogel patterning flow chamber. This microfluidic flow chamber was designed using AutoCAD. The design was used to generate a mask (5) produced by Fineline Imaging.

This microfluidic flow chamber consists of five inlets each with a resistor network of thin channels following them to increase the pressure drop of flow passing through the channels and decrease the chance of flow back up the channels. These five inlets merge and then branch into two patterning channels each measuring 1mm and 10mm long, separated by a 100µm wide wall. These two channels merge near the outlet.

5. Protocol for fabricating PDMS: Glass microfluidic flow chambers

1) Mix PDMS at a ratio of 1:10 curing agent to elastomer.
2) Pour over silicon wafer mold.
3) Degas for 30 minutes to remove air bubbles using vacuum.
4) Cure at 86°C for 30 minutes.
5) Remove PDMS from the silicon wafer mold.
6) Cut out individual microfluidic flow chambers.
7) Use biopsy punches (0.75mm and 1.5mm) to create inlet and outlet holes depending on which size of tubing is being used.
8) Clean off PDMS surface with scotch tape.
9) Treat surface of glass coverslip and surface of PDMS microfluidic flow chamber with Tesla coil for 15-20 seconds at a medium voltage with the 3-inch wire field-effect electrode.
10) Place treated surface of PDMS on treated surface of glass cover slip.
11) Bake at 86°C for 2 hours.

We create our microfluidic flow chambers out of the silicon polymer polydimethylsiloxane (PDMS), which is a commonly used material for microfluidic fabrication due to its low cost and ease of use. We purchased a kit that includes the elastomer and curing agent (**SI Section 33** for vendor information). We mixed the reagents provided by the kit at a 10:1 ratio of elastomer and curing agent and then poured that mixture over

the mold (steps 1-2). To prevent air bubbles that are generated while mixing the elastomer and curing agent together from causing deformations in the PDMS microfluidic flow chambers, we use a vacuum pump (house vacuum) and vacuum chamber to remove the air in the uncured PDMS, leaving it in the chamber for 30 minutes or until all the gas has risen out of the uncured PDMS (step 3). We also remove remaining bubbles at the surface, if necessary, by carefully dragging them away from the channels on the mold using a pipette tip. Although PDMS will cure at room temperature, we cure it at 86°C for at least 30 minutes (step 4) to speed up the process and create a more elastic final product than curing at room temperature would. The increased elasticity of the microfluidic flow chamber decreases the likelihood of deforming the PDMS microfluidic flow chamber when removing the PDMS from the silicon wafer mold. Using a razor blade, we cut the PDMS from the mold (being careful to not apply too much force on the silicon wafer, which can cause the wafer to break) (step 5), and then cut out the individual microfluidic flow chambers (step 6). We create the channel inlets and outlets using biopsy punches which allows us to remove the PDMS where tubing will be plugged into the microfluidic flow chamber (step 7): 0.75mm for the inlets to accommodate the low volume, high resistance tubing, and 1.5mm for the outlet which uses the larger diameter tubing. After this, we clean the surface of the PDMS using scotch tape (step 8) which removes any dust and debris without leaving a residue behind. The glass slide we use to form the bottom of the microfluidic flow chamber is a coverslip that we can image and photopattern through on an inverted microscope. Before we bond the glass and the PDMS (step 10), we treat them with plasma (step 9) to further clean them and to functionalize their surfaces for enhanced bonding. We then bake the microfluidic flow chambers for at least 2 hours (step 11) to create a permanent bond between the glass and the PDMS. After this, microfluidic flow chambers can be stored indefinitely.

6. Single-domain hydrogel fabrication protocol

The list of steps to run the protocol are shown below, followed by a detailed explanation of the protocol.

1) Turn on all MAPDH hardware, connect to Micromanager and Pycromanager (**SI Section 30**).
2) Insert the waste tube, 24-inch length of Tygon tubing (0.02”x0.06”), into the outlet of the 5-inlet 100μm empty (air-filled) microfluidic chamber.
3) Make four 200μL ink solutions in four vials, each with the following composition:
    - o 10 v/v% PEGDA-575
    - o 1 w/v% LAP
    - o 1X TAEM
    - o 500nM 5Acry_3Cy3_polyT10
4) Add 800 μL of 1X TAEM (wash solution) into a fifth vial.
5) Screw in each modified cap to its corresponding vial to connect all five vials to the five ink/wash tubes that are connected to the flow controller (**SI Section 3**).
6) Set the pressure for each vial on the low-volume flow controller to 3 PSI.
7) To prime the first ink tube, run the ink solution through the first ink tube by opening the first valve on the low-volume flow controller.
8) Using a 0.75mm biopsy punch to assist, insert the ink tube primed in step 7 into the first inlet of the microfluidic chamber.
9) Open the valve, to flow the ink solution through the microfluidic chamber until the ink solution reaches the outlet of the microfluidic chamber, then stop the flow by closing the valve.
10) Repeat steps 7-9 for the solutions in the other four vials, performing these steps for the vial containing the wash solution last.
11) Place the microfluidic flow chamber on the XY Stage, place the vials at a higher height than the microfluidic flow chamber (so that gravity assists rather than impedes flow), and place the end of a

waste tube on the rim of a waste receptacle (i.e., a 15 mL Falcon tube). The waste receptacle should be at the same height as the vials.

12) Focus on the surface of the microfluidic flow chamber with the 10X objective.
13) Clamp the waste tubing and all ink tubes except the wash tube.
14) Increase pressure on the flow controller for the wash vial to 5 PSI. Then open the wash valve and leave the wash valve open until all remaining air is removed from the microfluidic flow chamber. To verify all air is removed, look through the microfluidic flow chamber using the microscope.
15) Return the pressure on the flow controller to 3 PSI and stop the flow of the wash solution by closing the valve.
16) Unclamp the waste, ink, and solution tubes.
17) Move field of view of the camera to the patterning channel within the microfluidic flow chamber and refocus if necessary. Then adjust microscope focus to 50% of a full rotation of the focus knob below the channel focal plane for patterning.
18) Run the MAPDH script linked below three times at 3 different starting locations:
    - MAPDH script
        - https://github.com/MishaRubanov/MAPDH/tree/main/Supplemental%20protocols%20and%20data
    - This MAPDH script patterns 60 different hydrogels (each a 50µm square) in 12 sets (left to right) of 5 (patterned top to bottom) (**SI Figure 4B**).
        - Set 1: 5 squares with Ink 1 (**SI Figure 4A**)
        - Set 2: 5 squares with Ink 2
        - Set 3: 5 squares with Ink 3
        - Set 4: 5 squares with Ink 4
        - Set 5: 5 squares with Ink 1
        - Set 6: 5 squares with Ink 2
        - Set 7: 5 squares with Ink 3
        - Set 8: 5 squares with Ink 4
        - Set 9: 5 squares with Ink 1
        - Set 10: 5 squares with Ink 2
        - Set 11: 5 squares with Ink 3
        - Set 12: 5 squares with Ink 4
19) After patterning is done, flow 1X TAEM wash by opening the wash valve for 5-10 minutes.
20) Take both brightfield and Cy3 micrographs of fabricated hydrogels.

The single-domain hydrogel patterning experiment in **Figure 2** was run using the 5-inlet 100 µm microfluidic flow chamber (**SI Section 4**). After turning on all the necessary equipment and software (step 1) (**SI Section 30**), we set up the flow chamber by inserting the waste tubing first (step 2). The waste tubing had a larger diameter compared to the ink/wash tubing (0.02”x0.06”) to ensure low resistance of the outlet so that fluid prefers to flow through the waste tubing rather than through the ink/wash tubing. 24 inches of tubing was used to ensure that there was enough tubing to reach the waste receptacle.

We made one ink solution containing 500nM of strand 5Acry_3Cy3_polyT10. This solution was then split into four 200µL aliquots each placed in vials (step 3) that are then connected to the low-volume flow controller: each vial had a modified lid (step 5) with one hole through which a hollow point needle connected to an air source (for pressure) was inserted and one hole through which the low volume ink/wash tubing (0.01”x0.03”) fed through to the bottom of the vial for flow of solution from the bottom of the vial to the microfluidic flow chamber (**SI Section 3**). We used this experiment to compare the sizes and fluorescence of hydrogels patterned in different rounds to determine if the round the hydrogel was patterned

in influences how much DNA was anchored inside the hydrogel or hydrogel size (since the vials were otherwise identical). We used small-diameter ink/wash tubing to minimize the volume that was retained in the tubing and to increase the resistance to flow, which helped prevent backflow from one inlet into another.

For each inlet, we set the flow controller pressures to 3 PSI (step 6)—a value chosen to give moderate flow through the microfluidic flow chamber. Before connecting the tube to the microfluidic flow chamber , we primed the tube by opening the valve on the flow controller and letting the tubing fill with liquid until a bead of liquid was at the end of the tube (step 7). The tube for the wash solution was inserted last (*i.e.,* after all of the ink solutions) to ensure no ink remained within the microfluidic flow chamber during the rest of the setup process (step 10). The ink solutions and a waste receptacle were placed at the same level above the stage of the microscope to balance the hydrostatic pressure between the fluid source and the outlet of the tubing, and to drive flow down into the microfluidic flow chamber. After placing the microfluidic chamber on the stage (step 11), we used the 10X objective to focus on the flow chamber channel where we wanted to pattern (step 12) and then checked for air in the whole microfluidic flow chamber by moving the stage around. If air was visible, then we degassed the microfluidic flow chamber. This was done by clamping the waste tube and all the ink tubes except the wash solution tube (step 13), which meant we could increase the pressure inside of the microfluidic flow chamber, since fluid could not move out of the outlet or the other inlets, causing the air to compress and diffuse through the PDMS. After clamping everything except the wash solution tube, we opened the valve to the wash, increased the pressure to the wash vial on the flow controller to 5 PSI, and waited for the air in the microfluidic flow chamber to diffuse out through the PDMS. This usually took a few minutes (step 14).

To prepare for patterning, we focused on the channel of the microfluidic flow chamber through the camera and then moved 50% of a full rotation of the focus knob (*i.e.*, the location of the objective relative to the patterning chamber) below the PDMS: glass interface as focused on by the camera (step 16). The plane that the camera is focused on was lowered to allow the UV light coming from the digital micromirror microfluidic flow chamber to be in focus, which allowed for hydrogels patterned at that focal plane to have well-defined features (**SI Figure 3A**). This is due to the different locations for the DMD and camera on the microscope.

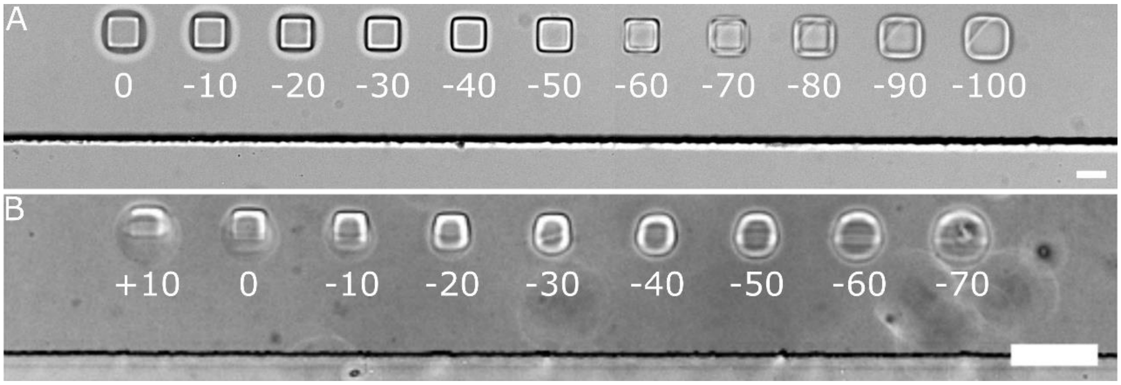

Figure 3. Brightfield micrographs of hydrogels patterned at different z-planes. The labels below each hydrogel are the percentages of a full rotation of the microscope, relative to focusing the camera on the glass surface-PDMS interface that the objective was set to before patterning. Since the DMD and camera are in different locations within the microscope and thus have different planes where they are in focus, we chose the focal plane for patterning by first focusing on the z-plane of the camera, and then adjusting the z-plane to match the ideal plane for patterning. A) 10x patterning and imaging. We observed that the sharpest edges were made when patterning at a plane 50% of a full turn below the plane where the camera is focused on the glass: PDMS interface of the microfluidic flow chamber. B) Patterning and imaging using the 20x objective. We observed that the sharpest edges were found when patterning at a plane 30% of a full turn below the plane where the camera is focused on the glass: PDMS interface of the microfluidic flow chamber. Scale bars 50 µm.

The MAPDH script for this experiment patterns 4 rounds of 5 individual hydrogels in a line from top to bottom in each round (**SI Figure 4**). All hydrogels patterned had the same composition (*i.e.,* domain type). The MAPDH script was run (step 17) three times, each time starting in a new location, for a total of 12 rounds of patterning. To pattern each hydrogel domain, the MAPDH script directs the initial flow of ink into the microfluidic chamber for 90 seconds. Between each patterning step, the current ink solution is flowed for 5 seconds to provide fresh ink to the chamber. After patterning of the entire domain, a wash solution (1x TAEM) is flowed for 60 seconds to remove all previous ink in the chamber. After running the MAPDH script to pattern all four hydrogel domains (step 17), the wash solution is flowed through the microfluidic flow chamber for an additional 5-10 minutes (step 18) to remove any remaining ink solution before imaging (step 19).

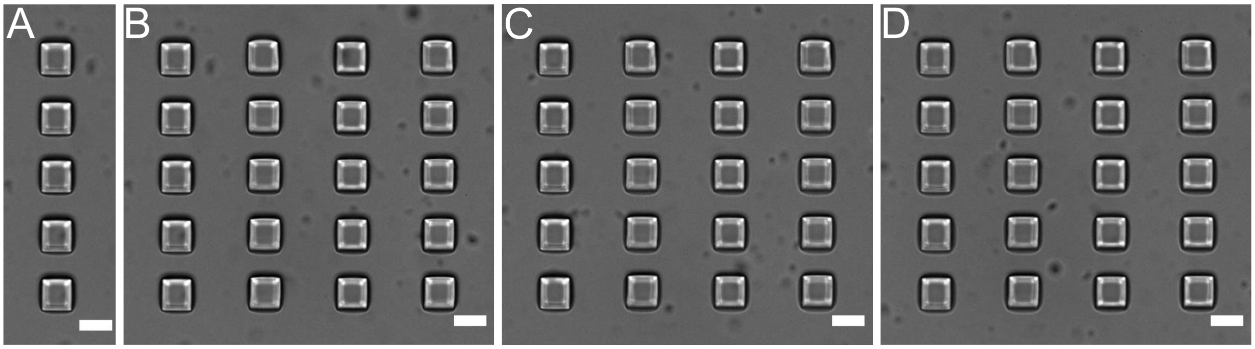

Figure 4. Brightfield micrographs of gels patterned as part of the single-domain hydrogel patterning experiment. The first micrograph shows the hydrogels produced within a single round of patterning (Set 1). The last three micrographs show the resulting hydrogels from all 12 rounds of patterning, in order from left to right. Scale bars are 50 µm.

7. Data and analysis for single-domain hydrogel photopatterning in 100 µm tall flow chamber

After imaging, we overlaid the brightfield and Cy3 micrographs using ImageJ to merge the brightfield micrograph into the gray channel and the Cy3 micrograph into the red channel (**SI Figure 5**).

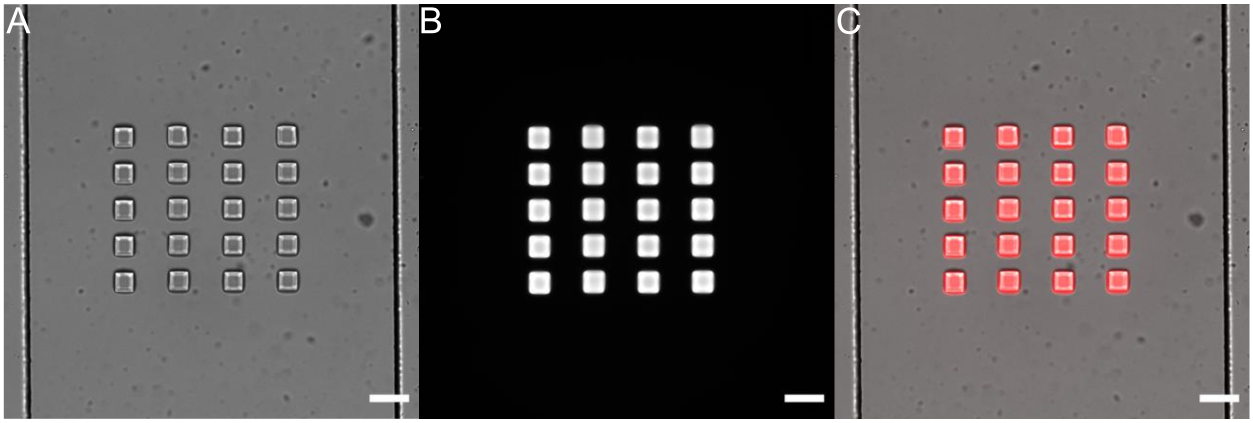

Figure 5. Micrograph overlay using ImageJ. Scale bars are 100 µm. A) Bright field image B) Fluorescence image (Cy3) C) Overlay with brightfield grayscale and Cy3 in red.

We measured the height and width of each gel using the brightfield micrographs. We used the rectangle selection tool to select the outline of the gel within a micrograph (**SI Figure 6A**), and with ImageJ's built-in measure function recorded the height and width of the gel. We then calculated the average fluorescence

intensity within that rectangular area in the Cy3 micrograph (**SI Figure 6B**). This measurement process was repeated for each individual gel.

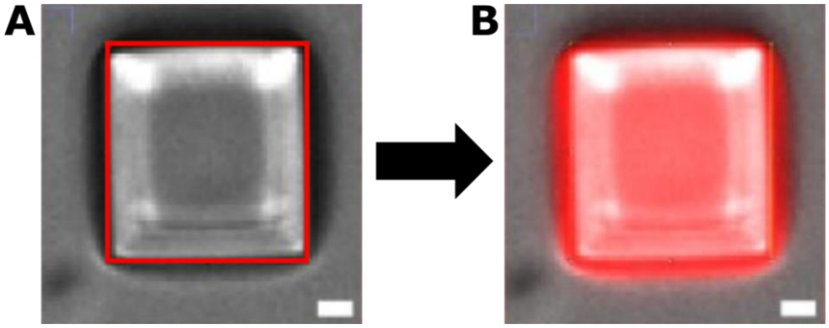

Figure 6. Gel measurements. A) Brightfield micrograph for a single hydrogel. The outline was chosen manually using ImageJ's rectangle selection tool. The width and height of this rectangle corresponded to the width and height of the hydrogel. B) Overlayed brightfield and fluorescent Cy3 micrographs for a single hydrogel. The rectangle, selected from the brightfield micrograph in SI Figure 6A, is used to calculate the average fluorescence intensity for the hydrogel. Scale bars are 10 µm.

The measurements from the 5 gels from each round were used (N = 5 for each round) to find the average and standard deviation for each round of the height, width, and fluorescence of gels patterned in that round that are reported in **Figure 2H, I.**

8. Protocol for the fabrication of a silicon master mold of the 20 µm tall microfluidic flow chamber

1) Bake 4-inch silicon wafer at 200°C for 10 minutes.
2) Plasma treat the wafer using Technics PE-II-A Etcher & Stripper at 100W for 5 minutes.
3) Spin coat SU-8 10 photoresist at 1650 rpm for 30 seconds with an acceleration of 300rotations/second onto the silicon wafer.
4) Bake the wafer at 95°C for 6 minutes.
5) Using a Teflon mask (**SI Figure 2**) and the double-sided mask aligner, treat the photoresist-covered silicon wafer with 200mJ/cm^2 UV energy.
6) Bake the wafer at 95°C for 2.5 minutes.
7) Rinse the baked wafer in SU-8 developer until all undeveloped (i.e., unpolymerized) SU-8 is removed.
8) Wash with a final rinse of SU-8 developer followed by isopropyl alcohol.
9) Dry using compressed air or nitrogen.
10) Bake at 200°C overnight.

Using the mask in **SI Figure 2**, we adapted a standard photolithography protocol from the Kayaku Microchem SU-8 Data Sheet [3] (steps 1-10). In addition to following the protocol on this data sheet for the SU-8 10 photoresist and 20 µm height values (steps 3-7), we treated the wafer with oxygen plasma (step 2) to ensure we had a clean surface and to increase the bond strength of the photoresist to the wafer. After successful creation of the template, the mold produced using this process was used for all the experiments whose results are shown in **SI Figure 7**.

9. Protocol, data, and analysis for MAPDH in 20 µm tall flow chamber

The list of steps to run the protocol are shown below, followed by a detailed explanation of the protocol.

1) Turn on all MAPDH hardware, connect to Micromanager and Pycromanager (**SI Section 30**).

2) Insert the waste tube, 24-inch length of Tygon tubing (0.02”x0.06”), into the outlet of the 5-inlet 100µm empty (air-filled) microfluidic chamber.
3) Make four 200µL ink solutions in four vials, each with the following composition:
   - 10 v/v% PEGDA-575
   - 1 w/v% LAP
   - 1X TAEM
4) Add 800 µL of 1X TAEM (wash solution) into a fifth vial.
5) Screw in each modified cap to its corresponding vial to connect all five vials to the five ink/wash tubes that are connected to the flow controller (**SI Section 3**).
6) Set the pressure for each vial on the low-volume flow controller to 3 PSI.
7) To prime the first ink tube, run the ink solution through the first ink tube by opening the first valve on the low-volume flow controller.
8) Using a 0.75mm biopsy punch to assist, insert the ink tube primed in step 7 into the first inlet of the microfluidic chamber.
9) Open the valve, to flow the ink solution through the microfluidic chamber until the ink solution reaches the outlet of the microfluidic chamber, then stop the flow by closing the valve.
10) Repeat steps 7-9 for the solutions in the other four vials, performing these steps for the vial containing the wash solution last.
11) Place the microfluidic flow chamber on the XY Stage, place the vials at a higher height than the microfluidic flow chamber (so that gravity assists rather than impedes flow), and place the end of a waste tube on the rim of a waste receptacle (i.e., a 15 mL Falcon tube). The waste receptacle should be at the same height as the vials.
12) Focus on the surface of the microfluidic flow chamber with the 20X objective.
13) Clamp the waste tubing and all ink tubes except the wash tube.
14) Increase pressure on the flow controller for the wash vial to 5 PSI. Then open the wash valve and leave the wash valve open until all remaining air is removed from the microfluidic flow chamber. To verify all air is removed, look through the microfluidic flow chamber using the microscope.
15) Return the pressure on the flow controller to 3 PSI and stop the flow of the wash solution by closing the valve.
16) Unclamp the waste, ink, and solution tubes.
17) Move field of view of the camera to the patterning channel within the microfluidic flow chamber and refocus if necessary. Then adjust microscope focus to 50% of a full rotation of the focus knob below the channel focal plane for patterning.
18) Run the MAPDH script linked below three times at 3 different starting locations:
   - MAPDH script
   - https://github.com/MishaRubanov/MAPDH/tree/main/Supplemental%20protocols%20and%20data
   - This MAPDH script patterns 40 different hydrogels (each a 20µm square) in 8 sets (left to right) of 5 (patterned top to bottom) (**SI Figure 7**).

The single domain hydrogel patterning experiment in **SI Figure 7** was run using a 5-inlet 20 µm microfluidic chamber. The microfluidic flow chamber was fabricated at a 20 µm height rather than 100 µm height as we observed that hydrogels with sharper edges formed in the 20 µm height channels from less scattering of light. After turning on all the necessary equipment and software (step 1) (**SI Section 30**), we set up the flow chamber by inserting the waste tubing first (step 2). The waste tubing had a larger diameter compared to the ink/wash tubing (0.02”x0.06”) to ensure low resistance of the outlet so that fluid prefers

to flow through the waste tubing rather than through the ink/wash tubing. 24 inches of tubing was used to ensure that there was enough tubing to reach the waste receptacle.

We made one ink solution, and this solution was then split into four 200µL aliquots each placed in a vial (step 3). This solution was then split into four 200µL aliquots each placed in vials (step 3) that are then connected to the low-volume flow controller: each vial had a modified lid (step 5) with one hole through which a hollow point needle connected to an air source (for pressure) was inserted and one hole through which the low volume ink/wash tubing (0.01”x0.03”) fed through to the bottom of the vial for flow of solution from the bottom of the vial to the microfluidic flow chamber (**SI Section 3**). We used this experiment to compare the sizes of hydrogels patterned in different rounds to determine if the round the hydrogel was patterned in influences hydrogel size (since the vials were otherwise identical). We used small-diameter ink/wash tubing to minimize the volume that was retained in the tubing and to increase the resistance to flow, which helped prevent backflow from one inlet into another.

For each inlet, we set the flow controller pressures to 3 PSI (step 6)—a value chosen to give moderate flow through the microfluidic flow chamber. Before connecting the tube to the microfluidic flow chamber, we primed the tube by opening the valve on the flow controller and letting the tubing fill with liquid until a bead of liquid was at the end of the tube (step 7). The tube for the wash solution was inserted last (*i.e.*, after all of the ink solutions) to ensure no ink remained within the microfluidic flow chamber during the rest of the setup process (step 10). The ink solutions and a waste receptacle were placed at the same level above the stage of the microscope to balance the hydrostatic pressure between the fluid source and the outlet of the tubing, and to drive flow down into the microfluidic flow chamber. After placing the microfluidic chamber on the stage (step 11), we used the 10X objective to focus on the flow chamber channel where we wanted to pattern (step 12) and then checked for air in the whole microfluidic flow chamber by moving the stage around. If air was visible, then we degassed the microfluidic flow chamber. This was done by clamping the waste tube and all the ink tubes except the wash solution tube (step 13), which meant we could increase the pressure inside of the microfluidic flow chamber, since fluid could not move out of the outlet or the other inlets, causing the air to compress and diffuse through the PDMS. After clamping everything except the wash solution tube, we opened the valve to the wash, increased the pressure to the wash vial on the flow controller to 5 PSI, and waited for the air in the microfluidic flow chamber to diffuse out through the PDMS. This usually took a few minutes (step 14).

To prepare for patterning, we focused on the channel of the microfluidic flow chamber through the camera and then moved 30% of a full rotation of the focus knob (*i.e.*, the location of the objective relative to the patterning chamber) below the PDMS: glass interface as focused on by the camera (step 16). The plane that the camera is focused on was lowered to allow the UV light coming from the digital micromirror microfluidic flow chamber to be in focus, which allowed for hydrogels patterned at that focal plane to have well-defined features (**SI Figure 3B**). This is due to the different locations for the DMD and camera on the microscope.

The MAPDH script for this experiment patterns 4 rounds of 5 individual hydrogels in a line from top to bottom each round, first using a 20µm square mask (**SI Figure 7A**) and then using a 10µm mask (**SI Figure 7B**). To pattern each hydrogel domain, the MAPDH script directs the initial flow of ink into the microfluidic chamber for 90 seconds. Between each patterning step, the current ink solution is flowed for 5 seconds to provide fresh ink to the chamber. After patterning of the entire domain, a wash solution (1x TAEM) is flowed for 60 seconds to remove all previous ink in the chamber. After running the MAPDH script to pattern all four hydrogel domains (step 17), the wash solution is flowed through the microfluidic flow chamber for an additional 5-10 minutes (step 18) to remove any remaining ink solution before imaging (step 19).

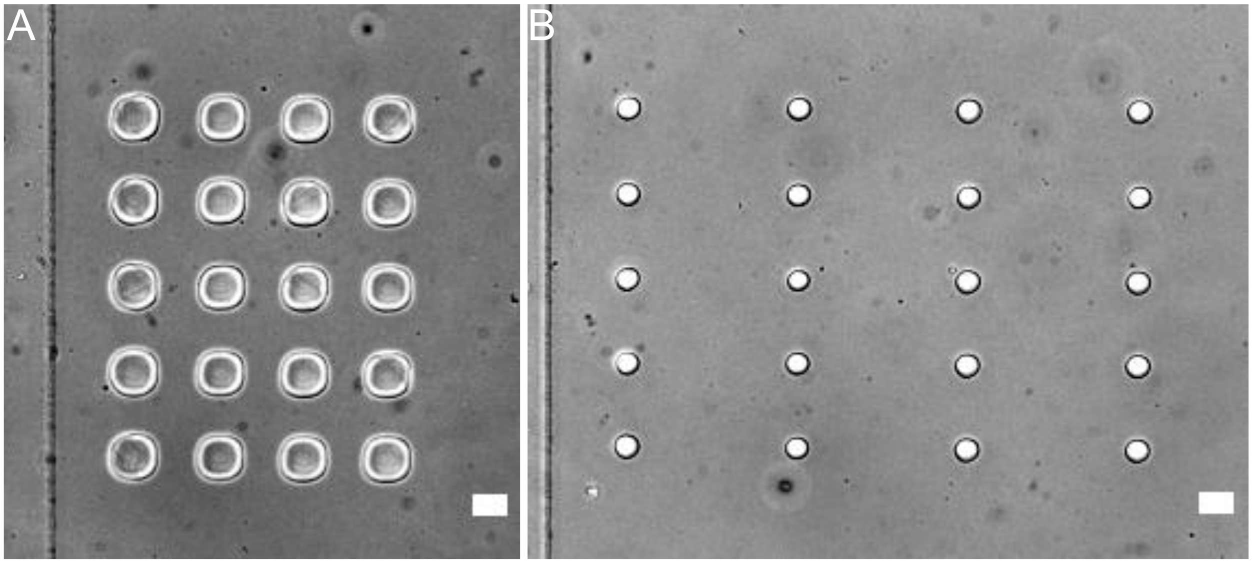

Figure 7. Small gels patterning. A) Gels patterned automatically using 20 µm square masks and Inks 1-4 following the SI Section 9 protocol. B) Gels patterned automatically using 10 µm square masks and inks 1-4 following the SI Section 9 protocol. Scale bars are 20 µm.

The gels were then measured for height and width using the same method in **SI Figure 6**. 20 hydrogels patterned using the 20 µm mask and 20 hydrogels patterned using the 10 µm mask were analyzed for the average and standard deviation of the height and width of gels patterned at using that mask. The hydrogels patterned using a 20 µm rectangular mask have a measured height of 26.88±0.76 µm (mean±s.d.) and a measured width of 27.12±0.71 µm (mean±s.d.). Using MAPDH with a 10µm rectangular mask, the measured height is 13.69±0.76 µm (mean±s.d.) and the measured width is 14.62±0.71 µm (mean±s.d.).

10. Multi-domain hydrogel fabrication protocol

The list of steps to run the protocol are shown below, followed by a detailed explanation of the protocol.

1) Turn on all MAPDH hardware, connect to Micromanager and Pycromanager (**SI Section 30**).
2) Insert the waste tube, 24-inch length of Tygon tubing (0.02”x0.06”), into the outlet of the 5-inlet 100µm empty (air-filled) microfluidic chamber.
3) Make four 200µL ink solutions in four vials, each with the following composition:
    - 10 v/v% PEGDA-575
    - 1 w/v% LAP
    - 1X TAEM
    - Acrydite and fluorophore modified DNA.
        - Ink 1: 500nM 5Acry_3Cy3_polyT10
        - Ink 2: 500nM 5Acry_3ATTO488_polyT10
        - Ink 3: 500nM 5Acry_3TYE665_polyT10
        - Ink 4: 500nM 5Acry_3Cy3_polyT10, 500nM 5Acry_3ATTO488_polyT10, and 500nM 5Acry_3TYE665_polyT10
4) Add 800 µL of 1X TAEM (wash solution) into a fifth vial.
5) Screw in each modified cap to its corresponding vial to connect all five vials to the five ink/wash tubes that are connected to the flow controller (**SI Section 3**).
6) Set the pressure for each vial on the low-volume flow controller to 3 PSI.

7) To prime the first ink tube, run the ink solution through the first ink tube by opening the first valve on the low-volume flow controller.
8) Using a 0.75mm biopsy punch to assist, insert the ink tube primed in step 7 into the first inlet of the microfluidic chamber.
9) Open the valve, to flow the ink solution through the microfluidic chamber until the ink solution reaches the outlet of the microfluidic chamber, then stop the flow by closing the valve.
10) Repeat steps 7-9 for the solutions in the other four vials, performing these steps for the vial containing the wash solution last.
11) Place the microfluidic flow chamber on the XY Stage, place the vials at a higher height than the microfluidic flow chamber (so that gravity assists rather than impedes flow), and place the end of a waste tube on the rim of a waste receptacle (i.e., a 15 mL Falcon tube). The waste receptacle should be at the same height as the vials.
12) Focus on the surface of the microfluidic flow chamber with the 10X objective.
13) Clamp the waste tubing and all ink tubes except the wash tube.
14) Increase pressure on the flow controller for the wash vial to 5 PSI. Then open the wash valve and leave the wash valve open until all remaining air is removed from the microfluidic flow chamber. To verify all air is removed, look through the microfluidic flow chamber using the microscope.
15) Return the pressure on the flow controller to 3 PSI and stop the flow of the wash solution by closing the valve.
16) Unclamp the waste, ink, and solution tubes.
17) Move field of view of the camera to the patterning channel within the microfluidic flow chamber and refocus if necessary. Then adjust microscope focus to 50% of a full rotation of the focus knob below the channel focal plane for patterning.
18) Run the MAPDH script linked below:
    - MAPDH script
        - https://github.com/MishaRubanov/MAPDH/tree/main/Supplemental%20protocols%20and%20data
    - This MAPDH script patterns 80 different hydrogels, each a 50µm square, were patterned in 4 different architectures (**SI Figure 8**)
    - Architecture 1:
        - 5 gels patterned from top to bottom in a line with Ink 1
        - 5 gels patterned from top to bottom in a line with Ink 2
        - 5 gels patterned from top to bottom in a line with Ink 3
        - 5 gels patterned from top to bottom in a line with Ink 4
    - Architecture 2:
        - 1 gel patterned with Ink 1 with 50µm spacing from the other gels.
        - 1 gel patterned with Ink 2 with 50µm spacing from the other gels.
        - 1 gel patterned with Ink 3 with 50µm spacing from the other gels.
        - 1 gel patterned with Ink 4 with 50µm spacing from the other gels.
    - Architecture 3:
        - 4 gels patterned at the corners of a plus shape with Ink 1
        - 4 gels patterned at the corners of a plus shape with Ink 2
        - 4 gels patterned at the corners of a plus shape with Ink 3
        - 4 gels patterned at the corners of a plus shape with Ink 4
    - Architecture 4:
        - 16 gels patterned in a square five gels wide with 150µm spacing with Ink 1
        - 12 gels patterned in a square four gels wide with 150µm spacing with Ink 2

- 8 gels patterned in a square three gels wide with 150µm spacing with Ink 3
- 4 gels patterned in a square two gels wide with 150µm spacing with Ink 4

19) After patterning is done, flow 1X TAEM wash by opening the wash valve for 5-10 minutes.
20) Take micrographs of each set of hydrogels using the Cy3, Atto488, TYE665, and bright-field channels.

The multi-domain hydrogel patterning experiment in **Figure 2** was run using the 5-inlet 100 µm microfluidic flow chamber (**SI Section 4)**. After turning on all the necessary equipment and software (step 1) (**SI Section 30**), we set up the flow chamber by inserting the waste tubing first (step 2). The waste tubing had a larger diameter compared to the ink/wash tubing (0.02"x0.06") to ensure low resistance of the outlet so that fluid prefers to flow through the waste tubing rather than through the ink/wash tubing. 24 inches of tubing was used to ensure that there was enough tubing to reach the waste receptacle.

We made four ink solutions of 200µL each using 3 differently modified strands of DNA each with an acrydite group, a sequence, and a different fluorophore. The first three of these solutions were made using 500nM of one of the following strands: 5Acry_3Cy3_polyT10, 5Acry_3ATTO488_polyT10, or 5Acry_3TYE665_polyT10. The fourth solution was made using all three of these strands at a concentration of 500nM each. We added different fluorescent DNA into each vial so that we could pattern 4 hydrogel domains with distinct fluorescent profiles. Four 200µL aliquots of each ink are placed in vials (step 3) that are then connected to the low-volume flow controller: each vial had a modified lid (step 5) with one hole through which a hollow point needle connected to an air source (for pressure) was inserted and one hole through which the low volume ink/wash tubing (0.01"x0.03") fed through to the bottom of the vial for flow of solution from the bottom of the vial to the microfluidic flow chamber (**SI Section 3**). We used small-diameter ink/wash tubing to minimize the volume that was retained in the tubing and to increase the resistance to flow, which helped prevent backflow from one inlet into another.

For each inlet, we set the flow controller pressures to 3 PSI (step 6)—a value chosen to give moderate flow through the microfluidic flow chamber. Before connecting the tube to the microfluidic flow chamber , we primed the tube by opening the valve on the flow controller and letting the tubing fill with liquid until a bead of liquid was at the end of the tube (step 7). The tube for the wash solution was inserted last (*i.e.,* after all of the ink solutions) to ensure no ink remained within the microfluidic flow chamber during the rest of the setup process (step 10). The ink solutions and a waste receptacle were placed at the same level above the stage of the microscope to balance the hydrostatic pressure between the fluid source and the outlet of the tubing, and to drive flow down into the microfluidic flow chamber. After placing the microfluidic chamber on the stage (step 11), we used the 10X objective to focus on the flow chamber channel where we wanted to pattern (step 12) and then checked for air in the whole microfluidic flow chamber by moving the stage around. If air was visible, then we degassed the microfluidic flow chamber. This was done by clamping the waste tube and all the ink tubes except the wash solution tube (step 13), which meant we could increase the pressure inside of the microfluidic flow chamber, since fluid could not move out of the outlet or the other inlets, causing the air to compress and diffuse through the PDMS. After clamping everything except the wash solution tube, we opened the valve to the wash, increased the pressure to the wash vial on the flow controller to 5 PSI, and waited for the air in the microfluidic flow chamber to diffuse out through the PDMS. This usually took a few minutes (step 14).

To prepare for patterning, we focused on the channel of the microfluidic flow chamber through the camera and then moved 50% of a full rotation of the focus knob (*i.e.*, the location of the objective relative to the patterning chamber) below the PDMS: glass interface as focused on by the camera (step 16). The plane that the camera is focused on was lowered to allow the UV light coming from the digital micromirror microfluidic flow chamber to be in focus, which allowed for hydrogels patterned at that focal plane to have

well-defined features (**SI Figure 3A**). This is due to the different locations for the DMD and camera on the microscope.

The MAPDH script for this experiment patterns 4 rounds of 4 different hydrogel architectures (**SI Figure 8**). To pattern each hydrogel domain, the MAPDH script directs the initial flow of ink into the microfluidic chamber for 90 seconds. Between each patterning step, the current ink solution is flowed for 5 seconds to provide fresh ink to the chamber. After patterning of the entire domain, a wash solution (1x TAEM) is flowed for 60 seconds to remove all previous ink in the chamber. After running the MAPDH script to pattern all four hydrogel domains (step 17), the wash solution is flowed through the microfluidic flow chamber for an additional 5-10 minutes (step 18) to remove any remaining ink solution before imaging (step 19).

11. Micrograph processing for Figure 2J

After imaging, we overlaid the Cy3, ATTO488, and TYE665 micrographs using ImageJ to merge the Cy3 micrograph into the red channel, ATTO488 micrograph into the green channel, and TYE665 micrograph into the blue channel. The brightness and contrast for each channel were set so that the normalized intensity values within each hydrogel were approximately equal in the final composite micrograph.

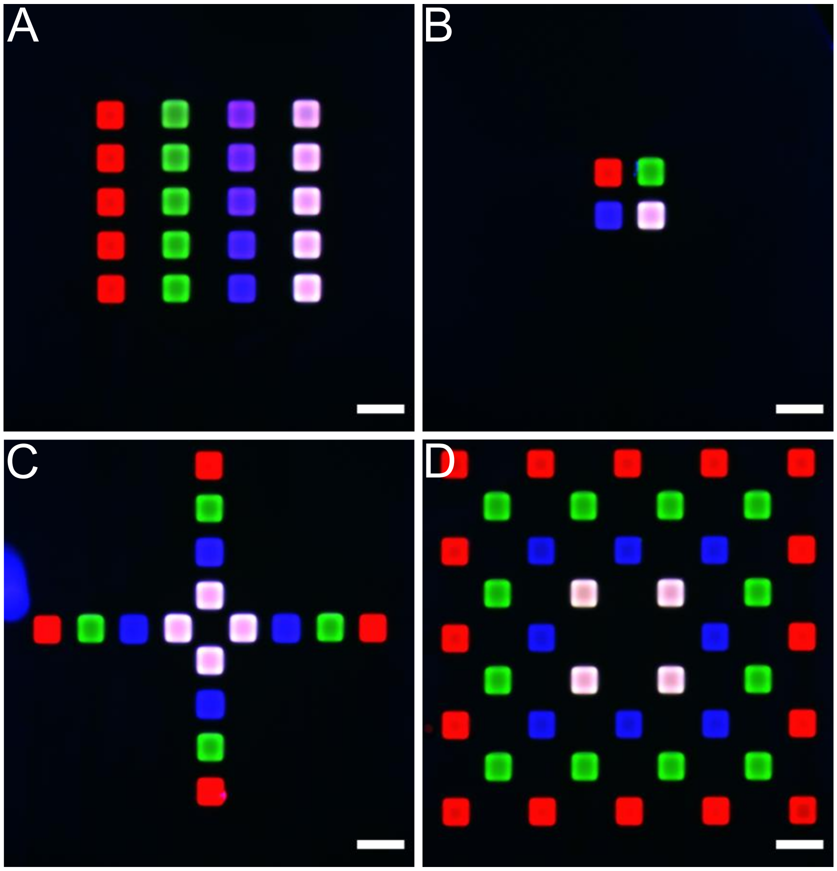

Figure 8. Cy3 (red channel), ATTO488 (green channel), and TYE665 (blue channel) micrographs overlaid showing patterning results from Fig 2J experiment using the protocol in SI Section 10. A) Architecture 1, B) Architecture 2, C) Architecture 3, D) Architecture 4. Scale bars are 100 µm.

12. Location mapping from pixel art

We wanted to develop the ability to convert any pixel art image into a set of instructions for fabricating multi-domain hydrogel architectures. Specifically, we wanted to fabricate the pixel art image as a multi-domain hydrogel architecture, where each pixel is fabricated as a separate hydrogel, and the color of the pixel determines the composition of the hydrogel. To prevent multiple flow steps, we wanted to photopattern each domain (i.e., each color) within the architecture at the same time, before flowing in the following ink. Thus, for a 4-color pixel art image (i.e., a 4-domain hydrogel architecture), we wanted to develop an algorithm that outputs the patterning locations for all hydrogels within each domain. The algorithm we designed outputs four 15x15 binary masks corresponding to the locations for patterning each hydrogel 'pixel' within the hydrogel architecture.

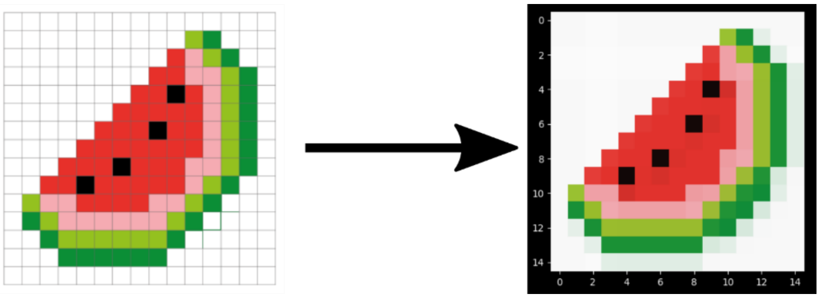

Figure 9. Binning a pixel art image into a 15x15 grid. The left image is a 355x355 pixel image, the right is a binned 15x15 version of the same image.

We first downloaded a .PNG image of a watermelon. The downloaded image is a 355x355 3-channel (RGB) matrix. Initially, we binned the image down to a 15x15 RGB image so that we could create 15x15 binary masks from a 15x15 pixel RGB image (**SI Figure S9**). We chose to automatically cluster the different colors in the image, so that we could use this algorithm to automatically generate patterning instruction for MAPDH for other pixel art images. We used the k-means clustering method to cluster the pixels into 4 color groups (**SI Figure 10**). We then iterate over all pixels and place each pixel into one of 4 masks depending on its color. To determine which mask to place the pixel into, we apply a threshold based on whether the pixel in the image is within 25% the RGB values for one of the extracted colors (red, dark green, light green, or pink). If the pixel passes the thresholding test, the pixel can then be set to 1 within a binary 15x15 mask representing that color (**SI Figure 11**). These masks (termed location maps) are instructions that are used within a function in patterning package for MAPDH (**SI Section 13**).

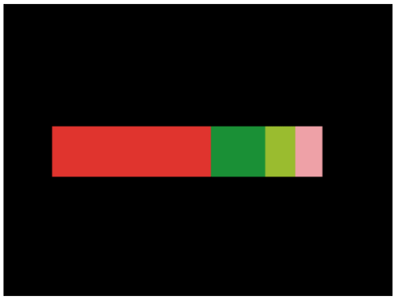

Figure 10. Color histogram for the watermelon pixel art image. The length of each color bar represents the number of pixels with that corresponding color. The colors were clustered automatically using the k-means clustering method.

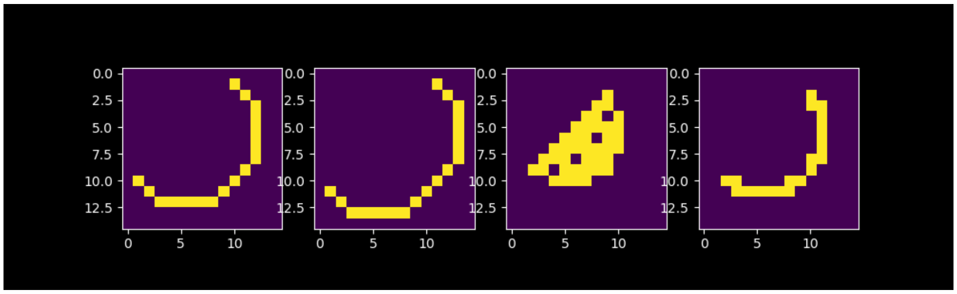

Figure 11. Binary masks for all four domains to be photopatterned using MAPDH. These masks, termed location maps, serve as inputs for the MAPDH function matrix_patterner in SI Section 13.

Code for this section can be found at https://github.com/MishaRubanov/MAPDH/

13. Integration of location maps for use with MAPDH

The 4 binary masks representing locations for patterning each hydrogel domain can then be input into a patterning function found in the patterning functions package:

https://github.com/MishaRubanov/MAPDH

```
def matrix_patterner(mat1,exposure,mask,valveon = [],dist = 60,ch=4,inte=1000):
'''Patterns a grid of hydrogels at location specified in binary matrix mat1.
mat1: locations of each hydrogel in grid.
exposure: UV exposure for all hydrogels.
mask: hydrogel shape.
valveon: turning a particular valve to pattern.
dist: distance between each hydrogel
channel: UV/Blue LED
intensity: LED intensity'''
x=core.getXPosition()
y=core.getYPosition()
for i in range(len(mat1)):
for j in range(len(mat1)):
if mat1[i,j]:
core.setXYPosition(x,y)
time.sleep(3)
core.setRelativeXYPosition((dist)*i,(dist)*j)
time.sleep(2)
if not valveon:
valve_timer(valveon, 5) #flows pregel 5 seconds
patterning(exposure,mask,channel=ch,intensity=inte)
time.sleep(.5)
```

The matrix_patterner function takes as input one binary matrix storing locations for patterning from the previous section, the exposure in seconds, the digital mask for the DMD (the shape of each hydrogel), the valve (or ink) to flow, the distance between each gel (in microns), and the LED type and intensity. The output is the fabrication of a grid of hydrogels at the specified locations. After patterning the first grid for the first domain, a different matrix storing locations for patterning of the next set of hydrogels is input into the matrix_patterner function. After a wash and ink injection step, the matrix_patterner function is then used to pattern the next set of hydrogels.

14. Pixel art multi-domain fabrication protocol

The list of steps to run the protocol are shown below, followed by a detailed explanation of the protocol.

1) Turn on all MAPDH hardware, connect to Micromanager and Pycromanager (**SI Section 30**).
2) Insert the waste tube, 24-inch length of Tygon tubing (0.02”x0.06”), into the outlet of the 5-inlet 100µm empty (air-filled) microfluidic chamber.
3) Make four 200µL ink solutions in four vials, each with the following composition:
   - 10 v/v% PEGDA-575
   - 1 w/v% LAP
   - 1X TAEM
   - Acrydite and fluorophore modified DNA.
     - Ink 1: 500nM 5Acry_3Cy3_polyT10
     - Ink 2: 500nM 5Acry_3ATTO488_polyT10
     - Ink 3: 500nM 5Acry_3TYE665_polyT10
     - Ink 4: 500nM 5Acry_3Cy3_polyT10, 500nM 5Acry_3ATTO488_polyT10, and 500nM 5Acry_3TYE665_polyT10
4) Add 800 µL of 1X TAEM (wash solution) into a fifth vial.
5) Screw in each modified cap to its corresponding vial to connect all five vials to the five ink/wash tubes that are connected to the flow controller (**SI Section 3**).
6) Set the pressure for each vial on the low-volume flow controller to 3 PSI.
7) To prime the first ink tube, run the ink solution through the first ink tube by opening the first valve on the low-volume flow controller.
8) Using a 0.75mm biopsy punch to assist, insert the ink tube primed in step 7 into the first inlet of the microfluidic chamber.
9) Open the valve, to flow the ink solution through the microfluidic chamber until the ink solution reaches the outlet of the microfluidic chamber, then stop the flow by closing the valve.
10) Repeat steps 7-9 for the solutions in the other four vials, performing these steps for the vial containing the wash solution last.
11) Place the microfluidic flow chamber on the XY Stage, place the vials at a higher height than the microfluidic flow chamber (so that gravity assists rather than impedes flow), and place the end of a waste tube on the rim of a waste receptacle (i.e., a 15 mL Falcon tube). The waste receptacle should be at the same height as the vials.
12) Focus on the surface of the microfluidic flow chamber with the 10X objective.
13) Clamp the waste tubing and all ink tubes except the wash tube.
14) Increase pressure on the flow controller for the wash vial to 5 PSI. Then open the wash valve and leave the wash valve open until all remaining air is removed from the microfluidic flow chamber. To verify all air is removed, look through the microfluidic flow chamber using the microscope.
15) Return the pressure on the flow controller to 3 PSI and stop the flow of the wash solution by closing the valve.
16) Unclamp the waste, ink, and solution tubes.
17) Move field of view of the camera to the patterning channel within the microfluidic flow chamber and refocus if necessary. Then adjust microscope focus to 50% of a full rotation of the focus knob below the channel focal plane for patterning.
18) Run the MAPDH script linked below:
   - Patterning MAPDH script
     - https://github.com/MishaRubanov/MAPDH/tree/main/SI%20Protocols/SI%20Section%2014

- This MAPDH script patterns 99 different hydrogels, each a 50µm square, were patterned in watermelon formation based off a pixel art image (**Figure 3**)

19) After patterning is done, flow 1X TAEM wash by opening the wash valve for 5-10 minutes.
20) Take micrographs of each set of hydrogels using the Cy3, Atto488, TYE665, and bright-field channels.

The multi-domain hydrogel patterning experiment in **Figure 3** was run using the 5-inlet 100 um microfluidic chamber (**SI Section 4)**. After turning on all the necessary equipment and software (step 1) (**SI Section 30**), we set up the flow chamber by inserting the waste tubing first (step 2). The waste tubing had a larger diameter compared to the ink/wash tubing (0.02”x0.06”) to ensure low resistance of the outlet so that fluid prefers to flow through the waste tubing rather than through the ink/wash tubing. 24 inches of tubing was used to ensure that there was enough tubing to reach the waste receptacle.

We made four ink solutions of 200µL each using 3 differently modified strands of DNA each with an acrydite group, a sequence, and a different fluorophore. Three of these solutions were made using 500nM of one of the following strands: 5Acry_3Cy3_polyT10, 5Acry_3ATTO488_polyT10, or 5Acry_3TYE665_polyT10. A fourth solution was made using all three of these strands at a concentration of 500nM each. We did this so that we could pattern hydrogels with 4 different fluorescent signatures. Each ink was made at a quantity of 200µL to minimize the amount of material used and placed in a vial (step 3) that would connect to the low volume flow controller setup by screwing the new vial into a modified lid (step 5) that had one hole with a hollow point needle connected to an air source (for pressure) and one hole with the low volume ink/wash tubing (0.01”x0.03”) fed through to the bottom of the vial (for flow of solution). We used a smaller diameter ink/wash tubing to decrease the volume that was retained in the tubing and to increase the resistance to flow which helped prevent backflow from one inlet into another.

We made four ink solutions of 200µL each using 3 differently modified strands of DNA each with an acrydite group, a sequence, and a different fluorophore. The first three of these solutions were made using 500nM of one of the following strands: 5Acry_3Cy3_polyT10, 5Acry_3ATTO488_polyT10, or 5Acry_3TYE665_polyT10. The fourth solution was made using all three of these strands at a concentration of 500nM each. We added different fluorescent DNA into each vial so that we could pattern 4 hydrogel domains with distinct fluorescent profiles. Four 200µL aliquots of each ink are placed in vials (step 3) that are then connected to the low-volume flow controller: each vial had a modified lid (step 5) with one hole through which a hollow point needle connected to an air source (for pressure) was inserted and one hole through which the low volume ink/wash tubing (0.01”x0.03”) fed through to the bottom of the vial for flow of solution from the bottom of the vial to the microfluidic flow chamber (**SI Section 3**). We used small-diameter ink/wash tubing to minimize the volume that was retained in the tubing and to increase the resistance to flow, which helped prevent backflow from one inlet into another.

For each inlet, we set the flow controller pressures to 3 PSI (step 6)—a value chosen to give moderate flow through the microfluidic flow chamber. Before connecting the tube to the microfluidic flow chamber, we primed the tube by opening the valve on the flow controller and letting the tubing fill with liquid until a bead of liquid was at the end of the tube (step 7). The tube for the wash solution was inserted last (*i.e.,* after all of the ink solutions) to ensure no ink remained within the microfluidic flow chamber during the rest of the setup process (step 10). The ink solutions and a waste receptacle were placed at the same level above the stage of the microscope to balance the hydrostatic pressure between the fluid source and the outlet of the tubing, and to drive flow down into the microfluidic flow chamber. After placing the microfluidic chamber on the stage (step 11), we used the 10X objective to focus on the flow chamber channel where we wanted to pattern (step 12) and then checked for air in the whole microfluidic flow chamber by moving the stage around. If air was visible, then we degassed the microfluidic flow chamber. This was done by

clamping the waste tube and all the ink tubes except the wash solution tube (step 13), which meant we could increase the pressure inside of the microfluidic flow chamber, since fluid could not move out of the outlet or the other inlets, causing the air to compress and diffuse through the PDMS. After clamping everything except the wash solution tube, we opened the valve to the wash, increased the pressure to the wash vial on the flow controller to 5 PSI, and waited for the air in the microfluidic flow chamber to diffuse out through the PDMS. This usually took a few minutes (step 14).

To prepare for patterning, we focused on the channel of the microfluidic flow chamber through the camera and then moved 50% of a full rotation of the focus knob (*i.e.*, the location of the objective relative to the patterning chamber) below the PDMS: glass interface as focused on by the camera (step 16). The plane that the camera is focused on was lowered to allow the UV light coming from the digital micromirror microfluidic flow chamber to be in focus, which allowed for hydrogels patterned at that focal plane to have well-defined features (**SI Figure 3A**). This is due to the different locations for the DMD and camera on the microscope.

The patterning MAPDH script reads 4 location maps (i.e., binary masks) containing relative locations for patterning each hydrogel domain (**SI Sections 12,13 and Figure 11**). To pattern each hydrogel domain, the MAPDH script directs the initial flow of ink into the microfluidic chamber for 90 seconds. Between each patterning step, the current ink solution is flowed for 5 seconds to provide fresh ink to the chamber. After patterning of the entire domain, a wash solution (1x TAEM) is flowed for 60 seconds to remove all previous ink in the chamber. After running the MAPDH script to pattern all four hydrogel domains (step 17), the wash solution is flowed through the microfluidic flow chamber for an additional 5-10 minutes (step 18) to remove any remaining ink solution before imaging (step 19).

15. Micrograph Processing for Figure 3

We initially imaged the 4-domain hydrogel architecture using 3 fluorophore channels: Cy3, Atto488, TYE665. After imaging, we processed the micrographs first using automatic contrast enhancement using histogram equalization. After contrast enhancement, we applied a colormap to each grayscale micrograph depending on the binary mask used to make it. For example, the binary mask used to make the red part of the hydrogel architecture (shown first in **SI Figure 12**) had a red colormap applied to the micrograph.

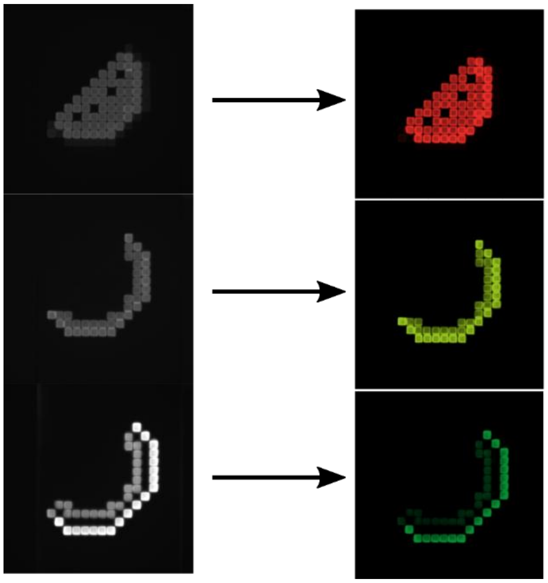

Figure 12. Coloring grayscale micrographs based on original pixel-art colors.

Next, we isolated the 4th domain (the pink domain, which is the domain containing all three different fluorophores within the ink) by thresholding each micrograph using a manually chose threshold to identify pixels within the micrograph containing hydrogels of the corresponding color. We then identify the pixels that were '1' in all three micrographs to produce a new binding mask that is the intersection of the three micrographs. This mask was the locations for the pink domain and is then applied to the first grayscale micrograph in **SI Figure 13** to extract the fourth domain. The resulting grayscale micrograph then has a pink colormap applied to it.

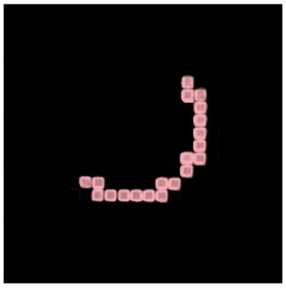

Figure 13. Multi-fluorophore domain. Enhanced and colorized.

The final step is to superimpose all 4 colored micrographs to visualize the final 4-domain pixel art hydrogel shown in **Figure 3D.**

16. MAPDH with addressable hybridization protocol

The list of steps to run the protocol are shown below, followed by a detailed explanation of the protocol.

1) Turn on all MAPDH hardware, connect to Micromanager and Pycromanager (**SI Section 30**).
2) Insert the waste tube, 24-inch length of Tygon tubing (0.02"x0.06"), into the outlet of the 5-inlet 100µm empty (air-filled) microfluidic chamber.
3) Make four 200µL ink solutions in four vials, each with the following composition:
    - 10 v/v% PEGDA-575
    - 1 w/v% LAP
    - 1X TAEM
    - Acrydite and fluorophore modified DNA.
        - Ink 1: 500nM of 5Acry_3Cy3_R1
        - Ink 2: 500nM 5Acry_3ATTO488_polyT10
        - Ink 3: 500nM 5Acry_3TYE665_polyT10
        - Ink 4: 500nM 5Acry_3Cy3_R1, 500nM 5Acry_3ATTO488_polyT10, and 500nM 5Acry_3TYE665_polyT10
4) Add 800 µL of 1X TAEM (wash solution) into a fifth vial.
5) Screw in each modified cap to its corresponding vial to connect all five vials to the five ink/wash tubes that are connected to the flow controller (**SI Section 3**).
6) Set the pressure for each vial on the low-volume flow controller to 3 PSI.
7) To prime the first ink tube, run the ink solution through the first ink tube by opening the first valve on the low-volume flow controller.
8) Using a 0.75mm biopsy punch to assist, insert the ink tube primed in step 7 into the first inlet of the microfluidic chamber.
9) Open the valve, to flow the ink solution through the microfluidic chamber until the ink solution reaches the outlet of the microfluidic chamber, then stop the flow by closing the valve.

10) Repeat steps 7-9 for the solutions in the other four vials, performing these steps for the vial containing the wash solution last.
11) Place the microfluidic flow chamber on the XY Stage, place the vials at a higher height than the microfluidic flow chamber (so that gravity assists rather than impedes flow), and place the end of a waste tube on the rim of a waste receptacle (i.e., a 15 mL Falcon tube). The waste receptacle should be at the same height as the vials.
12) Focus on the surface of the microfluidic flow chamber with the 10X objective.
13) Clamp the waste tubing and all ink tubes except the wash tube.
14) Increase pressure on the flow controller for the wash vial to 5 PSI. Then open the wash valve and leave the wash valve open until all remaining air is removed from the microfluidic flow chamber. To verify all air is removed, look through the microfluidic flow chamber using the microscope.
15) Return the pressure on the flow controller to 3 PSI and stop the flow of the wash solution by closing the valve.
16) Unclamp the waste, ink, and solution tubes.
17) Move field of view of the camera to the patterning channel within the microfluidic flow chamber and refocus if necessary. Then adjust microscope focus to 50% of a full rotation of the focus knob below the channel focal plane for patterning.
18) Run MAPDH script.
    - MAPDH script
        - https://github.com/MishaRubanov/MAPDH/tree/main/Supplemental%20protocols%20and%20data
    - This MAPDH script patterns 16 different hydrogels in 4 sets of 4 hydrogels each at different shapes, locations, and DNA compositions.
        - Set 1
            - 100µm wide square with Ink 1
            - 100µm diameter circle with Ink 2
            - 100µm base triangle with Ink 3
            - 100µm tall/40µm wide plus sign with Ink 4
        - Set 2
            - 100µm wide square with Ink 4
            - 100µm diameter circle with Ink 1
            - 100µm base triangle with Ink 2
            - 100µm tall/40µm wide plus sign with Ink 3
        - Set 3
            - 100µm wide square with Ink 3
            - 100µm diameter circle with Ink 4
            - 100µm base triangle with Ink 1
            - 100µm tall/40µm wide plus sign with Ink 2
        - Set 4
            - 100µm wide square with Ink 2
            - 100µm diameter circle with Ink 3
            - 100µm base triangle with Ink 4
            - 100µm tall/40µm wide plus sign with Ink 1
19) After patterning is done, flow 1X TAEM wash by opening the wash valve for 5-10 minutes.
20) Take micrographs of each set of hydrogels using the Cy3, Atto488, TYE665, and bright-field channels.

21) Remove outlet tubing and flow 1X TAEM wash through microfluidic flow chamber until a bead of liquid rests on the outlet of the microfluidic flow chamber.
22) Move ink/wash tubes to a position below the microscope stage.
23) Insert new Tygon tubing (0.02”x0.06”) into the outlet that is primed and connected to a 1mL syringe with 800µL of a 500nM 5Q_R1’ in 1X TAEM.
24) Image every 10 minutes with the same settings as step 20, and after the first micrograph set is taken, manually flow 750µL of the 500nM 5Q_R1’ in 1X TAEM solution through microfluidic flow chamber by slowly depressing the plunger of the syringe. Continue imaging every 10 minutes.

The reaction-diffusion experiment whose results are shown in **Figure 4** were run in a 5-inlet 100 um microfluidic chamber (**SI Section 4)**. After turning on all the necessary equipment and software (step 1) (**SI Section 30**), we set up the flow chamber by inserting the waste tubing first (step 2). The waste tubing had a larger diameter compared to the ink/wash tubing (0.02”x0.06”) to ensure low resistance of the outlet so that fluid prefers to flow through the waste tubing rather than through the ink/wash tubing. 24 inches of tubing was used to ensure that there was enough tubing to reach the waste receptacle.

We made four ink solutions of 200µL each using 3 differently modified strands of DNA each with an acrydite group, a sequence, and a different fluorophore. The first three of these solutions were made using 500nM of one of the following strands: 5Acry_3Cy3_R1, 5Acry_3ATTO488_polyT10, or 5Acry_3TYE665_polyT10. The fourth solution was made using all three of these strands at a concentration of 500nM each. We added different fluorescent DNA into each vial so that we could pattern 4 hydrogel domains with distinct fluorescent profiles. Four 200µL aliquots of each ink are placed in vials (step 3) that are then connected to the low-volume flow controller: each vial had a modified lid (step 5) with one hole through which a hollow point needle connected to an air source (for pressure) was inserted and one hole through which the low volume ink/wash tubing (0.01”x0.03”) fed through to the bottom of the vial for flow of solution from the bottom of the vial to the microfluidic flow chamber (**SI Section 3**). We used small-diameter ink/wash tubing to minimize the volume that was retained in the tubing and to increase the resistance to flow, which helped prevent backflow from one inlet into another.

For each inlet, we set the flow controller pressures to 3 PSI (step 6)—a value chosen to give moderate flow through the microfluidic flow chamber. Before connecting the tube to the microfluidic flow chamber , we primed the tube by opening the valve on the flow controller and letting the tubing fill with liquid until a bead of liquid was at the end of the tube (step 7). The tube for the wash solution was inserted last (*i.e.,* after all of the ink solutions) to ensure no ink remained within the microfluidic flow chamber during the rest of the setup process (step 10). The ink solutions and a waste receptacle were placed at the same level above the stage of the microscope to balance the hydrostatic pressure between the fluid source and the outlet of the tubing, and to drive flow down into the microfluidic flow chamber. After placing the microfluidic chamber on the stage (step 11), we used the 10X objective to focus on the flow chamber channel where we wanted to pattern (step 12) and then checked for air in the whole microfluidic flow chamber by moving the stage around. If air was visible, then we degassed the microfluidic flow chamber. This was done by clamping the waste tube and all the ink tubes except the wash solution tube (step 13), which meant we could increase the pressure inside of the microfluidic flow chamber, since fluid could not move out of the outlet or the other inlets, causing the air to compress and diffuse through the PDMS. After clamping everything except the wash solution tube, we opened the valve to the wash, increased the pressure to the wash vial on the flow controller to 5 PSI, and waited for the air in the microfluidic flow chamber to diffuse out through the PDMS. This usually took a few minutes (step 14).

To prepare for patterning, we focused on the channel of the microfluidic flow chamber through the camera and then moved 50% of a full rotation of the focus knob (*i.e.*, the location of the objective relative to the patterning chamber) below the PDMS: glass interface as focused on by the camera (step 16). The plane that the camera is focused on was lowered to allow the UV light coming from the digital micromirror microfluidic flow chamber to be in focus, which allowed for hydrogels patterned at that focal plane to have well-defined features (**SI Figure 3A**). This is due to the different locations for the DMD and camera on the microscope.

The MAPDH script for this experiment patterns 4 different hydrogel architectures (**SI Figure 14**). To pattern each hydrogel domain, the MAPDH script directs the initial flow of ink into the microfluidic chamber for 90 seconds. Between each patterning step, the current ink solution is flowed for 5 seconds to provide fresh ink to the chamber. After patterning of the entire domain, a wash solution (1x TAEM) is flowed for 60 seconds to remove all previous ink in the chamber. After running the MAPDH script to pattern all four hydrogel domains (step 17), the wash solution is flowed through the microfluidic flow chamber for an additional 5-10 minutes (step 18) to remove any remaining ink solution before imaging (step 19).

After imaging, we moved the inlet vials to a position below the stage (step 22) so that during the hybridization experiment we could be sure that ink solutions were not flowing into the microfluidic flow chamber. We removed the outlet tubing (step 21) so that a syringe could be connected containing a 500nM solution of the strand 5Q_R1' (step 23), which is complementary to the strand 5Acry_3Cy3_R1 and upon binding quenches its Cy3 fluorophore. We then manually flowed the solution containing 5Q_R1' into the microfluidic flow chamber from the outlet by slowly depressing the plunger of the syringe, which allowed the 5Q_R1' strand to enter the solution surrounding the hydrogels, and thus diffuse into the hydrogels and bind to the 5Acry_3Cy3_R1 strand. We imaged the hydrogels before and after (at 10-minute intervals) injecting the solution of 5Q_R1' to compare fluorescence values between gels before and after hybridization (step 24).

17. Figure 4 micrograph processing

After imaging, we overlaid the Cy3, ATTO488, and TYE665 micrographs using ImageJ. We assigned the Cy3 micrograph to the green channel, the ATTO488 micrograph to the blue channel, and the TYE665 micrograph to the red channel. The brightness and contrast for each channel were manually adjusted so that the intensity values of each hydrogel were approximately equal in the final composite micrograph.

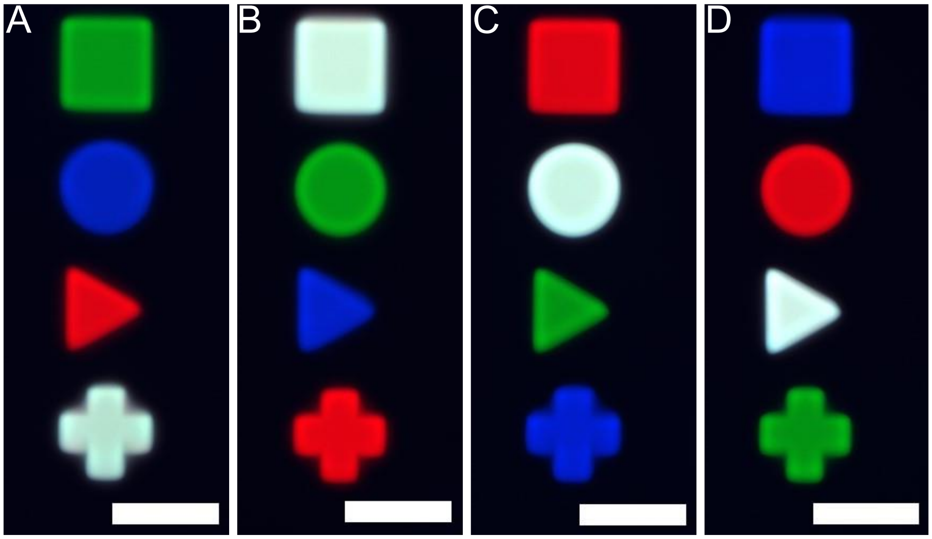

Figure 14. Hydrogels after patterning using the protocol in SI Section16, but before adding the strand 5Q_R1'. The TYE665 (red channel), Cy3 (green channel), and ATTO488 (blue channel) micrographs are overlaid as described in SI Section 17. A) Set 1 B) Set 2 C) Set 3 D) Set 4 as described in SI Section 16. Scale bars are 100 µm.

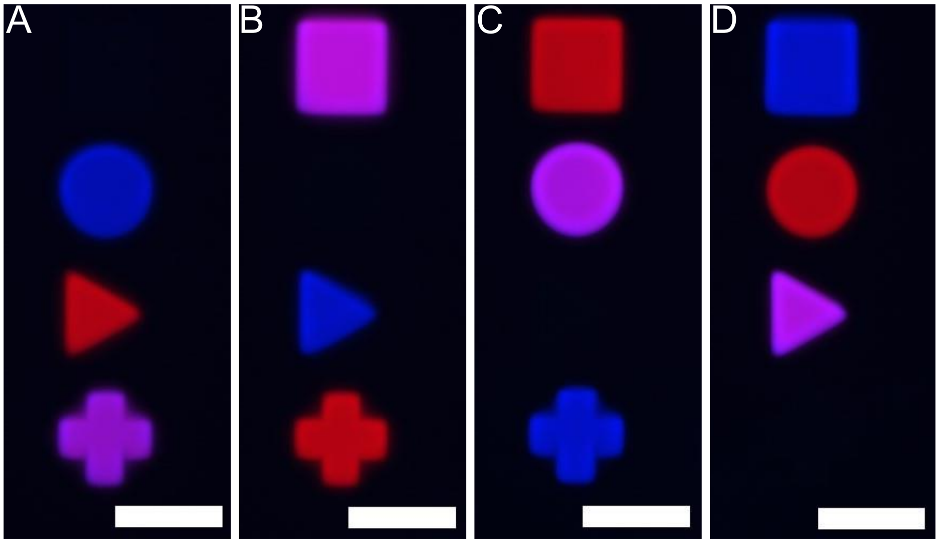

Figure 15. Hydrogels after patterning and adding the strand 5Q_R1' following the protocol described in SI Section 17. TYE665 (red channel), Cy3 (green channel), and ATTO488 (blue channel) micrographs overlaid as described in SI Section 17. A) Set 1 B) Set 2 C) Set 3 D) Set 4 as described in SI Section 16. Scale bars are 100 µm.

We also measured the fluorescence intensities of micrographs taken at 10-minute intervals to track the time scale of changes in fluorescence. The red (TYE665) and blue (ATTO488) fluorescence intensities remained unchanged, while green (Cy3) was quenched within 10 minutes (**SI Figure 16**).

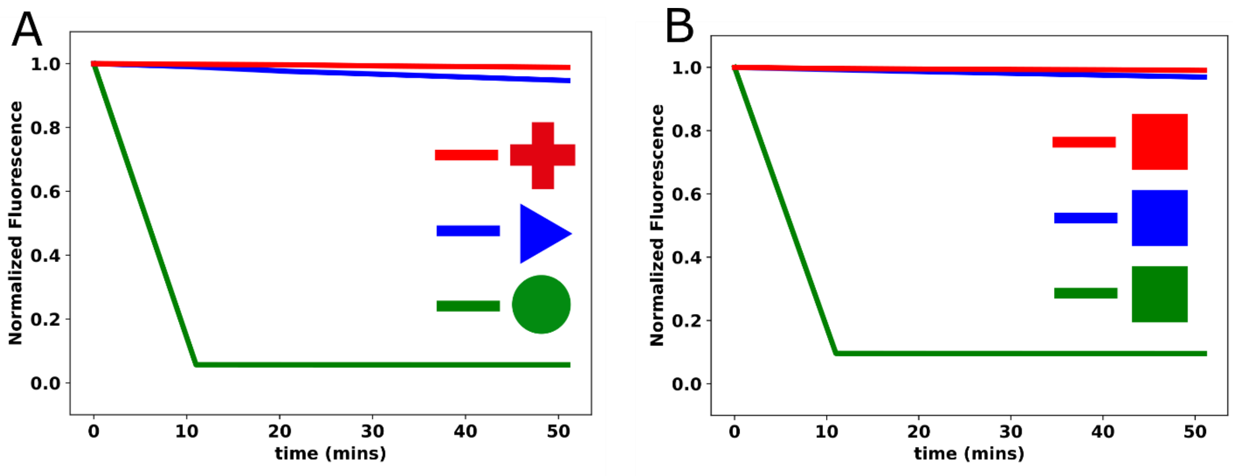

Figure 16. Normalized, mean fluorescence counts for hydrogels in Fig 4 and SI Figs 14B and 15B. A) Normalized fluorescence for the plus, triangle, and circle hydrogels in the respective channels that contained fluorescence. B) Normalized fluorescence for all three fluorophore channels for the square hydrogel.

18. Protocol for fabricating the silicon mold for the sacrificial layer boat

1. Bake a 4-inch silicon wafer at 200°C for 10 minutes.
2. Plasma-treat the wafer using Technics PE-II-A Etcher & Stripper at 100W for 5 minutes.
3. Spin coat SU-8 3050 photoresist initially at 500 rpm for 5 seconds, followed by 1700 RPM for 30 seconds with 300 RPM/sec acceleration onto the wafer.
4. Bake at 95°C for 5 minutes.
5. Using the film mask and double-sided mask aligner, apply 120 mJ/cm^2 of UV (mercury lamp: 200-450 nm) to the photoresist-covered silicon wafer.
6. Bake at 95°C for 5 minutes.
7. Immerse the wafer in SU-8 developer for ~ 5 mins until all unexposed SU-8 is removed.
8. Wash with a final rinse of SU-8 developer followed by isopropyl alcohol.
9. Dry using compressed air or nitrogen.
10. Bake at 200°C overnight.

Masks for the sacrificial boat were designed using AutoCAD and generated via an outside vendor (Fineline Imaging) (**SI Figure 17**).

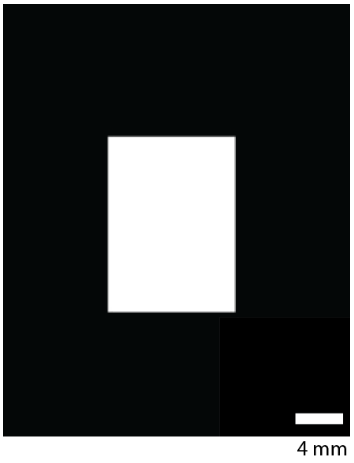

Figure 17. CAD Mask design for the PDMS boat.

SU-8 photoresist molds for the sacrificial layer boat were fabricated on 4-inch silicon wafers with standard contact photolithography (step 1) [4]. We plasma-treated the wafer first to ensure that the photoresist bonded to the wafer (step 2). Molds for the boat PAA sacrificial layer had a target height of 20 µm to be achieved by spin-coating SU-8 10 (step 3) [3] . We chose a target height of 20 mm to ensure robust coating of the sacrificial layer within the PDMS boat without significantly affecting the transmittance of light during MAPDH photopatterning through the boat, since the boat will be the bottom of a microfluidic flow chamber (**Figure 5B**). The wafer was then exposed to UV, baked, washed in SU-8 developer and dried to remove all unexposed photoresist (steps 5-9). Finally, the sacrificial boat was thermally cured in an oven overnight for permanent formation of the sacrificial layer within the boat (step 10).

Each boat containing the sacrificial layer has a length of 14 mm and a width of 10.1mm. The width of the boat is significantly larger than the width of the microfluidic chamber, which has a length of 15 mm and a width of 2mm. When designing the boat, we chose a length that is close to the length of the microfluidic chamber, but a width much wider than the microfluidic chamber ensures that the patterning chamber within the microfluidic flow chamber is in contact with the sacrificial layer **(Figure 4B)**.

19. Protocol for fabricating the sacrificial layer within the PDMS boat

1. Mix 10 g of Dow Sylgard 184 PDMS and 1 g curing agent.
2. Pour the PDMS over the silicon wafer mold of the sacrificial boat.
3. Degas the microfluidic flow chamber for 30 minutes to remove air bubbles.
4. Bake at 80°C for 2 hours.
5. Remove the PDMS from the silicon wafer mold.
6. Cut out individual PDMS boats.
7. Fully dissolve $CaCl_2$ in distilled water to make a 1 M 5mL $CaCl_2$ solution.
8. Prepare 5 mL 5% (v/v) polyacrylic acid (PAA) solution as the sacrificial layer solution.
9. Apply ~ 10 mL of the 5% PAA solution onto each PDMS boat. Use a flat blade to spread the solution evenly.
10. Immediately pipette approximately 20 mL of the 1M $CaCl_2$ solution over the sacrificial layer to completely cover the PAA solution. Use a flat blade to spread the solution evenly.
11. After 5 minutes, wash the sacrificial boats by pipetting approximately 200 mL water over the boat.
12. Gently pipette-mix the liquid above the surface of the sacrificial layer and then pipette out the excess unpolymerized liquid.
13. Thermally cure the PDMS boat with the polymerized sacrificial layer for 10 mins at 150 ^{o}C.

We first developed a protocol to create a microfluidic flow chamber with a removable sacrificial layer embedded within a PDMS boat. This sacrificial layer serves as the base of the patterning chamber (in contrast with other MAPDH protocols, where glass is the base). The boat was created by first pouring mixed PDMS and curing agent over the sacrificial boat mold, then degassing, baking, and removing the microfluidic flow chamber from the mold (steps 1-6). We used established methods and concentrations for sacrificial layer formulation [5,6] (steps 7-8). After pipetting the PAA solution into the boat, we added excess $CaCl_2$ solution over the evenly spread sacrificial layer solution to ensure polymerization of the surface (10). After pouring in the sacrificial layer and waiting a few minutes for curing, we wash the boat to remove excess $CaCl_2$ and uncured PAA using water (step 11). To remove excess PAA from the surface of the PDMS boat, there are two methods that we generally use. Either we run a small blade over the surface of the PDMS, which removes any excess PAA solution above the surface of the PDMS, or we pipette out small aliquots of uncured PAA above the surface of the PDMS. We chose to use pipetting for this part of the protocol to prevent PAA from spreading to other parts of the boat, preventing the PDMS channel from

attaching in the subsequent protocols. We then thermally cure the PDMS boat to finalize the preparation of the sacrificial layer (step 12).

20. Protocol for fabricating lift-off flow chambers

1. Punch holes in the 100 µm PDMS microfluidic chamber prepared using the protocol in **SI Section 5** – using hole punches with diameter 0.75mm for the inlets and 1.5mm for the outlet.
2. Place the PDMS microfluidic chamber and the sacrificial layer boat on a flat surface with their interiors facing upwards.
3. Treat the surface of the sacrificial layer and the surface of the PDMS microfluidic chamber with the Tesla coil for 15-20 seconds.
4. Assemble the sacrificial layer and the patterning microfluidic chamber by firmly pressing the two exposed surfaces together.
5. Anneal the assembled structure in an oven for 1 hour at 90 ^{o}C.

To combine the sacrificial layer with the patterning chamber, the chamber was first hole-punched using a biopsy punch (0.75mm) to form inlets and outlets that fit the appropriate low-volume inlet (0.01” x 0.03”) and outlet Tygon tubing (0.02” x 0.06”) (step 1). The two interior surfaces were then exposed to oxygen plasma to introduce silanol groups (SiOH) to the PDMS surfaces (steps 2-3). The patterning chamber and sacrificial layer are then pressed together, creating Si-O-Si bonds at their interface (step 4). We sandwiched the two PDMS components so that the sacrificial layer fully covers the patterning chamber so that when we pattern, the gels are all anchored on the surface of the sacrificial layer.

21. Protocol for single-domain hydrogel fabrication and liftoff

The list of steps to run the protocol are shown below, followed by a detailed explanation of the protocol.

1) Turn on all MAPDH hardware, connect to Micromanager and Pycromanager (**SI Section 30**).
2) Insert the waste tube, 24-inch length of Tygon tubing (0.02”x0.06”), into the outlet of the 5-inlet 100µm empty (air-filled) lift-off flow chamber.
3) Make a 200µL ink solution in one vial with the following composition:
    - 10 v/v% PEGDA-575
    - 1 w/v% LAP
    - 1X TAEM
    - 500nM 5Acry_3Cy3_polyT10
4) Add 800 µL of 1X TAEM (wash solution) into each of three vials.
5) Add 800 µL of 1M NaCl (lift-off solution) into the fifth and final vial.
6) Screw in each modified cap to its corresponding vial to connect all five vials to the five ink/wash tubes that are connected to the flow controller (**SI Section 3**).
7) Set the pressure for each vial on the low-volume flow controller to 3 PSI.
8) To prime the first ink tube, run the ink solution through the first ink tube by opening the first valve on the low-volume flow controller.
9) Using a 0.75mm biopsy punch to assist, insert the ink tube primed in step 8 into the first inlet of the microfluidic chamber.
10) Open the valve, to flow the ink solution through the microfluidic chamber until the ink solution reaches the outlet of the microfluidic chamber, then stop the flow by closing the valve.
11) Repeat steps 7-9 for the solutions in the other four vials, performing these steps for the vial containing the wash solution last.
12) Place the microfluidic flow chamber on the XY Stage, place the vials at a higher height than the microfluidic flow chamber (so that gravity assists rather than impedes flow), and place the end of a

waste tube on the rim of a waste receptacle (i.e., a 15 mL Falcon tube). The waste receptacle should be at the same height as the vials.

13) Focus on the surface of the microfluidic flow chamber with the 10X objective.
14) Clamp the waste tubing and all ink tubes except the wash tube.
15) Increase pressure on the flow controller for the wash vial to 5 PSI. Then open the wash valve and leave the wash valve open until all remaining air is removed from the microfluidic flow chamber. To verify all air is removed, look through the microfluidic flow chamber using the microscope.
16) Return the pressure on the flow controller to 3 PSI and stop the flow of the wash solution by closing the valve.
17) Unclamp the waste, ink, and solution tubes.
18) Move field of view of the camera to the patterning channel within the microfluidic flow chamber and refocus if necessary. Then adjust microscope focus to 50% of a full rotation of the focus knob below the channel focal plane for patterning.
19) Run the MAPDH-LC script.
    - MAPDH-LC script.
        - https://github.com/MishaRubanov/MAPDH/tree/main/Supplemental%20protocols%20and%20data
    - The MAPDH-LC script runs the following tasks:
        - Flow ink solution into the lift-off flow chamber.
        - Pattern 20 hydrogels (200 µm squares) within specified locations.
        - Image hydrogels at specified locations.
        - Flow in 1x TAEM solution to remove residual ink solution.
        - Image hydrogels at specified locations.
        - Flow in 1M NaCl solution for 1 minute to initiate dissolution of the PAA sacrificial layer.
        - Image hydrogels at specified locations.
        - Wait 1 hour.
20) Replace the outlet Falcon tube connected to the waste receptacle with a 1.7 mL microcentrifuge tube for collection.
21) Flow in 1X TAEM solution to convectively remove detached hydrogels.
22) Image hydrogel patterning locations to determine percentage of hydrogels that successfully detached.
23) Centrifuge the 1.7 mL microcentrifuge tube for 10-20 seconds at 6000 RPM.
24) Pipette 20 mL from the bottom of the centrifuged solution into a well of a 96-well plate.
25) Image collected hydrogels within the 96-well plate.

We used the lift-off flow chamber (**SI Section 20**) for patterning, lift-off, and collection of DNA-functionalized hydrogels. After turning on all the necessary equipment and software (step 1) (**SI Section 30**), we set up the lift-off flow chamber by inserting the waste tubing first (step 2). The waste tubing had a larger diameter compared to the ink/wash tubing (0.02"x0.06") to ensure low resistance of the outlet so that fluid prefers to flow through the waste tubing rather than through the ink/wash tubing. 24 inches of tubing was used to ensure that there was enough tubing to reach the waste receptacle.

We made one ink solution containing 500nM of strand 5Acry_3Cy3_polyT10. This solution was then aliquoted to a vial (step 3) that are then connected to the low-volume flow controller. We then added solutions containing 1x TAEM and 1 M NaCl to the other four vials (steps 4-5). Each vial had a modified lid (step 6) with one hole through which a hollow point needle connected to an air source (for pressure) was inserted and one hole through which the low volume ink/wash tubing (0.01"x0.03") fed through to the bottom of the vial for flow of solution from the bottom of the vial to the microfluidic flow chamber (**SI**

Section 3). We used small-diameter ink/wash tubing to minimize the volume that was retained in the tubing and to increase the resistance to flow, which helped prevent backflow from one inlet into another.

For each inlet, we set the flow controller pressures to 3 PSI (step 7)—a value chosen to give moderate flow through the microfluidic flow chamber. Before connecting the tube to the microfluidic flow chamber , we primed the tube by opening the valve on the flow controller and letting the tubing fill with liquid until a bead of liquid was at the end of the tube (step 8). The tube for the wash solution was inserted last (*i.e.,* after all of the ink solutions) to ensure no ink remained within the microfluidic flow chamber during the rest of the setup process (step 10). The ink solutions and a waste receptacle were placed at the same level above the stage of the microscope to balance the hydrostatic pressure between the fluid source and the outlet of the tubing, and to drive flow down into the microfluidic flow chamber. After placing the microfluidic chamber on the stage (step 12), we used the 10X objective to focus on the flow chamber channel where we wanted to pattern (step 13) and then checked for air in the whole microfluidic flow chamber by moving the stage around. If air was visible, then we degassed the microfluidic flow chamber. This was done by clamping the waste tube and all the ink tubes except the wash solution tube (step 14), which meant we could increase the pressure inside of the microfluidic flow chamber, since fluid could not move out of the outlet or the other inlets, causing the air to compress and diffuse through the PDMS. After clamping everything except the wash solution tube, we opened the valve to the wash, increased the pressure to the wash vial on the flow controller to 5 PSI, and waited for the air in the microfluidic flow chamber to diffuse out through the PDMS. This usually took a few minutes (steps 15-17).

To prepare for patterning, we focused on the channel of the microfluidic flow chamber through the camera and then moved 50% of a full rotation of the focus knob (*i.e.*, the location of the objective relative to the patterning chamber) below the PDMS: PDMS interface as focused on by the camera (step 18). The plane that the camera is focused on was lowered to allow the UV light coming from the digital micromirror microfluidic flow chamber to be in focus, which allowed for hydrogels patterned at that focal plane to have well-defined features (**SI Figure 3A**). This is due to the different locations for the DMD and camera on the microscope.

We used 1 M of NaCl solution to dissolve the sacrificial layer by converting the PAA into its water-soluble form via Na+ ion exchange (step 19). During the 1 hour wait step after flowing in 1M NaCl, we replaced the waste receptacle with a 1.7 mL microcentrifuge tube so that for the next wash step, the hydrogels are collected in the 1.7 mL microcentrifuge tube rather than the waste receptacle. We used a higher pressure to convectively remove the hydrogels from the chamber (5 PSI) (step 20). After an hour of waiting for the PAA layer to dissolve, we flow in 1 mL of 1x TAEM solution to convectively remove detached hydrogels and collect them in the microcentrifuge tube (step 21). We imaged the original patterning locations for all hydrogels to determine the percentage of detached hydrogels (step 22).

To concentrate the hydrogels, we centrifuge the 1.7 mL microcentrifuge tube at a spin rate of 6000 RPM, allowing collection of the settled hydrogels from the bottom of the tube (step 23). We can then aliquot 20 mL from the bottom of the collected solution into a well within a 96-well plate for further (step 24). On average, each 20 mL aliquot contained 3-5 hydrogels.

22. Analysis of protocol for fabrication of single-domain hydrogels using MAPDH-LC

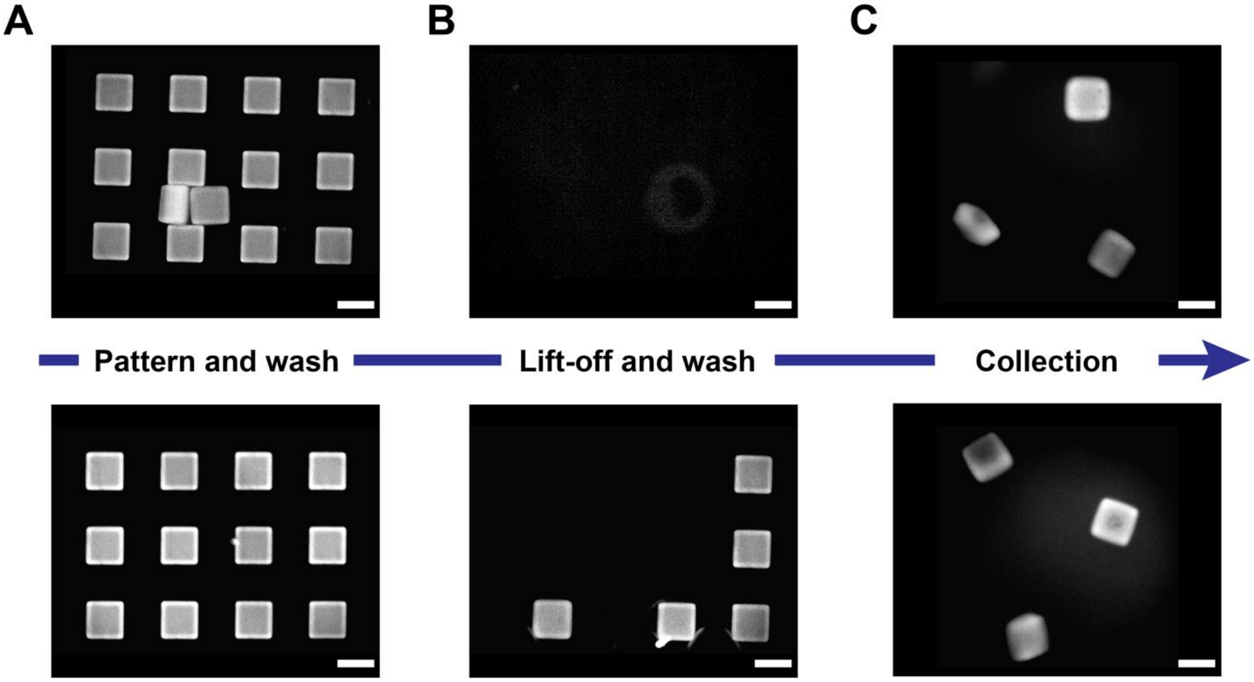

Figure 18. Demonstration of hydrogel production. A) Photopatterning and washing. B) Lift-off and washing. C) Collection in a well of a 96-well plate. Top and bottom micrographs are two different locations from the same round of single-domain hydrogel patterning. Scale bars are 200 µm.

We patterned a total of 25 single-domain hydrogels, and then washed the lift-off flow chamber with 1X TAEM. We postulated that not all the hydrogels were robustly anchored to the sacrificial layer after photopatterning. As shown in **Figure S18A**, after the washing process a few hydrogels appeared to have detached from their patterned location. After dissolution of the sacrificial layer and washing the chamber with 1X TAEM, most of the hydrogels were removed (**Figure S18B**).

Of the 25 hydrogels that we patterned; 13 free hydrogels were collected within the 96 well plate (**Figure S18C**). We hypothesize that we achieved lower yields in collected hydrogels due to three reasons: some hydrogels remained anchored to the lift-off flow chamber despite the dissolution of the sacrificial layer using 1 M NaCl, some hydrogels detached prematurely and therefore were not collected in the collection tube, and some hydrogels may have been lost during transfer due to sticking to the pipette tip walls or the 1.7 mL microcentrifuge tube.

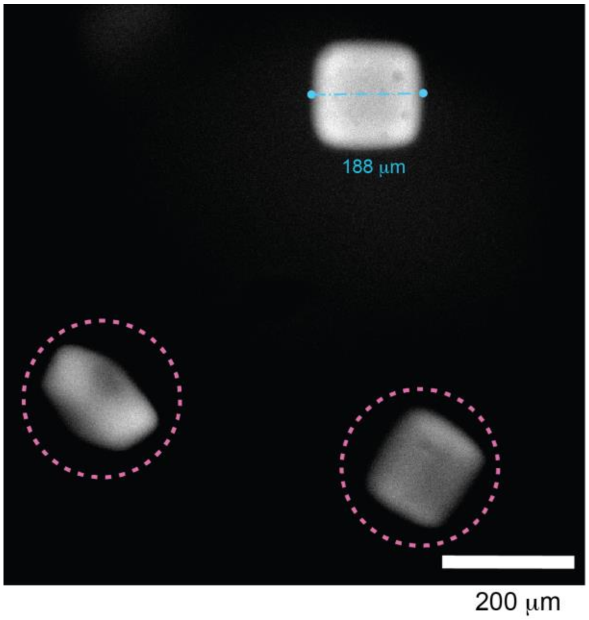

Figure 19. Representative micrograph of MAPDH single-domain hydrogels in a well of a 96-well plate. Hydrogels on their sides and/or not flat on the glass surface are enclosed by dashed, pink circles. Only hydrogels that lay flat on the bottom surface of the plate were further analyzed (top hydrogel).

After collection of the hydrogels in a well of a 96-well plate, we observed that some of the collected hydrogels were flush with the well surface, while others were on their sides (**SI Figure 19**). For consistency in measurement, we analyzed only the collected hydrogels that were flush with the well surface.

We wanted to see whether the collected hydrogels were similar in size compared to the hydrogels that had just been patterned and washed. We compared lengths of seven randomly selected hydrogels within the lift-off flow chamber (before lift-off and collection) to seven collected hydrogels. As shown in Figure 4D, there were no significant differences in size between the hydrogel lengths after patterning and washing (195±9 µm) and hydrogel lengths after collection (189±14 µm), suggesting that the hydrogels undergo no significant change in size after the lift-off, washing, and collection processes.

23. Protocol for multi-domain hydrogel fabrication, liftoff, and collection

The list of steps to run the protocol are shown below, followed by a detailed explanation of the protocol.

1) Turn on all MAPDH hardware, connect to Micromanager and Pycromanager (**SI Section 30**).
2) Insert the waste tube, 24-inch length of Tygon tubing (0.02"x0.06"), into the outlet of the 5-inlet 100µm empty (air-filled) lift-off flow chamber.
3) Make three 200µL ink solutions in three vials, each with the following composition:
    - 10 v/v% PEGDA-575
    - 1 w/v% LAP
    - 1X TAEM
    - Acrydite and fluorophore modified DNA.
        - Ink 1 (green domain): 500nM 5Acry_3ATTO488_polyT10
        - Ink 2 (red domain): 500nM 5Acry_3Cy3_polyT10
        - Ink 3 (blue domain): 500nM 5Acry_3TYE665_polyT10

4) Add 800 µL of 1X TAEM (wash solution) into a fourth vial.
5) Add 800 µL of 1M NaCl (lift-off solution) into the fifth and final vial.
6) Screw in each modified cap to its corresponding vial to connect all five vials to the five ink/wash tubes that are connected to the flow controller (**SI Section 3**).
7) Set the pressure for each vial on the low-volume flow controller to 3 PSI.
8) To prime the first ink tube, run the ink solution through the first ink tube by opening the first valve on the low-volume flow controller.
9) Using a 0.75mm biopsy punch to assist, insert the ink tube primed in step 8 into the first inlet of the microfluidic chamber.
10) Open the valve, to flow the ink solution through the microfluidic chamber until the ink solution reaches the outlet of the microfluidic chamber, then stop the flow by closing the valve.
11) Repeat steps 7-9 for the solutions in the other four vials, performing these steps for the vial containing the wash solution last.
12) Place the microfluidic flow chamber on the XY Stage, place the vials at a higher height than the microfluidic flow chamber (so that gravity assists rather than impedes flow), and place the end of a waste tube on the rim of a waste receptacle (i.e., a 15 mL Falcon tube). The waste receptacle should be at the same height as the vials.
13) Focus on the surface of the microfluidic flow chamber with the 10X objective.
14) Clamp the waste tubing and all ink tubes except the wash tube.
15) Increase pressure on the flow controller for the wash vial to 5 PSI. Then open the wash valve and leave the wash valve open until all remaining air is removed from the microfluidic flow chamber. To verify all air is removed, look through the microfluidic flow chamber using the microscope.
16) Return the pressure on the flow controller to 3 PSI and stop the flow of the wash solution by closing the valve.
17) Unclamp the waste, ink, and solution tubes.
18) Move field of view of the camera to the patterning channel within the microfluidic flow chamber and refocus if necessary. Then adjust microscope focus to 50% of a full rotation of the focus knob below the channel focal plane for patterning.
19) Run the MAPDH-LC script.
    - MAPDH-LC script
        - https://github.com/MishaRubanov/MAPDH/tree/main/Supplemental%20protocols%20and%20data
    - The MAPDH-LC script runs the following tasks:
        - Flow ink 1 (green domain) solution into the lift-off flow chamber.
        - Pattern 20 green-domain hydrogels (200 µm squares) within specified locations.
        - Flow in 1x TAEM solution to remove residual ink solution.
        - Image hydrogels at specified locations.
        - Flow ink 2 (red domain) solution into the lift-off flow chamber.
        - Pattern 20 hydrogels (200 µm squares) to the left of each green-domain hydrogel such that there is a 10 µm overlap.
        - Flow in 1x TAEM solution to remove residual ink solution.
        - Image hydrogels at specified locations.
        - Flow ink 3 (blue domain) solution into the lift-off flow chamber.
        - Pattern 20 hydrogels (200 µm squares) to the right of each green-domain hydrogel such that there is a 10 µm overlap.
        - Flow in 1x TAEM solution to remove residual ink solution.
        - Image hydrogels at specified locations.

- Flow in 1x TAEM solution to remove residual ink solution.
- Image hydrogels at specified locations.
- Flow in 1M NaCl solution for 1 minute to initiate dissolution of the PAA sacrificial layer.
- Image hydrogels at specified locations.
- Wait 1 hour.

20) Replace the outlet Falcon tube connected to the waste receptacle with a 1.7 mL microcentrifuge tube for collection.
21) Flow in 1X TAEM solution to convectively remove detached hydrogels.
22) Image hydrogel patterning locations to determine percentage of hydrogels that successfully detached.
23) Centrifuge the 1.7 mL microcentrifuge tube for 10-20 seconds at 6000 RPM.
24) Pipette 20 mL from the bottom of the centrifuged solution into a well of a 96-well plate.
25) Image collected hydrogels within the 96-well plate.

We used the lift-off flow chambers (**SI Section 20**) for patterning, lift-off, and collection of DNA-functionalized hydrogels. After turning on all the necessary equipment and software (step 1) (**SI Section 30**), we set up the lift-off flow chamber by inserting the waste tubing first (step 2). The waste tubing had a larger diameter compared to the ink/wash tubing (0.02”x0.06”) to ensure low resistance of the outlet so that fluid prefers to flow through the waste tubing rather than through the ink/wash tubing. 24 inches of tubing was used to ensure that there was enough tubing to reach the waste receptacle.

We made three ink solutions of 200µL each using 3 differently modified strands of DNA each with an acrydite group, a sequence, and a different fluorophore. The first three of these solutions were made using 500nM of one of the following strands: 5Acry_3Cy3_polyT10, 5Acry_3ATTO488_polyT10, or 5Acry_3TYE665_polyT10. The solutions were then aliquoted to a vial (step 3) that are then connected to the low-volume flow controller. We then added solutions containing 1x TAEM and 1 M NaCl to the other two vials (steps 4-5). Each vial had a modified lid (step 6) with one hole through which a hollow point needle connected to an air source (for pressure) was inserted and one hole through which the low volume ink/wash tubing (0.01”x0.03”) fed through to the bottom of the vial for flow of solution from the bottom of the vial to the microfluidic flow chamber (**SI Section 3**). We used small-diameter ink/wash tubing to minimize the volume that was retained in the tubing and to increase the resistance to flow, which helped prevent backflow from one inlet into another.

For each inlet, we set the flow controller pressures to 3 PSI (step 7)—a value chosen to give moderate flow through the microfluidic flow chamber. Before connecting the tube to the microfluidic flow chamber , we primed the tube by opening the valve on the flow controller and letting the tubing fill with liquid until a bead of liquid was at the end of the tube (step 8). The tube for the wash solution was inserted last (*i.e.*, after all of the ink solutions) to ensure no ink remained within the microfluidic flow chamber during the rest of the setup process (step 10). The ink solutions and a waste receptacle were placed at the same level above the stage of the microscope to balance the hydrostatic pressure between the fluid source and the outlet of the tubing, and to drive flow down into the microfluidic flow chamber. After placing the microfluidic chamber on the stage (step 12), we used the 10X objective to focus on the flow chamber channel where we wanted to pattern (step 13) and then checked for air in the whole microfluidic flow chamber by moving the stage around. If air was visible, then we degassed the microfluidic flow chamber. This was done by clamping the waste tube and all the ink tubes except the wash solution tube (step 14), which meant we could increase the pressure inside of the microfluidic flow chamber, since fluid could not move out of the outlet or the other inlets, causing the air to compress and diffuse through the PDMS. After clamping everything except the wash solution tube, we opened the valve to the wash, increased the pressure to the wash vial on

the flow controller to 5 PSI, and waited for the air in the microfluidic flow chamber to diffuse out through the PDMS. This usually took a few minutes (steps 15-17).

To prepare for patterning, we focused on the channel of the microfluidic flow chamber through the camera and then moved 50% of a full rotation of the focus knob (*i.e.*, the location of the objective relative to the patterning chamber) below the PDMS: PDMS interface as focused on by the camera (step 18). The plane that the camera is focused on was lowered to allow the UV light coming from the digital micromirror microfluidic flow chamber to be in focus, which allowed for hydrogels patterned at that focal plane to have well-defined features (**SI Figure 3A**). This is due to the different locations for the DMD and camera on the microscope.

The green domain was first patterned, and the locations for the 20 green domain hydrogels were used to determine the locations to pattern hydrogels within the red and blue domains (step 19). The red and blue domains were patterned with a 10-micron overlap with the green domain to ensure that the different domains remain connected to one another after lift-off and collection. We used 1 M of NaCl solution to dissolve the sacrificial layer by converting the PAA into its water-soluble form via Na+ ion exchange (step 19). During the 1 hour wait step after flowing in 1M NaCl, we replaced the waste receptacle with a 1.7 mL microcentrifuge tube so that for the next wash step, the hydrogels are collected in the 1.7 mL microcentrifuge tube rather than the waste receptacle (step 20). After an hour of waiting for the PAA layer to dissolve, we flow in 1 mL of 1x TAEM solution to convectively remove detached hydrogels and collect them in the microcentrifuge tube (step 21). We imaged the original patterning locations for all hydrogels between wash steps to determine the percentage of detached hydrogels at each round of patterning (step 22).

To concentrate the hydrogels, we centrifuge the 1.7 mL microcentrifuge tube at a spin rate of 6000 RPM, allowing collection of the settled hydrogels from the bottom of the tube (step 23). We can then aliquot 20 mL from the bottom of the collected solution into a well within a 96-well plate for further (step 24). On average, each 20 mL aliquot contained 3-5 hydrogels.

24. Analysis for multi-domain MAPDH-LC

For fabrication of multi-domain hydrogel architectures using MAPDH-LC, 25 hydrogels were first patterned conjugated with 5Acry_3ATTO488_polyT10 (colored green in **Figure S20**), followed by 25 hydrogels conjugated with 5Acry_3Cy3_polyT10 (colored red in **Figure S20**), followed by 25 hydrogels conjugated with 5Acry_3TYE665_polyT10 (colored blue in **Figure S20**). During the washing and patterning steps, we observed 13 of the 25 hydrogels remained adhered to the sacrificial layer surface. Some of the unadhered hydrogels (**Figure S20C**), were unaligned, or completely detached, during fabrication. Of the aligned hydrogels (**Figure S20A**), two were successfully collected (**Figure 20B**).

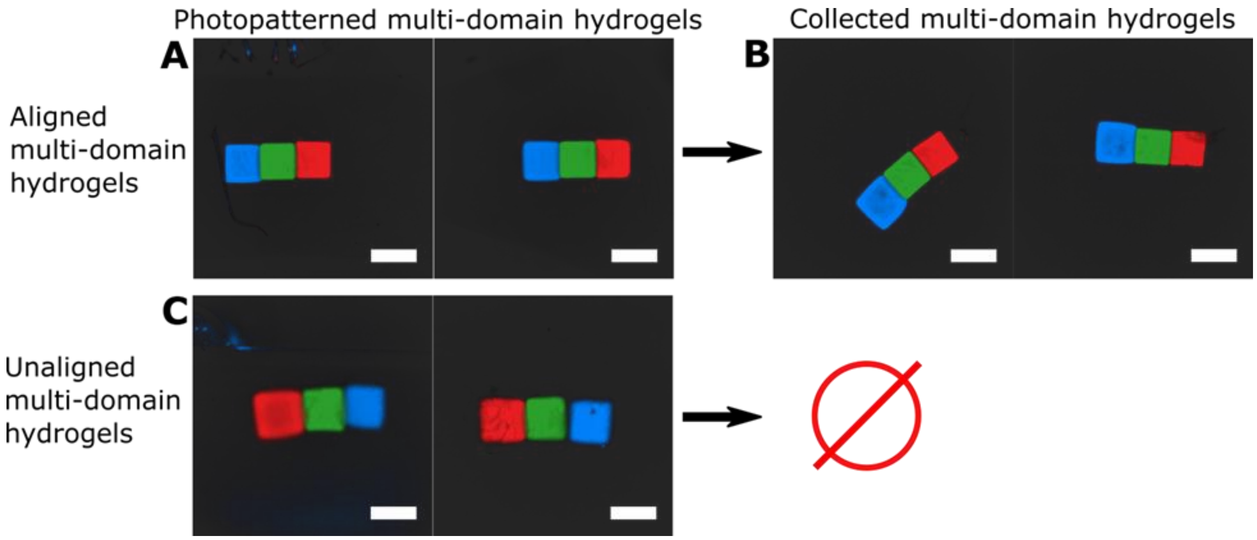

Figure 20. Examples multi-domain hydrogels fabricated using MAPDH-LC. Out of the 13 aligned hydrogels 2 were successfully collected. A) Hydrogels in the microfluidic device. B) Hydrogels collected and placed in a well of a 96-well plate. C) Unaligned multi-domain gels due to poor adhesion between the hydrogel and the sacrificial layer. Scale bars are 200 µm.

25. Protocol for single-domain, DNA-crosslinked hydrogel fabrication, lift-off, and collection

The list of steps to run the protocol are shown below, followed by a detailed explanation of the protocol.

1) Turn on all MAPDH hardware, connect to Micromanager and Pycromanager (**SI Section 30**).
2) Insert the waste tube, 24-inch length of Tygon tubing (0.02”x0.06”), into the outlet of the 5-inlet 100µm empty (air-filled) lift-off flow chamber.
3) Prepare the DNA crosslinks.
    - Make a system 1 DNA crosslink mixture by mixing S1 and S1’ in a 200 mL PCR tube with 1X TAEM buffer at a concentration of 3 mM per strand.
    - Heat the DNA crosslink mixture to 90 °C, then slowly cool down to 20 °C using a thermocycler at a rate of 1 °C min^{-1} to anneal the crosslinks.
4) Make a 100µL ink solution in one vial with the following composition:
    - 10 v/v% PEGDA-10K
    - 1 w/v% LAP
    - 1X TAEM
    - 500nM 5Acry_3Cy3_polyT10
    - 1.15 mM system 1 DNA crosslink mixture (annealed S1:S1’)
5) After thoroughly pipette mixing the ink solution, degas the ink solution under vacuum for at least 10 mins.
6) Add 800 µL of 1X TAEM (wash solution) into each of three vials.
7) Add 800 µL of 1M NaCl (lift-off solution) into the fifth and final vial.
8) Screw in each modified cap to its corresponding vial to connect all five vials to the five ink/wash tubes that are connected to the flow controller (**SI Section 3**).
9) Set the pressure for each vial on the low-volume flow controller to 3 PSI.
10) To prime the first ink tube, run the ink solution through the first ink tube by opening the first valve on the low-volume flow controller.
11) Using a 0.75mm biopsy punch to assist, insert the ink tube primed in step 10 into the first inlet of the microfluidic chamber.

12) Open the valve, to flow the ink solution through the microfluidic chamber until the ink solution reaches the outlet of the microfluidic chamber, then stop the flow by closing the valve.
13) Repeat steps 7-9 for the solutions in the other four vials, performing these steps for the vial containing the wash solution last.
14) Place the microfluidic flow chamber on the XY Stage, place the vials at a higher height than the microfluidic flow chamber (so that gravity assists rather than impedes flow), and place the end of a waste tube on the rim of a waste receptacle (i.e., a 15 mL Falcon tube). The waste receptacle should be at the same height as the vials.
15) Focus on the surface of the microfluidic flow chamber with the 10X objective.
16) Clamp the waste tubing and all ink tubes except the wash tube.
17) Increase pressure on the flow controller for the wash vial to 5 PSI. Then open the wash valve and leave the wash valve open until all remaining air is removed from the microfluidic flow chamber. To verify all air is removed, look through the microfluidic flow chamber using the microscope.
18) Return the pressure on the flow controller to 3 PSI and stop the flow of the wash solution by closing the valve.
19) Unclamp the waste, ink, and solution tubes.
20) Move field of view of the camera to the patterning channel within the microfluidic flow chamber and refocus if necessary. Then adjust microscope focus to 50% of a full rotation of the focus knob below the channel focal plane for patterning.
21) Run the MAPDH-LC script.
    - MAPDH-LC script
        - https://github.com/MishaRubanov/MAPDH/tree/main/Supplemental%20protocols%20and%20data
    - The MAPDH-LC script runs the following tasks:
        - Flow ink solution into the lift-off flow chamber.
        - Pattern 20 hydrogels (200 µm squares) within specified locations.
        - Image hydrogels at specified locations.
        - Flow in 1x TAEM solution to remove residual ink solution.
        - Image hydrogels at specified locations.
        - Flow in 1M NaCl solution for 1 minute to initiate dissolution of the PAA sacrificial layer.
        - Image hydrogels at specified locations.
        - Wait 1 hour.
22) Replace the outlet Falcon tube connected to the waste receptacle with a 1.7 mL microcentrifuge tube for collection.
23) Flow in 1X TAEM solution to convectively remove detached hydrogels.
24) Image hydrogel patterning locations to determine percentage of hydrogels that successfully detached.
25) Centrifuge the 1.7 mL microcentrifuge tube for 10-20 seconds at 6000 RPM.
26) Pipette 20 mL from the bottom of the centrifuged solution into a well of a 96-well plate.
27) Image collected hydrogels within the 96-well plate.

We used the lift-off flow chamber (**SI Section 20**) for patterning, lift-off, and collection of DNA-functionalized, DNA-crosslinked hydrogels. After turning on all the necessary equipment and software (step 1) (**SI Section 30**), we set up the lift-off flow chamber by inserting the waste tubing first (step 2). The waste tubing had a larger diameter compared to the ink/wash tubing (0.02”x0.06”) to ensure low resistance of the outlet so that fluid prefers to flow through the waste tubing rather than through the ink/wash tubing. 24 inches of tubing was used to ensure that there was enough tubing to reach the waste receptacle.

The DNA crosslink sequences were those reported previously by Cangialosi et al. as System 1 strands [5] (step 3). System 1 DNA crosslink strands (S1 and S1') (sequences in **SI Section 34**) are purchased lyophilized from Integrated DNA Technologies (IDT) without additional purification. We diluted the DNA crosslink to 3 mM per strand, then followed an annealing protocol where the strands are mixed and heated to 90 °C to completely denature the strands, and slowly cooled to 20°C so that the two DNA strands can hybridize correctly (step 3). We used PEGDA 10K M_n in the ink formulation (step 4) due to higher-degree swelling compared to PEGDA 575 M_n [7].

We made one ink solution containing 500nM of strand 5Acry_3Cy3_polyT10 and 1.15 mM S1:S1'. This solution was then aliquoted to a vial (step 4) that are then connected to the low-volume flow controller. We then added solutions containing 1x TAEM and 1 M NaCl to the other four vials (steps 6-7). Each vial had a modified lid (step 8) with one hole through which a hollow point needle connected to an air source (for pressure) was inserted and one hole through which the low volume ink/wash tubing (0.01"x0.03") fed through to the bottom of the vial for flow of solution from the bottom of the vial to the microfluidic flow chamber (**SI Section 3**). We used small-diameter ink/wash tubing to minimize the volume that was retained in the tubing and to increase the resistance to flow, which helped prevent backflow from one inlet into another.

For each inlet, we set the flow controller pressures to 3 PSI (step 9)—a value chosen to give moderate flow through the microfluidic flow chamber. Before connecting the tube to the microfluidic flow chamber , we primed the tube by opening the valve on the flow controller and letting the tubing fill with liquid until a bead of liquid was at the end of the tube (step 10). The tube for the wash solution was inserted last (*i.e.*, after all of the ink solutions) to ensure no ink remained within the microfluidic flow chamber during the rest of the setup process (step 11). The ink solutions and a waste receptacle were placed at the same level above the stage of the microscope to balance the hydrostatic pressure between the fluid source and the outlet of the tubing, and to drive flow down into the microfluidic flow chamber. After placing the microfluidic chamber on the stage (step 14), we used the 10X objective to focus on the flow chamber channel where we wanted to pattern (step 15) and then checked for air in the whole microfluidic flow chamber by moving the stage around. If air was visible, then we degassed the microfluidic flow chamber. This was done by clamping the waste tube and all the ink tubes except the wash solution tube (step 16), which meant we could increase the pressure inside of the microfluidic flow chamber, since fluid could not move out of the outlet or the other inlets, causing the air to compress and diffuse through the PDMS. After clamping everything except the wash solution tube, we opened the valve to the wash, increased the pressure to the wash vial on the flow controller to 5 PSI, and waited for the air in the microfluidic flow chamber to diffuse out through the PDMS. This usually took a few minutes (steps 17-19).

To prepare for patterning, we focused on the channel of the microfluidic flow chamber through the camera and then moved 50% of a full rotation of the focus knob (*i.e.*, the location of the objective relative to the patterning chamber) below the PDMS: PDMS interface as focused on by the camera (step 20). The plane that the camera is focused on was lowered to allow the UV light coming from the digital micromirror microfluidic flow chamber to be in focus, which allowed for hydrogels patterned at that focal plane to have well-defined features (**SI Figure 3A**). This is due to the different locations for the DMD and camera on the microscope.

We used 1 M of NaCl solution to dissolve the sacrificial layer by converting the PAA into its water-soluble form via Na+ ion exchange (step 21). During the 1 hour wait step after flowing in 1M NaCl, we replaced the waste receptacle with a 1.7 mL microcentrifuge tube so that for the next wash step, the hydrogels are collected in the 1.7 mL microcentrifuge tube rather than the waste receptacle (step 20). After an hour of waiting for the PAA layer to dissolve, we flow in 1 mL of 1x TAEM solution to convectively remove

detached hydrogels and collect them in the microcentrifuge tube (step 23). We imaged the original patterning locations for all hydrogels to determine the percentage of detached hydrogels (step 24).

To concentrate the hydrogels, we centrifuge the 1.7 mL microcentrifuge tube at a spin rate of 6000 RPM, allowing collection of the settled hydrogels from the bottom of the tube (step 25). We can then aliquot 20 mL from the bottom of the collected solution into a well within a 96-well plate for further (step 26). On average, each 20 mL aliquot contained 3-5 hydrogels.

26. Protocol for swelling single-domain hydrogels

1. Dilute System 1 hairpins (S1_H1 and S1_H2) to a concentration 100 mM in 1x TAEM buffer for each strand.
2. Heat the S1_H1 and S1_H2 DNA solutions to 95 °C for 15 mins using an Eppendorf PCR instrument.
3. Quickly place the heated strands in an ice bath for 5 mins.
4. Pipette each hairpin into the well that contains the single-domain MAPDH hydrogels for a final concentration of 20 mM of each hairpin.
5. Run time-lapse fluorescence imaging with images taken at 30-minute intervals to measure change in hydrogel length.

System 1 hairpins (S1_H1, S1_H2) were purchased in a lyophilized form from IDT without additional purification. Following an established protocol to swell the DNA-functionalized hydrogels [5], system 1 DNA swelling signals were first diluted to 100 mM and then heated to 95 °C to completely denature the single-stranded DNA and prevent undesired secondary structures (steps 1-2). We then quickly placed the heated strands in an ice bath so that the strands form a secondary hairpin structure (steps 3). After formation of the hairpins, we pipetted 20 mL of S1_H1 and S1_H2 DNA solutions into the well that contains the single-domain MAPDH hydrogels (step 4). We then added 40 mL of 1x TAEM buffer solution for a total volume of 100 mL and a final DNA swelling signal concentration of 20 mM. We then ran time-lapse fluorescence imaging of the swelling hydrogels (step 5).

27. Automated measurement of hydrogel lengths

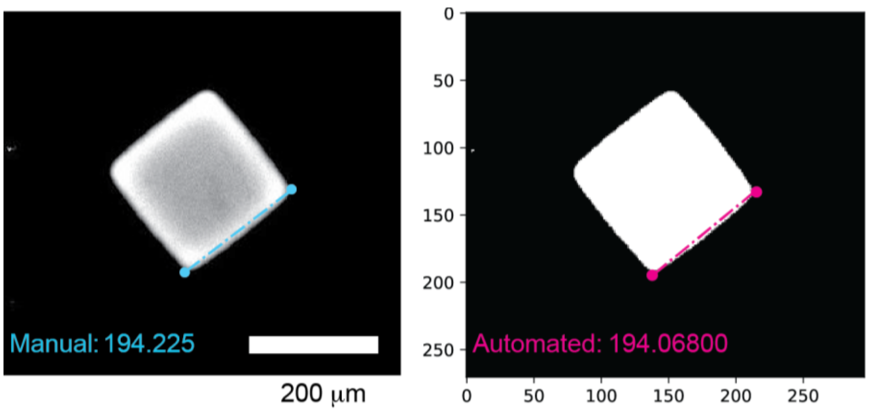

Figure 21. Demonstration of measuring hydrogel lengths and comparison of (left) manual and (right) automated measurements. Herein, to automatically measure the hydrogel length we binarized the micrograph by a threshold calculated via Otsu's method. (Reference to MAPDH script).

In **Figure 4B** we manually measured the side lengths of each hydrogel via ImageJ. by measuring the corner-to-corner distances. However, due to the large quantity of hydrogel micrographs that we wanted to analyze during the swelling experiments (approximately 60 micrographs per gel), we developed an automated script for measuring hydrogel length (**SI Figure 19**). First, we binarized the micrograph using a threshold determined via Otsu's thresholding method [7], then used the scikit-image Harris corner detector package to automatically identify corners of the hydrogel. After identifying the corners of each hydrogel, we determined the end-to-end distance of the corners. Code for this analysis is found at https://github.com/MishaRubanov/MAPDH.

28. Analysis for single-domain hydrogel swelling

In accordance with methods described in Cangialosi et al.[5], free-floating DNA-functionalized hydrogels undergo swelling when DNA swelling signals, in the form of hairpins, are pipetted into the well with the single- or multi- domain hydrogels to initiate the hairpin chain reaction. Using the script for measuring hydrogel lengths automatically (SI Section 27), we calculated the lengths of each hydrogel over 60 hours at 30-minute intervals. Hydrogel swelling was calculated by comparing the end-to-end length of the hydrogel in each time-lapse micrograph to the initial end-to-end length of the hydrogel before addition of the DNA swelling signal. To ensure that the automated length measurement of the hydrogels was accurate, the automated measurement was compared to the manually measured length for the same hydrogel at three different time points, at t = 0hr, t = 30hrs, and t = 60hrs (**SI Figure 22**). The measurements were different by at most 6.57 microns. Since the measurements varied by at most 4%, we chose to use the automated script to measure all hydrogel lengths.

Data for the free-floating hydrogel swelling were plotted as changes in length divided by the initial length ($\Delta L/L_0$) over time and smoothened by calculating and applying a moving average over eight values using the numpy.convolve module. The analysis was repeated over 11 replicate hydrogels that were collected and swelled in the same imaging plate. *Shi et al*. reported that DNA-functionalized PEGDA with 10K number average molecular weight (M_n) hydrogels should achieve approximately 0.4 ($\Delta L/L_0$) at 60 hours from initiation of swelling [8]. The single-domain DNA-functionalized, PEGDA-10k hydrogels exhibited swelling of approximately 0.51 ($\Delta L/L_0$) at 60 hours (**Figure 5B**), suggesting compatibility between the

previously reported DNA-functionalized PEGDA-10k M_n hydrogel fabrication method and the MAPDH fabrication method for DNA-directed swelling hydrogels.

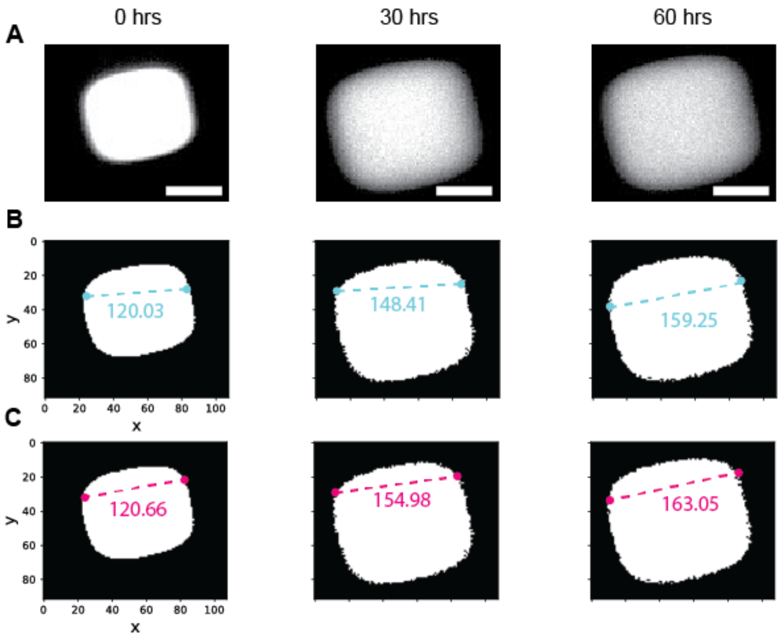

Figure 22. Analysis of single hydrogel swelling. A) Original time-lapse micrograph of single-domain MAPDH hydrogel swelling. B) Automated micrograph analysis of single-domain MAPDH hydrogel swelling and measurement of length changes. C) Manual measurements of single-domain MAPDH hydrogel swelling and length change.

29. Protocol for multi-domain, DNA-crosslinked hydrogel fabrication, lift-off, and collection

The list of steps to run the protocol are shown below, followed by a detailed explanation of the protocol.

1) Turn on all MAPDH hardware, connect to Micromanager and Pycromanager (**SI Section 30**).
2) Insert the waste tube, 24-inch length of Tygon tubing (0.02”x0.06”), into the outlet of the 5-inlet 100µm empty (air-filled) lift-off flow chamber.
3) Prepare the DNA crosslinks.
    - Make a system 1 DNA crosslink mixture by mixing S1 and S1’ in a 200 mL PCR tube with 1X TAEM buffer at a concentration of 3 mM per strand.
    - Make a system 2 DNA crosslink mixture by mixing S2 and S2’ in a 200 mL PCR tube with 1X TAEM buffer at a concentration of 3 mM per strand.
    - Heat the DNA crosslink mixture to 90 °C, then slowly cool down to 20 °C using a thermocycler at a rate of 1 °C min^{-1} to anneal the crosslinks.
4) Make three 200µL ink solutions in three vials, each with the following composition:
    - 10 v/v% PEGDA-10K
    - 1 w/v% LAP
    - 1X TAEM
    - Modified DNA.
        - Ink 1 (green domain): 500nM 5Acry_3ATTO488_polyT10
        - Ink 2 (red domain): 500nM 5Acry_3Cy3_polyT10, 1.15 mM system 1 DNA crosslink mixture (annealed S1:S1’)

        - Ink 3 (blue domain): 500nM 5Acry_3TYE665_polyT10, 1.15 mM system 2 DNA crosslink mixture (annealed S2:S2')

5) After thoroughly pipette mixing the ink solution, degas the ink solution under vacuum for at least 10 mins.
6) Add 800 µL of 1X TAEM (wash solution) into a fourth vial.
7) Add 800 µL of 1M NaCl (lift-off solution) into the fifth and final vial.
8) Screw in each modified cap to its corresponding vial to connect all five vials to the five ink/wash tubes that are connected to the flow controller (**SI Section 3**).
9) Set the pressure for each vial on the low-volume flow controller to 3 PSI.
10) To prime the first ink tube, run the ink solution through the first ink tube by opening the first valve on the low-volume flow controller.
11) Using a 0.75mm biopsy punch to assist, insert the ink tube primed in step 10 into the first inlet of the microfluidic chamber.
12) Open the valve, to flow the ink solution through the microfluidic chamber until the ink solution reaches the outlet of the microfluidic chamber, then stop the flow by closing the valve.
13) Repeat steps 7-9 for the solutions in the other four vials, performing these steps for the vial containing the wash solution last.
14) Place the microfluidic flow chamber on the XY Stage, place the vials at a higher height than the microfluidic flow chamber (so that gravity assists rather than impedes flow), and place the end of a waste tube on the rim of a waste receptacle (i.e., a 15 mL Falcon tube). The waste receptacle should be at the same height as the vials.
15) Focus on the surface of the microfluidic flow chamber with the 10X objective.
16) Clamp the waste tubing and all ink tubes except the wash tube.
17) Increase pressure on the flow controller for the wash vial to 5 PSI. Then open the wash valve and leave the wash valve open until all remaining air is removed from the microfluidic flow chamber. To verify all air is removed, look through the microfluidic flow chamber using the microscope.
18) Return the pressure on the flow controller to 3 PSI and stop the flow of the wash solution by closing the valve.
19) Unclamp the waste, ink, and solution tubes.
20) Move field of view of the camera to the patterning channel within the microfluidic flow chamber and refocus if necessary. Then adjust microscope focus to 50% of a full rotation of the focus knob below the channel focal plane for patterning.
21) Run the MAPDH-LC script.
    - MAPDH-LC script
        - https://github.com/MishaRubanov/MAPDH/tree/main/Supplemental%20protocols%20and%20data
    - The MAPDH-LC script runs the following tasks:
        - Flow ink 1 (green domain) solution into the lift-off flow chamber.
        - Pattern 50 green-domain hydrogels (200 µm squares) within specified locations.
        - Flow in 1x TAEM solution to remove residual ink solution.
        - Image hydrogels at specified locations.
        - Flow ink 2 (red domain) solution into the lift-off flow chamber.
        - Pattern 50 hydrogels (200 µm squares) to the left of each green-domain hydrogel such that there is a 10 µm overlap.
        - Flow in 1x TAEM solution to remove residual ink solution.
        - Image hydrogels at specified locations.
        - Flow ink 3 (blue domain) solution into the lift-off flow chamber.

- Pattern 50 hydrogels (200 µm squares) to the right of each green-domain hydrogel such that there is a 10 µm overlap.
- Flow in 1x TAEM solution to remove residual ink solution.
- Image hydrogels at specified locations.
- Flow in 1x TAEM solution to remove residual ink solution.
- Image hydrogels at specified locations.
- Flow in 1M NaCl solution for 1 minute to initiate dissolution of the PAA sacrificial layer.
- Image hydrogels at specified locations.
- Wait 1 hour.

22) Replace the outlet Falcon tube connected to the waste receptacle with a 1.7 mL microcentrifuge tube for collection.
23) Flow in 1X TAEM solution to convectively remove detached hydrogels.
24) Image hydrogel patterning locations to determine percentage of hydrogels that successfully detached.
25) Centrifuge the 1.7 mL microcentrifuge tube for 10-20 seconds at 6000 RPM.
26) Pipette 20 mL from the bottom of the centrifuged solution into a well of a 96-well plate.
27) Image collected hydrogels within the 96-well plate.

We used the lift-off flow chambers (**SI Section 20**) for patterning, lift-off, and collection of DNA-functionalized hydrogels. After turning on all the necessary equipment and software (step 1) (**SI Section 30**), we set up the lift-off flow chamber by inserting the waste tubing first (step 2). The waste tubing had a larger diameter compared to the ink/wash tubing (0.02"x0.06") to ensure low resistance of the outlet so that fluid prefers to flow through the waste tubing rather than through the ink/wash tubing. 24 inches of tubing was used to ensure that there was enough tubing to reach the waste receptacle.

The DNA crosslink sequences were those reported previously by Cangialosi et al. as System 1 strands [5] (step 3). System 1 and 2 DNA crosslink strands (S1 and S1'; S2 and S2') (sequences in **SI Section 34**) are purchased lyophilized from Integrated DNA Technologies (IDT) without additional purification. We diluted the DNA crosslink to 3 mM per strand, then followed an annealing protocol where the strands are mixed and heated to 90 °C to completely denature the strands, and slowly cooled to 20°C so that the two DNA strands can hybridize correctly (step 3). We used PEGDA 10K M_n in the ink formulation (step 4) due to higher-degree swelling compared to PEGDA 575 M_n [7].

We made three ink solutions each containing different fluorophore-modified DNA and DNA crosslink mixtures. The green domain does not contain and DNA crosslinks so that it does not undergo swelling after addition of system 1 or 2 hairpins (step 4). The solutions were then aliquoted to a vial that are then connected to the low-volume flow controller (step 8). We then added solutions containing 1x TAEM and 1 M NaCl to the other two vials (steps 6-7). Each vial had a modified lid (step 8) with one hole through which a hollow point needle connected to an air source (for pressure) was inserted and one hole through which the low volume ink/wash tubing (0.01"x0.03") fed through to the bottom of the vial for flow of solution from the bottom of the vial to the microfluidic flow chamber (**SI Section 3**). We used small-diameter ink/wash tubing to minimize the volume that was retained in the tubing and to increase the resistance to flow, which helped prevent backflow from one inlet into another.

For each inlet, we set the flow controller pressures to 3 PSI (step 9)—a value chosen to give moderate flow through the microfluidic flow chamber. Before connecting the tube to the microfluidic flow chamber , we primed the tube by opening the valve on the flow controller and letting the tubing fill with liquid until a bead of liquid was at the end of the tube (step 8). The tube for the wash solution was inserted last (*i.e.,* after all of the ink solutions) to ensure no ink remained within the microfluidic flow chamber during the rest of

the setup process (steps 10-13). The ink solutions and a waste receptacle were placed at the same level above the stage of the microscope to balance the hydrostatic pressure between the fluid source and the outlet of the tubing, and to drive flow down into the microfluidic flow chamber. After placing the microfluidic chamber on the stage (step 14), we used the 10X objective to focus on the flow chamber channel where we wanted to pattern (step 15) and then checked for air in the whole microfluidic flow chamber by moving the stage around. If air was visible, then we degassed the microfluidic flow chamber. This was done by clamping the waste tube and all the ink tubes except the wash solution tube (step 16), which meant we could increase the pressure inside of the microfluidic flow chamber, since fluid could not move out of the outlet or the other inlets, causing the air to compress and diffuse through the PDMS. After clamping everything except the wash solution tube, we opened the valve to the wash, increased the pressure to the wash vial on the flow controller to 5 PSI, and waited for the air in the microfluidic flow chamber to diffuse out through the PDMS. This usually took a few minutes (steps 17-19).

To prepare for patterning, we focused on the channel of the microfluidic flow chamber through the camera and then moved 50% of a full rotation of the focus knob (*i.e.*, the location of the objective relative to the patterning chamber) below the PDMS: PDMS interface as focused on by the camera (step 20). The plane that the camera is focused on was lowered to allow the UV light coming from the digital micromirror microfluidic flow chamber to be in focus, which allowed for hydrogels patterned at that focal plane to have well-defined features (**SI Figure 3A**). This is due to the different locations for the DMD and camera on the microscope.

The green domain was first patterned, and the locations for the 20 green domain hydrogels were used to determine the locations to pattern hydrogels within the red and blue domains (step 21). The red and blue domains were patterned with a 10-micron overlap with the green domain to ensure that the different domains remain connected to one another after lift-off and collection. We used 1 M of NaCl solution to dissolve the sacrificial layer by converting the PAA into its water-soluble form via Na+ ion exchange (step 21). During the 1 hour wait step after flowing in 1M NaCl, we replaced the waste receptacle with a 1.7 mL microcentrifuge tube so that for the next wash step, the hydrogels are collected in the 1.7 mL microcentrifuge tube rather than the waste receptacle (step 22). After an hour of waiting for the PAA layer to dissolve, we flow in 1 mL of 1x TAEM solution to convectively remove detached hydrogels and collect them in the microcentrifuge tube (step 23). We imaged the original patterning locations for all hydrogels between wash steps to determine the percentage of detached hydrogels at each round of patterning (step 24).

To concentrate the hydrogels, we centrifuge the 1.7 mL microcentrifuge tube at a spin rate of 6000 RPM, allowing collection of the settled hydrogels from the bottom of the tube (step 25). We can then aliquot 20 mL from the bottom of the collected solution into a well within a 96-well plate for further (step 26). Out of the 50 multi-domain hydrogels we patterned, we were only able to collect one intact 3-domain hydrogel in the 96-well plate.

30. Protocol for swelling of multi-domain hydrogels

1. Dilute System 1 hairpins (S1_H1 and S1_H2) and System 2 hairpins (S2_H1 and S2_H2) to a concentration of 200 mM per strand in 1x TAEM buffer.
2. Heat S1_H1 and S1_H2 DNA solutions to 95 °C for 15 mins using an Eppendorf PCR instrument.
3. Quickly place the heated strands in an ice bath for 5 min.
4. Pipette S1_H1 and S1_H2 into the well that contains the 3-domain hydrogel for a final concentration of 20 mM of each hairpin.

5. Run time-lapse fluorescence imaging with 30-minute intervals to measure change in red hydrogel length over 24 hours.
6. Heat S2_H1 and S2_H2 DNA solutions to 95 °C for 15 mins using an Eppendorf PCR instrument.
7. Quickly place the heated strands in an ice bath for 5 min.
8. Pipette out 60 mL of solution above the hydrogels.
9. Pipette a 60 mL solution of 20 mM S2_H1 and 20 mM S2_H2 in 1x TAEM buffer.
10. Run time-lapse fluorescence imaging with 30-minute intervals to measure change in blue hydrogel length for 24 hours.

System 1 (S1_H1, S1_H2) and System 2 (S2_H1, S2_H2) hairpins were purchased in a lyophilized form from IDT without additional purification. All four ssDNA strands from system 1 and system 2 swelling signals were diluted to 100 mM in 1X TAEM buffer (step 1). For the first swelling stage, only the S1_H1 and S1_H2 DNA solution were first heated and cooled in an ice bath to form the correct hairpin structure (steps 2-3). After formation of the hairpins, 20 mL of each S1_H1 and S1_H2 DNA solutions were pipetted into the well that contains the 3-domain hydrogel (step 4). We then added 40 mL of 1x TAEM buffer solution for a total volume of 100 mL. This way we have 20 mM of S1 DNA swelling signals inside the well to induce actuation of the red hydrogel. We ran time-lapse fluorescence imaging of the multi-domain hydrogel swelling for 24 hours with 30-minute intervals to observe time-dependent changes in red domain length (steps 4-5). After 24 hours, we repeated the above process using system 2 hairpins to actuate the blue domain hydrogel (steps 5-9).

31. Analysis for multi-domain hydrogel swelling

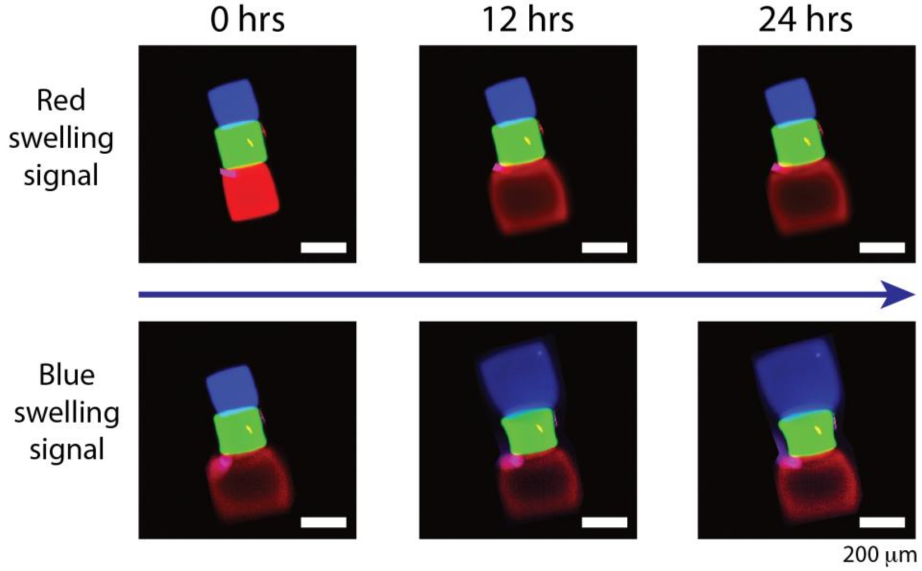

Figure 23. Analysis of multi-domain hydrogel swelling. Micrographs of multi-domain hydrogel swelling after addition of red and blue swelling signals.

Swelling of the free-floating multi-domain hydrogel was conducted for 48 hours with imaging via time-lapse fluorescence at 30-minute intervals. After imaging, we overlaid the TYE665, Cy3, and ATTO488 fluorescent channels and assigned each grayscale micrograph a color channel within a 3-color composite micrograph. The Cy3 micrograph was assigned to the red channel, the TYE665 micrograph was assigned to the blue channel, and the ATTO488 micrograph was assigned to the green channel. **SI Figure 23** depicts

swelling profiles of the hydrogels specifically at 0, 12, and 24 hours after addition of the red/blue swelling signal into the well.

We quantitatively analyze swelling of the free-floating multi-domain hydrogels by using the automated length measurement algorithm described in **SI Section 27**. Here, we analyzed micrographs taken from each individual filter (red, green, or blue) to compare hydrogel length changes for each domain over time. Similarly, for each hydrogel domain, we binarized the micrograph, and used corner detection to determine length of the corners. After identifying the corners of each hydrogel, we determined the distance between each corner.

Swelling of the free-floating multi-domain hydrogel was analyzed and plotted as $\Delta L/L_0$ over time and smoothened by calculating and applying the moving average of eight values using the numpy.convolve module. Hydrogel swelling was calculated by comparing the corner-to-corner length of the hydrogel in each time-lapse micrograph to the initial corner-to-corner length of the hydrogel before addition of red and blue DNA swelling signals. This automated measurement protocol was applied to all three domains of the 3-domain hydrogel and the result is plotted in **Figure 5E**.

Code for this analysis can be found at https://github.com/MishaRubanov/MAPDH.

32. MAPDH Equipment

**Label**	Vendor/Part number	Sections used
DMD	Mightex Polygon 400	9,10,11,16,18,23,24,27,28
Microscope (Filter turrets,etc)	Olympus IX73	9,11,12,16,18,23,24,27,28
UV LED	CoolLED PE-100 365 nm	9,11,12,16,18,23,24,27,28
Electronic Valve	Grainger 4ELC3	9,11,12,16,18,23,24,27,28
Pressure Gauge	McMaster-Carr 2798K211 Range of 0-30psi	9,11,12,16,18,23,24,27,28
Pressure Regulator	Airtrol R-800 SERIES MINIATURE PRECISION PRESSURE REGULATOR – 1/8 NPT PORTS, 0.5-10psi	9,11,12,16,18,23,24,27,28
10x Objective	Olympus UPlanFL N 10X/0.30 NA	9,11,12,16,18,23,24,27,28
20x NUV Objective	Thorlabs LMU-20X-NUV	11
Cy3 Filter	Laser2000 Cy3-4040C	9,12,16,18,25,26,29,30,
Atto488 Filter	Chroma Oregon Green 488, 89021	12,16,18,26,30
TYE665 Filter	Chroma 49006	12,16,18,26,30
Dichroic Mirror	Mightex 90/10 Beam splitter (DSI-BS-90R-10T-000)	9,11,12,16,18
XY Stage	ASI MS-2000 Flat-top	9,11,12,16,18
White LED	X-Cite 120LED Boost High-Power	9,12,16,18,
Light Pillar	Olympus IX73 Universal Condenser	9,11,12,16,18
Camera	Hamamatsu Flash 4.0LT	9,11,12,16,18
Vacuum Chamber	BEL-ART F42025-0000	8,20,21,27,28
Biopsy Punch—0.75mm	Ted Pella, Inc. 15115-2	8,9,11,12,16,18,22,23,24
Biopsy Punch—1.5mm	Ted Pella, Inc. 15110-15	8,22
Tesla Coil	BD-20 AC Laboratory Corna Treater (Electro Technic Products SKU: 12051A)	8,20,22

Plasma Oven	Technics PE-II-A Etcher & Stripper	6,7,20
Double sided mask aligner	EV group EVG620 NT	6,7
Microcentrifuge	Corning LSE Mini Microcentrifuge 6770	23,24,27,28

33. MAPDH Reagents and components

**Label**	Full name	Vendor	Part Number	Sections used
Vial	Conical Vial	Fisher	3431ANK	9,11,12,16,18,23,24, 27,28
PEGDA-575	Poly-ethylene Glycol Diacrylate- 575 M_n	Sigma-Aldrich	437441	9,11,12,16,18,23,24, 1,1
LAP	Lithium phenyl-2,4,6-trimethylbenzoylphosphinate	Allevi	LAP	9,11,12,16,18,23,24, 27,28
TAEM	Tris, acetic acid, EDTA, Mg^{2+}	Sigma-Aldrich	1.06174	9,11,12,16,18,23,24, 25,26,27,28,29,30
NaCl	Sodium Chloride	Fisher Chemical	7647-14-5	23,24,27,28
PDMS	Polydimethylsiloxane	Fisher	Dow Sylgard 185 NC9285739	8,20,21
Glass cover slip		Thermo Scientific Gold Seal Cover Glass 48x60mm No 1	VWR 48404-142	8
Silicon wafer		Wafer World Inc	2886	6,7,20
SU-8 3050 photoresist		Fisher	NC0702369	6,20
SU-8 developer		Fisher	NC9901158	6,7,20
IPA	Isopropyl alcohol			6,7,20
SU-8 10 photoresist		Fisher	NC1954729	7
Scotch tape				8
Tygon tubing (0.02”x0.06”)		Fisher	06419-01	9,11,12,16,18,23,24, 27,28
Tygon tubing (0.01”x0.03”)		Fisher	06419-00	9,11,12,16,18,23,24, 27,28
1mL syringe		VWR	BD309647<br>or<br>BD-309628	11,18
23-Gauge Blunt Tipped Needle		VWR	37695-168	11,18,23,24,27,28
Calcium Chloride		Sigma-Aldrich	C1016	21
PEDGA-10K	Poly-ethylene Glycol Diacrylate- 10k M_n	MilliporeSigma	729094	27,28
PCR thermocycler		Eppendorf	Mastercycler GX2e	27,28,29,30
200 mL PCR tube		VMR	20170-010	27,28,29,30

1.7 mL microcentrifuge tube		VMR	87003-294	23,24,27,28

34. Sequences

Name	Sequence	Purification	Sections used
5Acry_3TYE665_polyT10	/5ACryd/TTTTTTTTTT/3TYE665/	HPLC	12,16,18, 24,28
5Acry_3Cy3_polyT10	/5ACryd/TTTTTTTTTT/3Cy3Sp/	HPLC	9,12,16, 23,24,27,28
5Acry_3ATTO488_polyT10	/5Acryd/TTTTTTTTTT/3ATTO488N/	HPLC	12,16,18,24, 28
5Acry_3Cy3_R1	/5ACryd/ TGACCGTAGGCGAAGCACCTTTTTTGACATACAGATTAACCAGACA/3Cy3Sp/	HPLC	18
5Q_R1’	/5IABkFQ/TGTCTGGTTAATCTGTATGTCAAAAAAGGTGCTTCGCCTACGGTCA	HPLC	18
S1	/5Acryd/TAAGTTCGCTGTGGCACCTGCACG	Desalted	27,28
S1’	/5Acryd/CAA CGTGCAGGTGCCACAGCGTGG	Desalted	27,28
S1_H1	CCACGCTGTGGCACCTGCACGCACCCACGTGCAGGTGCCACAGCGAACTTA	Desalted	29,30
S1_H2	TGGGTGCGTGCAGGTGCCACAGCGTAAGTTCGCTGTGGCACCTGCACG TTG	Desalted	29,30
S2	/5Acryd/CTGTCTGCCTACCACTCCGTTGCG	Desalted	28
S2’	/5Acryd/ATTCGCAACGGAGTGGTAGGCTTT	Desalted	28
S2_H1	AAAGCCTACCACTCCGTTGCGGAACCTCGCAACGGAGTGGTAGGCAGACAG	Desalted	30
S2_H2	AGGTTCCGCAACGGAGTGGTAGGCCTGTCTGCCTACCACTCCGTTGCGAAT	Desalted	30

35. Bibliography

1. Edelstein AD, Tsuchida MA, Amodaj N, Pinkard H, Vale RD, Stuurman N. Advanced methods of microscope control using μManager software. Journal of Biological Methods. 2014;1: e10–e10. doi:10.14440/jbm.2014.36

2. Pinkard H, Stuurman N, Ivanov IE, Anthony NM, Ouyang W, Li B, et al. Pycro-Manager: open-source software for customized and reproducible microscope control. Nat Methods. 2021;18: 226–228. doi:10.1038/s41592-021-01087-6

3. KAM-SU-8-2-25-Datasheet-9.3.20-final.pdf. Available: https://kayakuam.com/wp-content/uploads/2020/09/KAM-SU-8-2-25-Datasheet-9.3.20-final.pdf

4. Dorsey PJ, Rubanov M, Wang W, Schulman R. Digital Maskless Photolithographic Patterning of DNA-Functionalized Poly(ethylene glycol) Diacrylate Hydrogels with Visible Light Enabling Photodirected Release of Oligonucleotides. ACS Macro Lett. 2019; 1133–1140. doi:10.1021/acsmacrolett.9b00450

5. Cangialosi A, Yoon C, Liu J, Huang Q, Guo J, Nguyen TD, et al. DNA sequence–directed shape change of photopatterned hydrogels via high-degree swelling. Science. 2017;357: 1126–1130. doi:10.1126/science.aan3925

6. Linder V, Gates BD, Ryan D, Parviz BA, Whitesides GM. Water-Soluble Sacrificial Layers for Surface Micromachining. Small. 2005;1: 730–736. doi:10.1002/smll.200400159

7. Otsu N. A threshold selection method from gray-level histograms. IEEE Transactions on Systems, Man and Cybernetics. 1979;9(1): 62-6.

8. Shi R, Fern J, Xu W, Jia S, Huang Q, Pahapale G, et al. Multicomponent DNA Polymerization Motor Gels. Small. 2020;16: 2002946. doi:10.1002/smll.202002946
