# Supplementary figures and images for: Multi-domain automated patterning of DNA-functionalized hydrogels
Source: PLoS One. 2024 Feb 2;19(2):e0295923. doi: 10.1371/journal.pone.0295923 (PMC10836684; doi:10.1371/journal.pone.0295923)

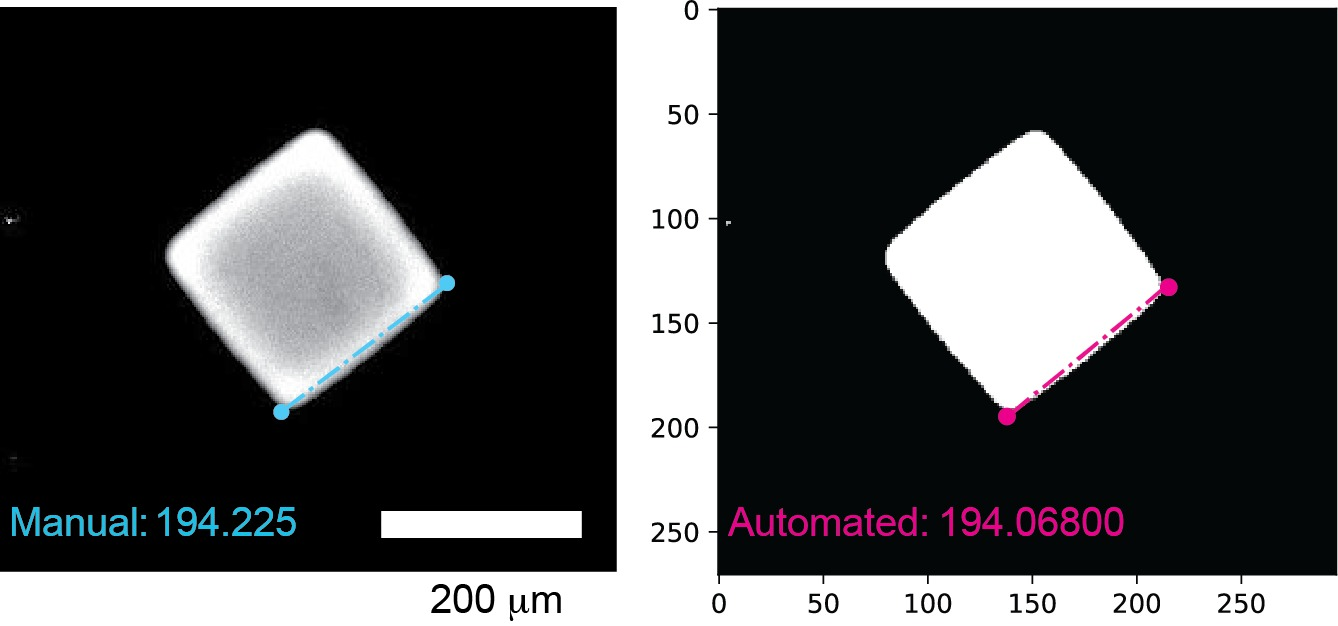

Supplement: S1 Fig — Pressurized air is fed into the top of the air-tight vial through the air tube, which forces ink through the ink/wash tube out of the vial and into the microfluidic flow chamber. (TIF) [file pone.0295923.s002.tif]

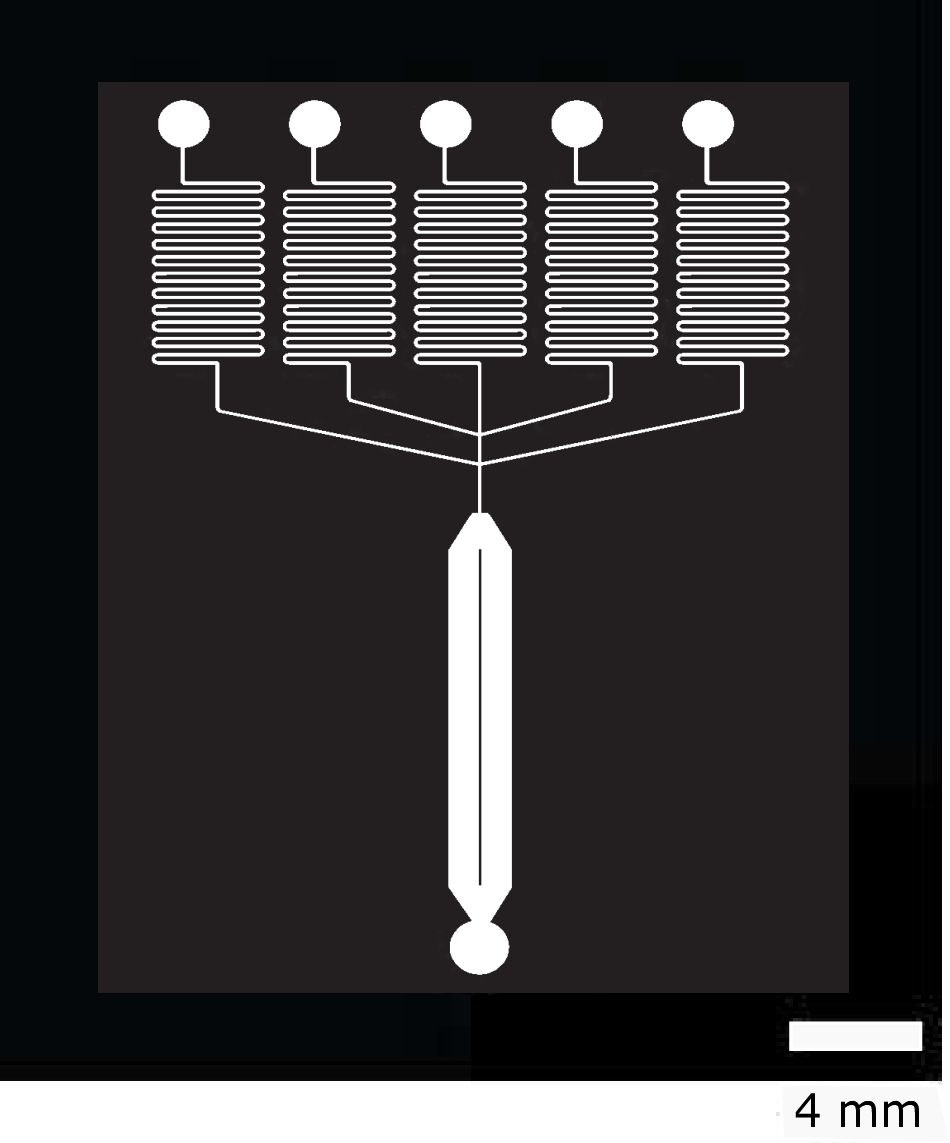

Supplement: S2 Fig — This microfluidic flow chamber was designed using AutoCAD. The design was used to generate a mask (5) produced by Fineline Imaging. (TIF) [file pone.0295923.s003.tif]

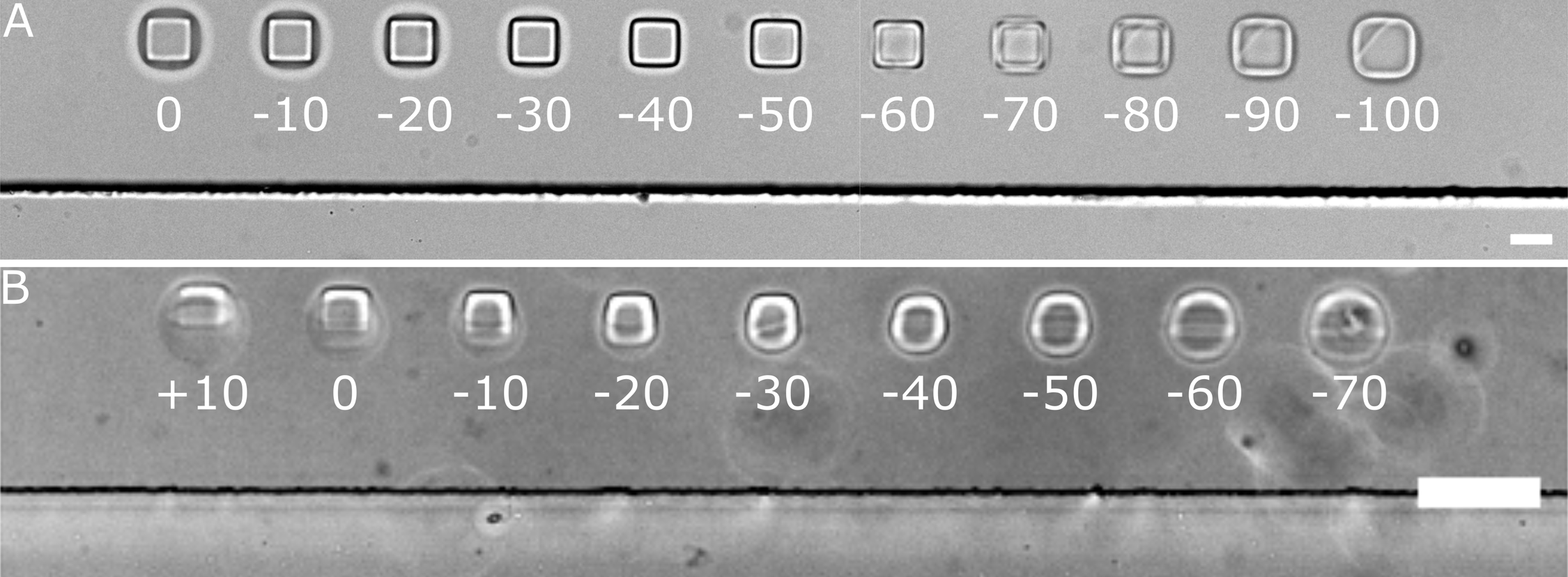

Supplement: S3 Fig — The labels below each hydrogel are the percentages of a full rotation of the microscope, relative to focusing the camera on the glass surface-PDMS interface that the objective was set to before patterning. Since the DMD and camera are in different locations within the microscope and thus have different planes where they are in focus, we chose the focal plane for patterning by first focusing on the z-plane of the camera, and then adjusting the z-plane to match the ideal plane for patterning. A) 10x patterning and imaging. We observed that the sharpest edges were made when patterning at a plane 50% of a full turn below the plane where the camera is focused on the glass: PDMS interface of the microfluidic flow chamber. B) Patterning and imaging using the 20x objective. We observed that the sharpest edges were found when patterning at a plane 30% of a full turn below the plane where the camera is focused on the glass: PDMS interface of the microfluidic flow chamber. Scale bars are 50 μm. (TIF) [file pone.0295923.s004.tif]

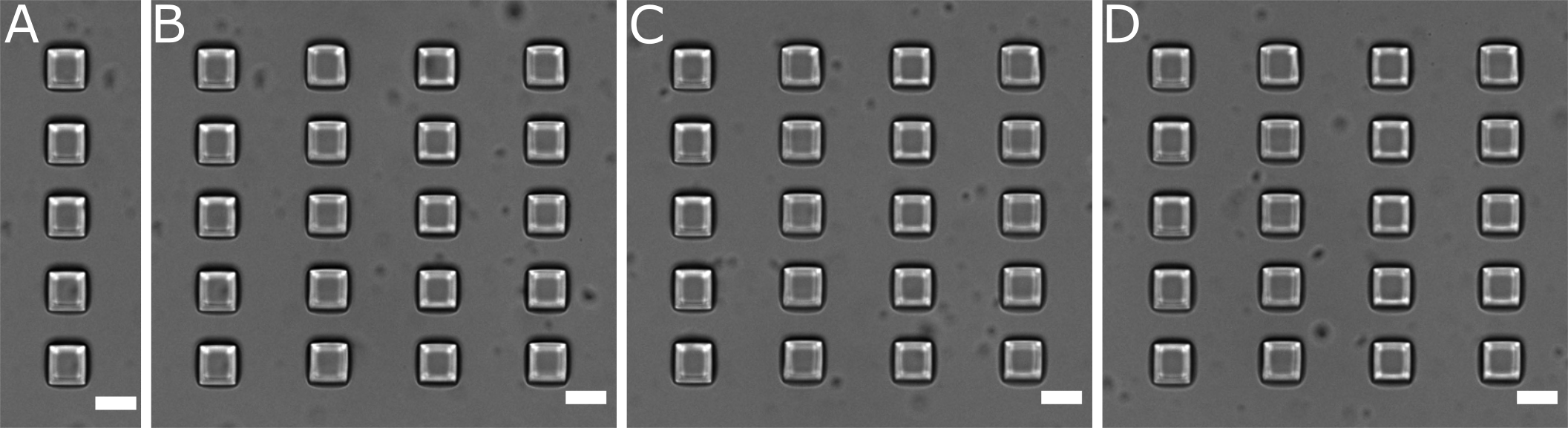

Supplement: S4 Fig — The first micrograph shows the hydrogels produced within a single round of patterning (Set 1). The last three micrographs show the resulting hydrogels from all 12 rounds of patterning, in order from left to right. Scale bars are 50 μm. (TIF) [file pone.0295923.s005.tif]

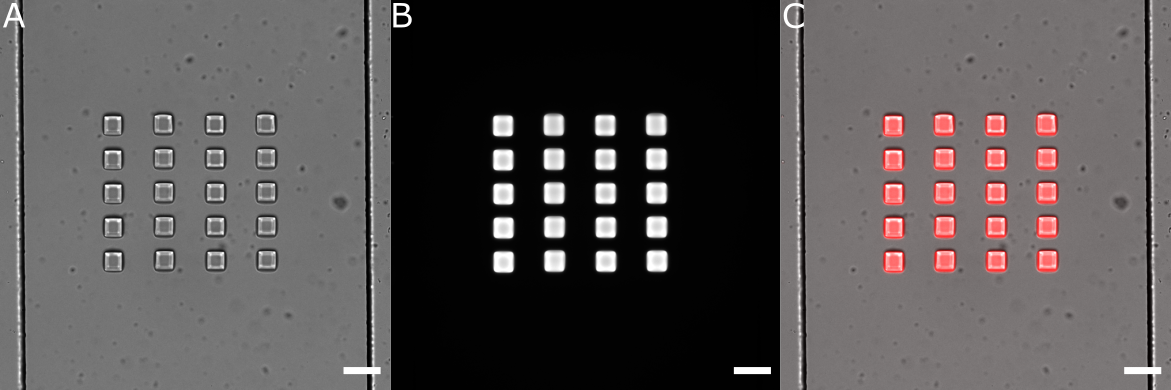

Supplement: S5 Fig — A) Bright field image B) Fluorescence image (Cy3) C) Overlay with brightfield grayscale and Cy3 in red. Scale bars are 100 μm. (TIF) [file pone.0295923.s006.tif]

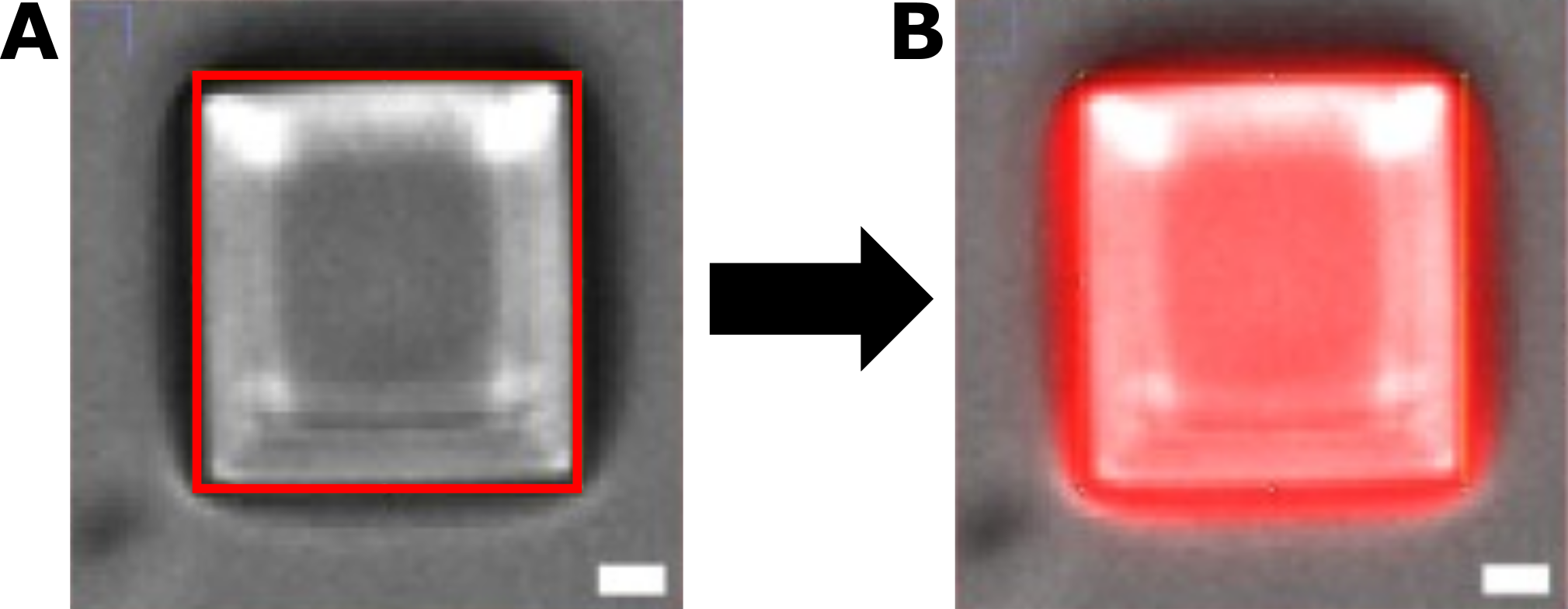

Supplement: S6 Fig — A) Brightfield micrograph for a single hydrogel. The outline was chosen manually using ImageJ’s rectangle selection tool. The width and height of this rectangle corresponded to the width and height of the hydrogel. B) Overlayed brightfield and fluorescent Cy3 micrographs for a single hydrogel. The rectangle, selected from the brightfield micrograph in S6A Fig, is used to calculate the average fluorescence intensity for the hydrogel. Scale bars are 10 μm. (TIF) [file pone.0295923.s007.tif]

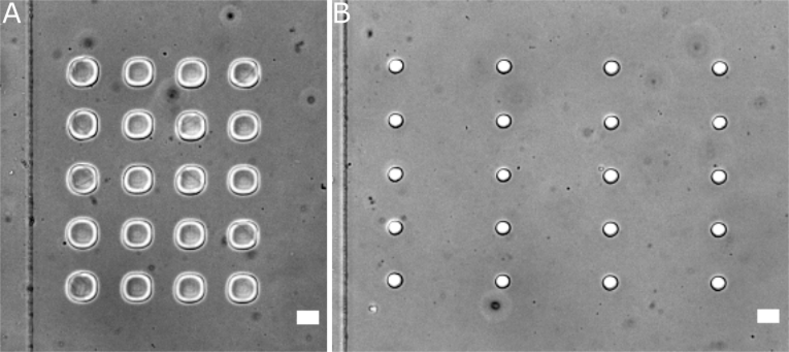

Supplement: S7 Fig — A) Hydrogels patterned automatically using 20 μm square masks and Inks 1–4 following the Section 9 in S1 File protocol. B) Hydrogels patterned automatically using 10 μm square masks and Inks 1–4 following the Section 9 in S1 File protocol. Scale bars are 20 μm. (TIF) [file pone.0295923.s008.tif]

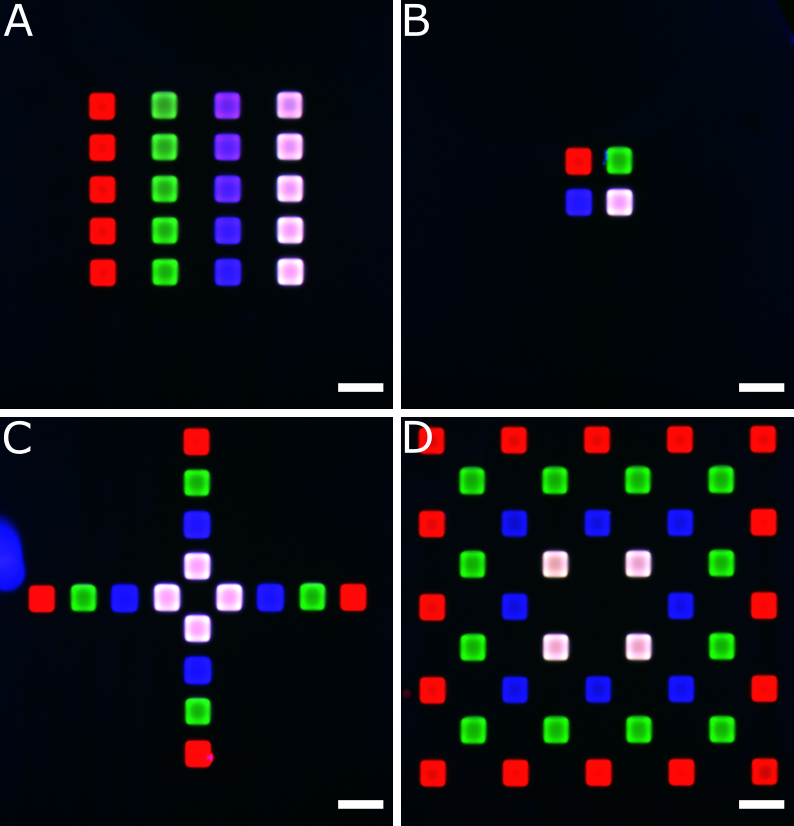

Supplement: S8 Fig — A) Architecture 1, B) Architecture 2, C) Architecture 3, D) Architecture 4. Scale bars are 100 μm. (TIF) [file pone.0295923.s009.tif]

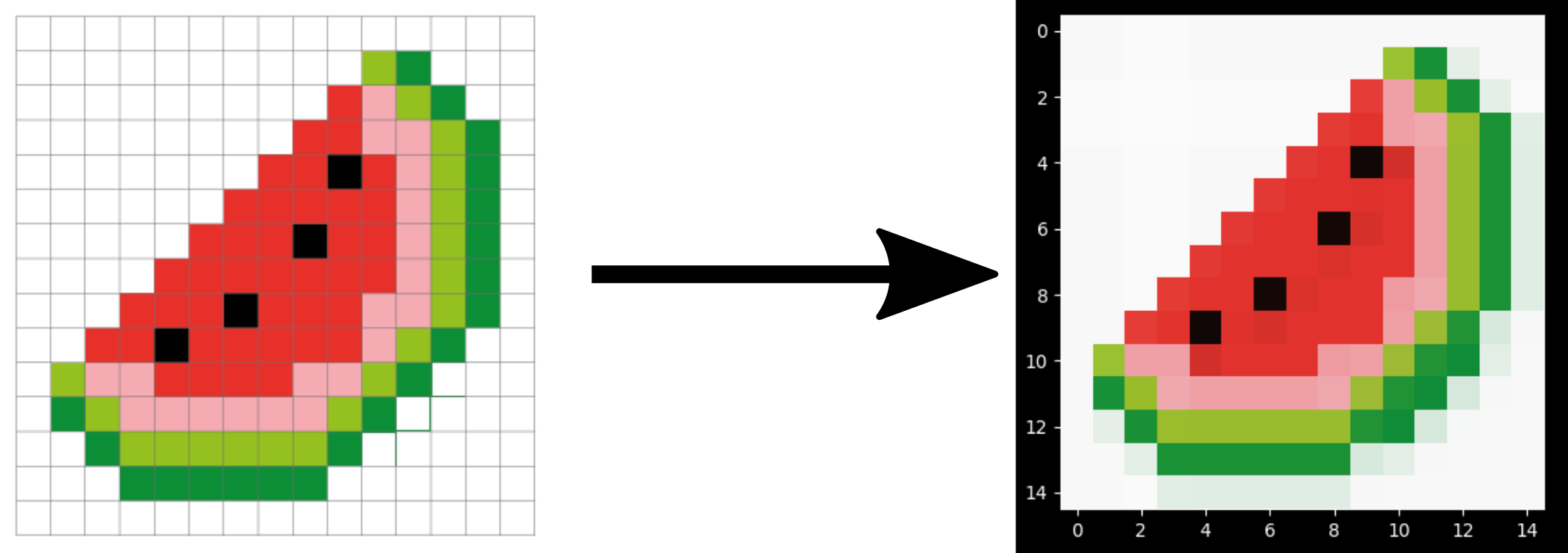

Supplement: S9 Fig — The left image is a 355x355 pixel image, the right is a binned 15x15 version of the same image. (TIF) [file pone.0295923.s010.tif]

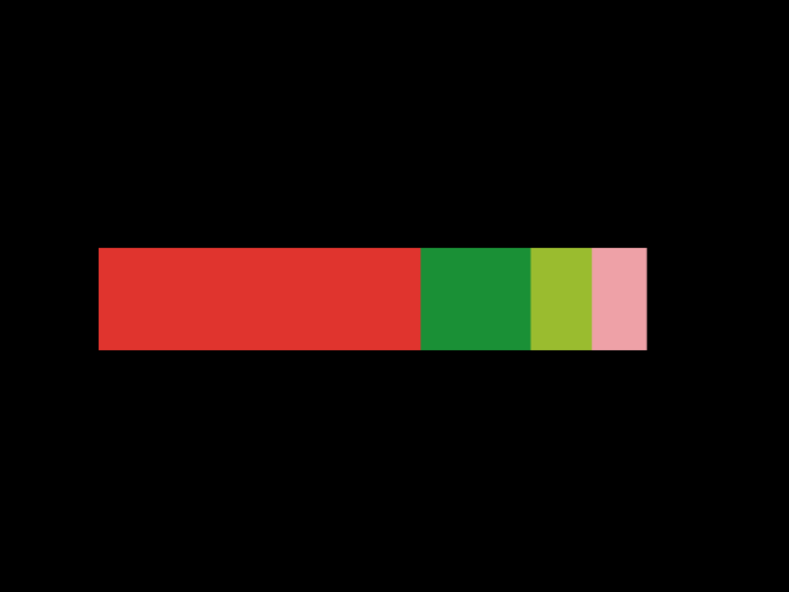

Supplement: S10 Fig — The length of each color bar represents the number of pixels with that corresponding color. The colors were clustered automatically using the k-means clustering method. (TIF) [file pone.0295923.s011.tif]

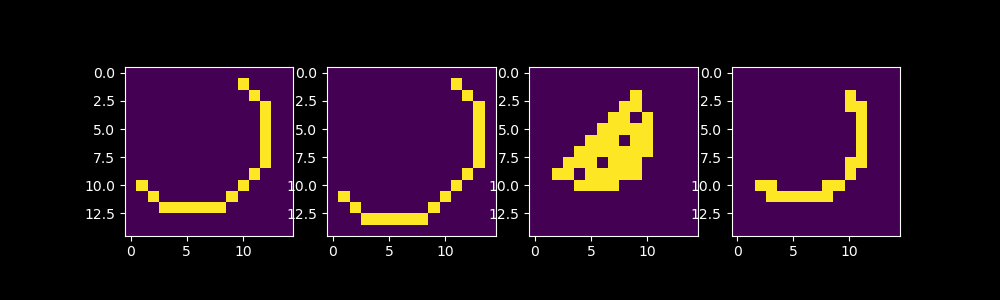

Supplement: S11 Fig — These masks, termed location maps, serve as inputs for the MAPDH function matrix_patterner in Section 13 in S1 File. (TIF) [file pone.0295923.s012.tif]

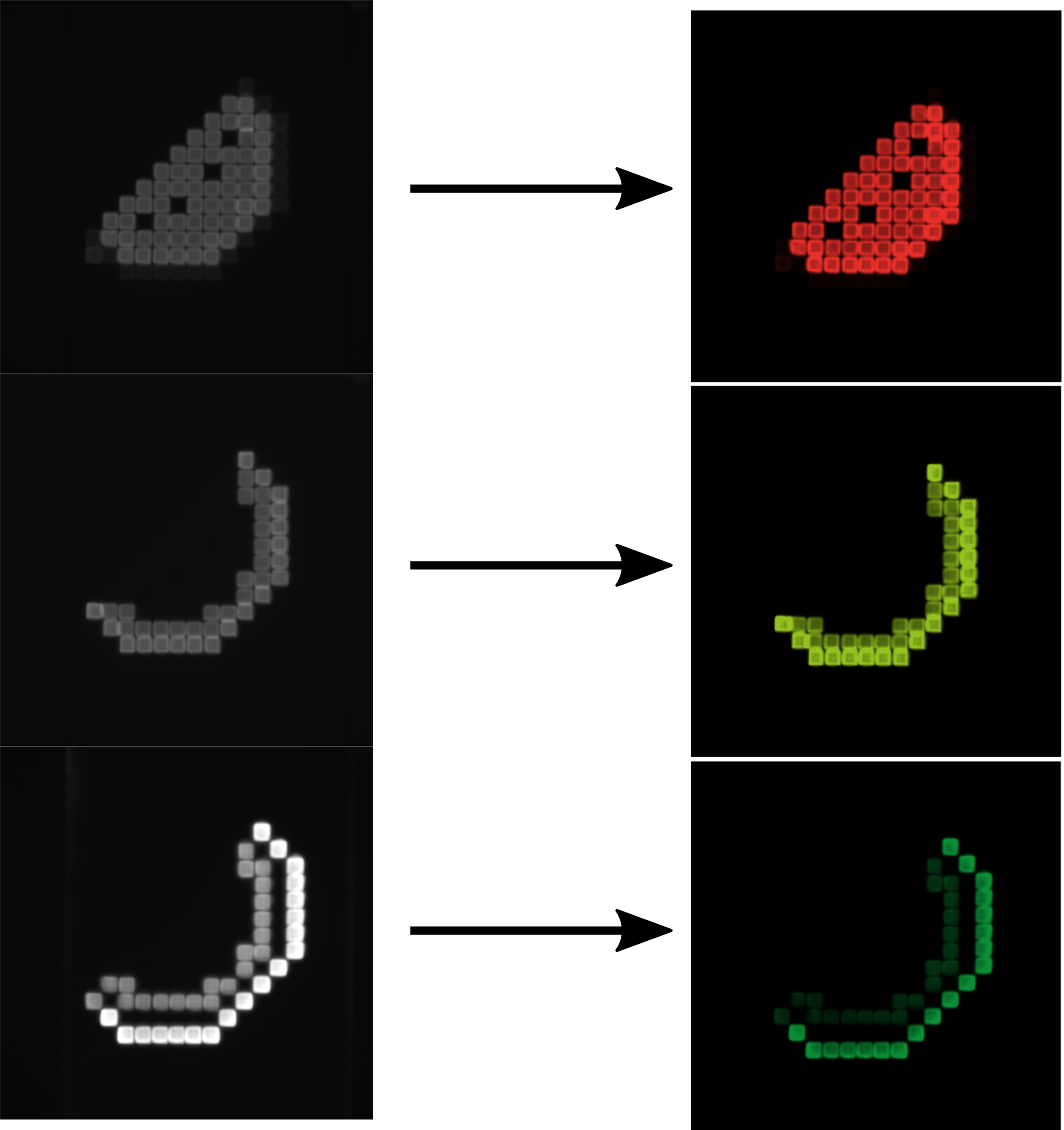

Supplement: S12 Fig — (TIF) [file pone.0295923.s013.tif]

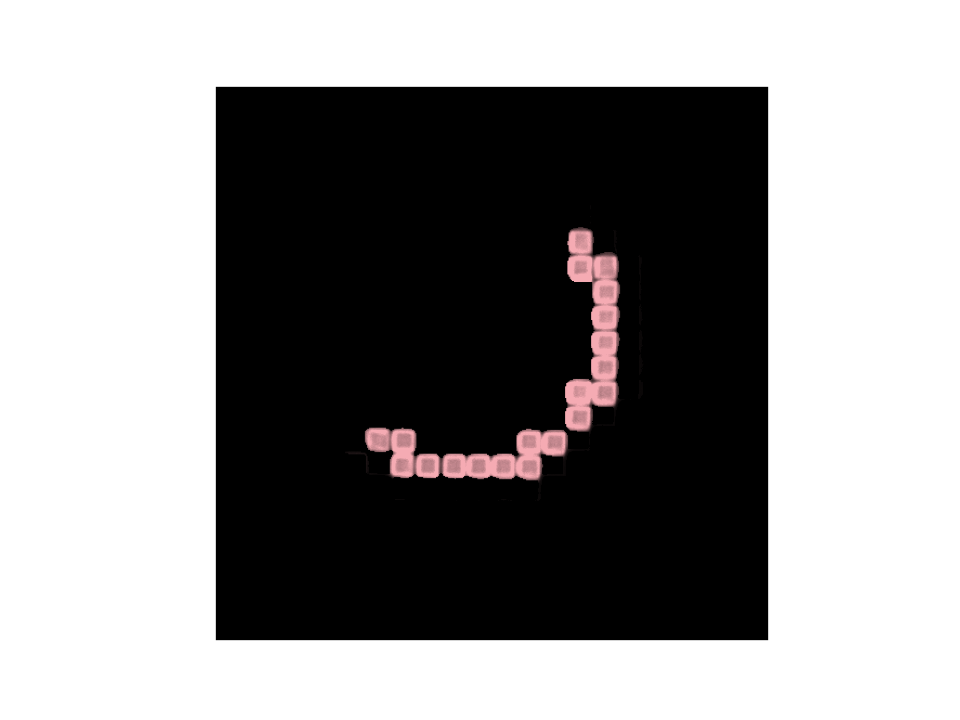

Supplement: S13 Fig — Enhanced and colorized. (TIF) [file pone.0295923.s014.tif]

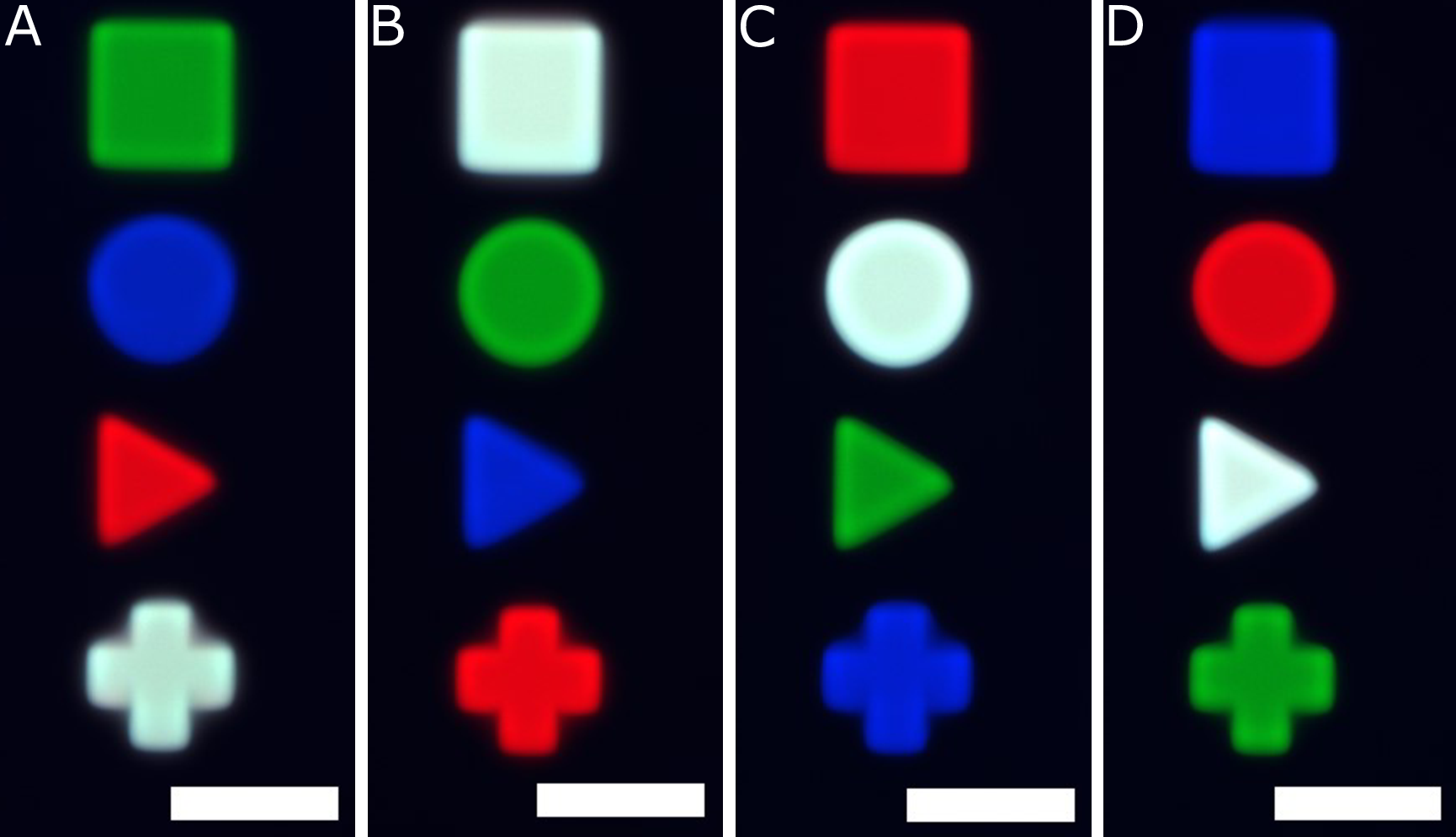

Supplement: S14 Fig — The TYE665 (red channel), Cy3 (green channel), and ATTO488 (blue channel) micrographs are overlaid as described in Section 17 in S1 File. A) Set 1 B) Set 2 C) Set 3 D) Set 4 as described in Section 16 in S1 File. Scale bars are 100 μm. (TIF) [file pone.0295923.s015.tif]

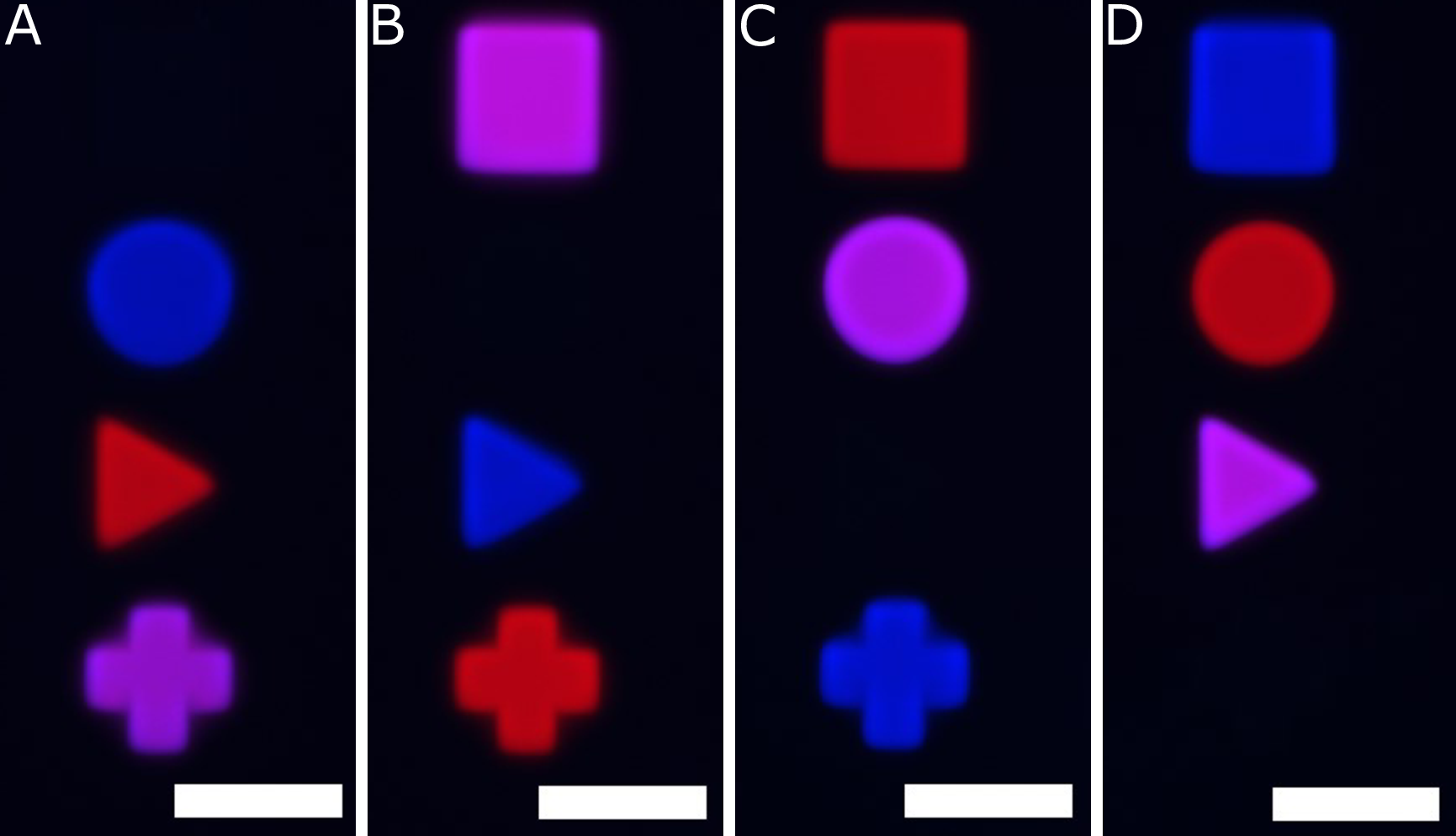

Supplement: S15 Fig — TYE665 (red channel), Cy3 (green channel), and ATTO488 (blue channel) micrographs overlaid as described in Section 17 in S1 File. A) Set 1 B) Set 2 C) Set 3 D) Set 4 as described in Section 16 in S1 File. Scale bars are 100 μm. (TIF) [file pone.0295923.s016.tif]

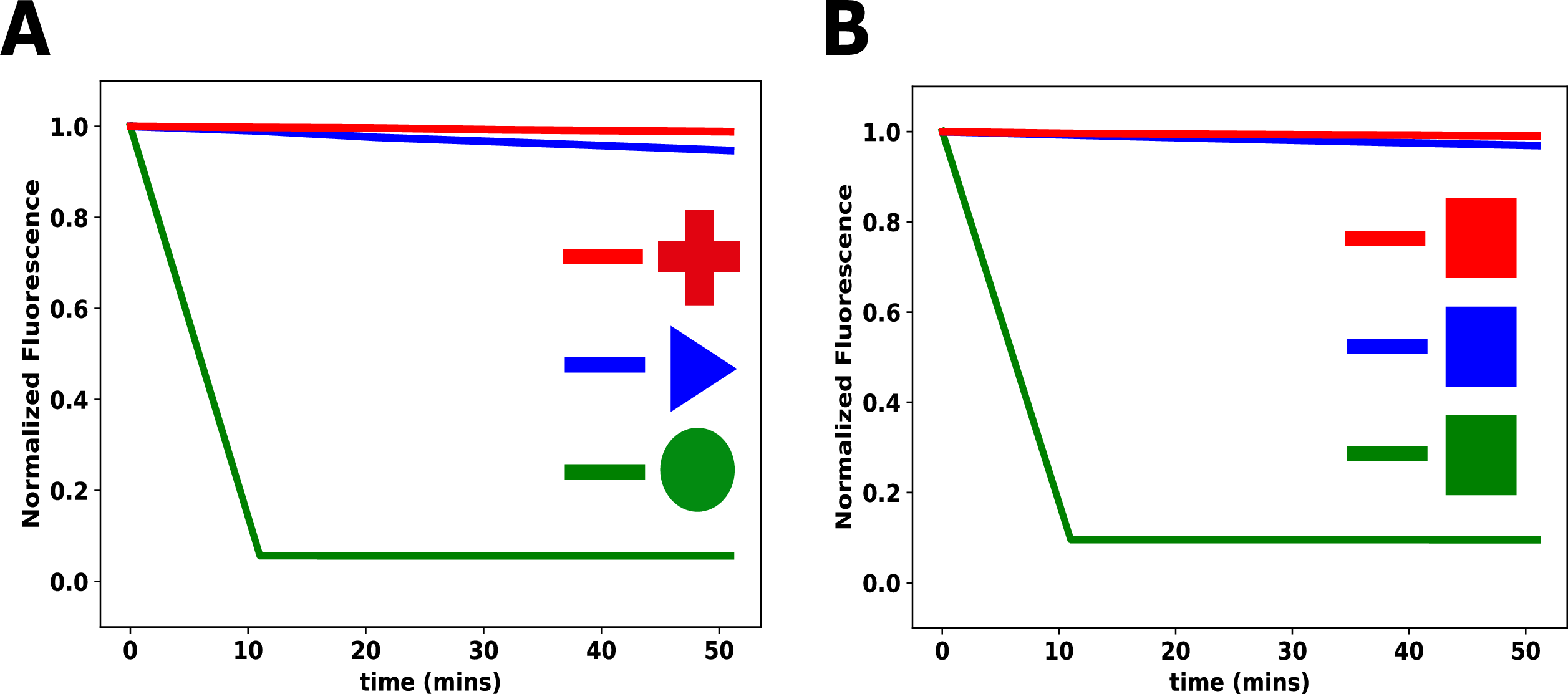

Supplement: S16 Fig — A) Normalized fluorescence for the plus, triangle, and circle hydrogels in the respective channels that contained fluorescence. B) Normalized fluorescence for all three fluorophore channels for the square hydrogel. (TIF) [file pone.0295923.s017.tif]

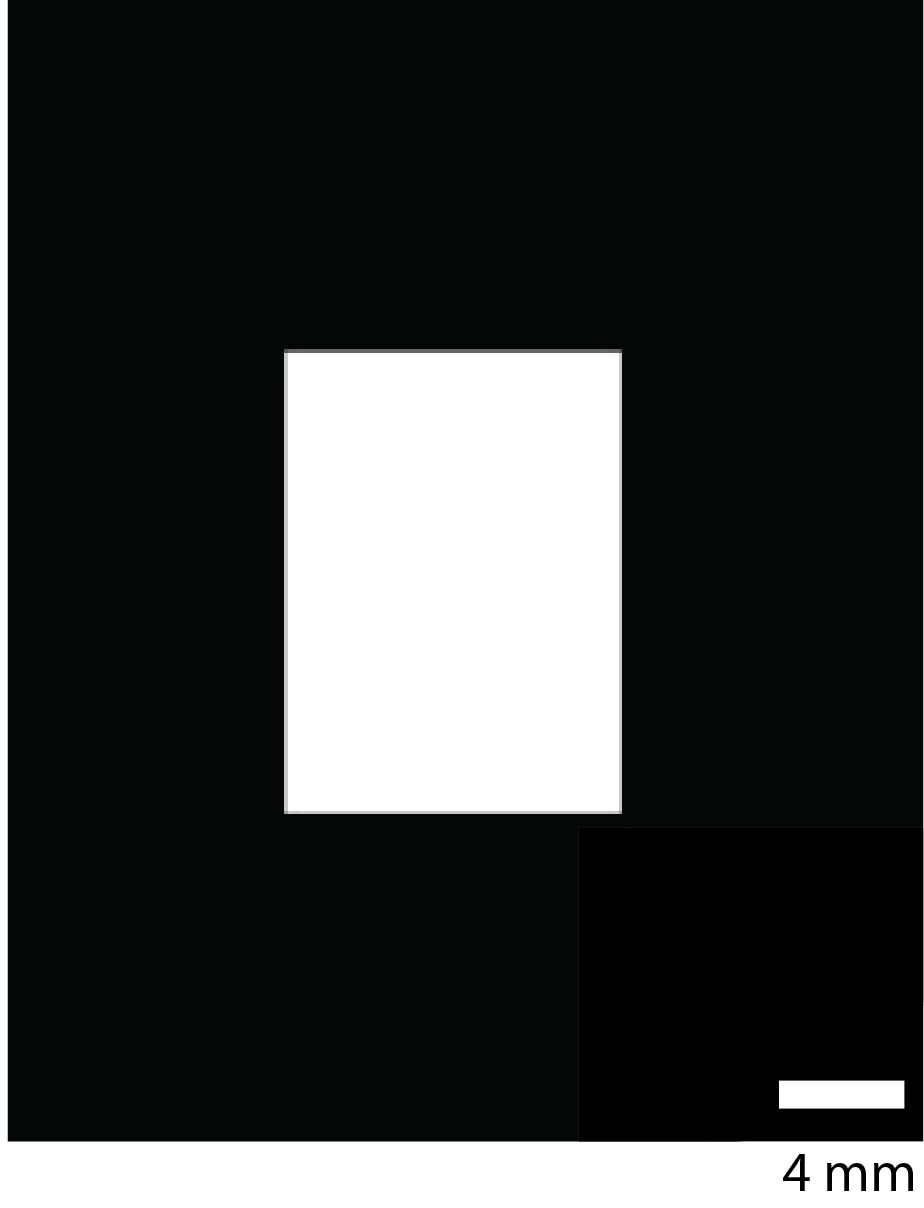

Supplement: S17 Fig — (TIF) [file pone.0295923.s018.tif]

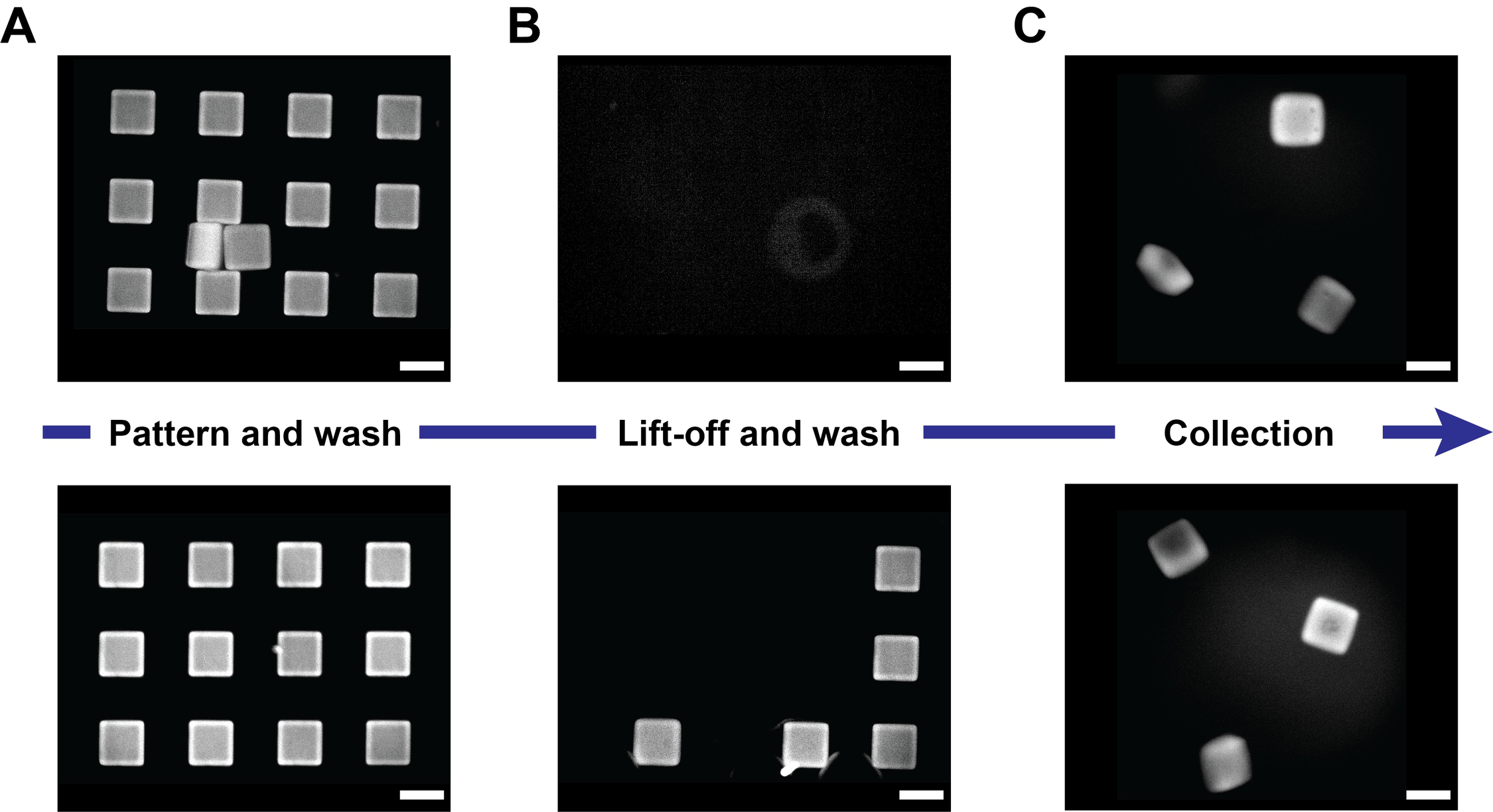

Supplement: S18 Fig — A) Photopatterning and washing. B) Lift-off and washing. C) Collection in a well of a 96-well plate. Top and bottom micrographs are two different locations from the same round of single-domain hydrogel patterning. Scale bars are 200 μm. (TIF) [file pone.0295923.s019.tif]

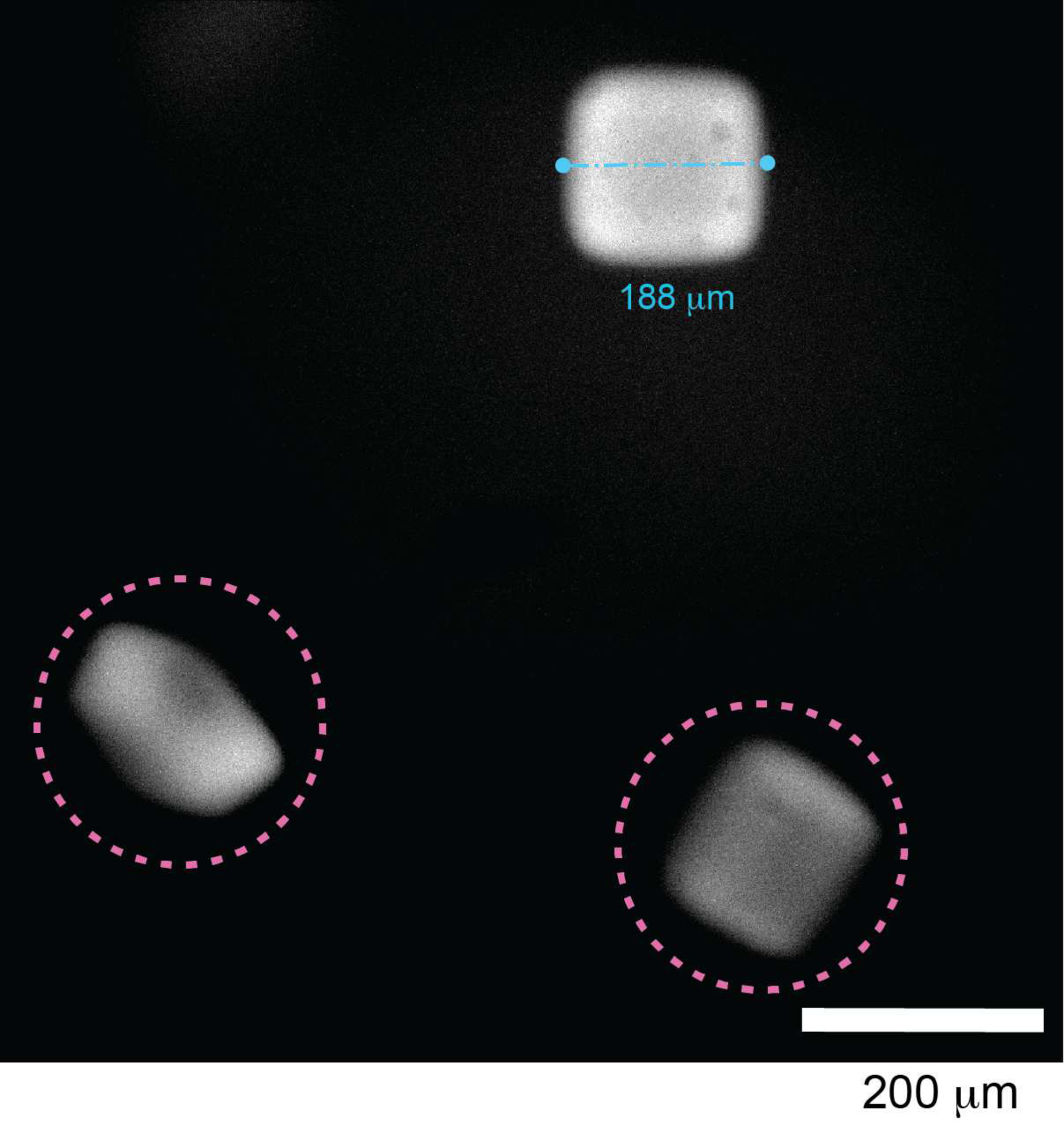

Supplement: S19 Fig — Hydrogels on their sides and/or not flat on the glass surface are enclosed by dashed, pink circles. Only hydrogels that lay flat on the bottom surface of the plate were further analyzed (top hydrogel). (TIF) [file pone.0295923.s020.tif]

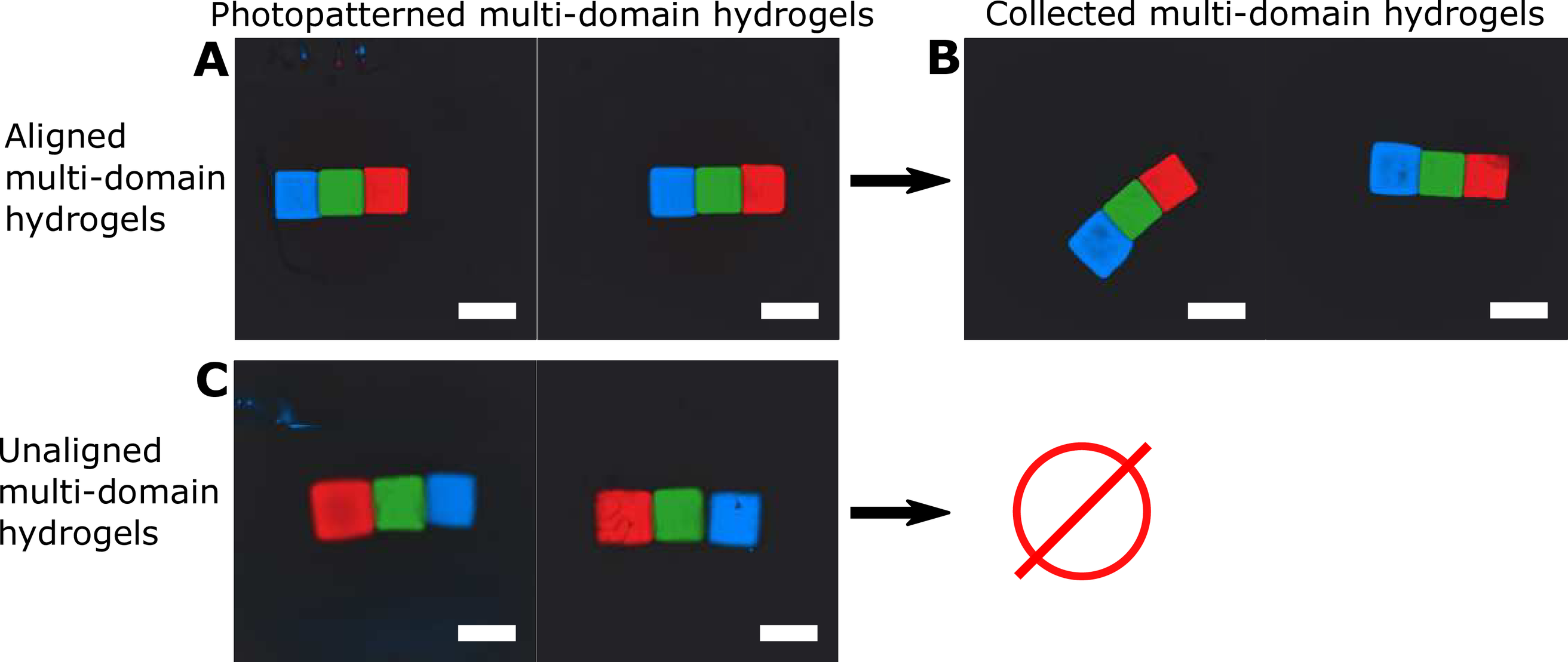

Supplement: S20 Fig — Out of the 13 aligned hydrogels 2 were successfully collected. A) Hydrogels in the microfluidic device. B) Hydrogels collected and placed in a well of a 96-well plate. C) Unaligned multi-domain hydrogels due to poor adhesion between the hydrogel and the sacrificial layer. Scale bars are 200 μm. (TIF) [file pone.0295923.s021.tif]

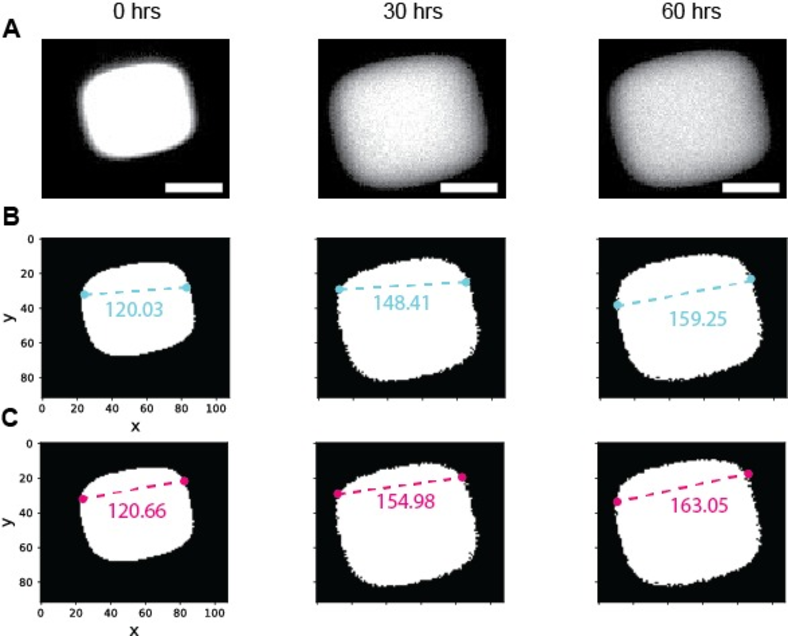

Supplement: S21 Fig — Demonstration of measuring hydrogel lengths and comparison of (left) manual and (right) automated measurements. Herein, to automatically measure the hydrogel length we binarized the micrograph by a threshold calculated via Otsu’s method. (Reference to MAPDH script). (TIF) [file pone.0295923.s022.tif]

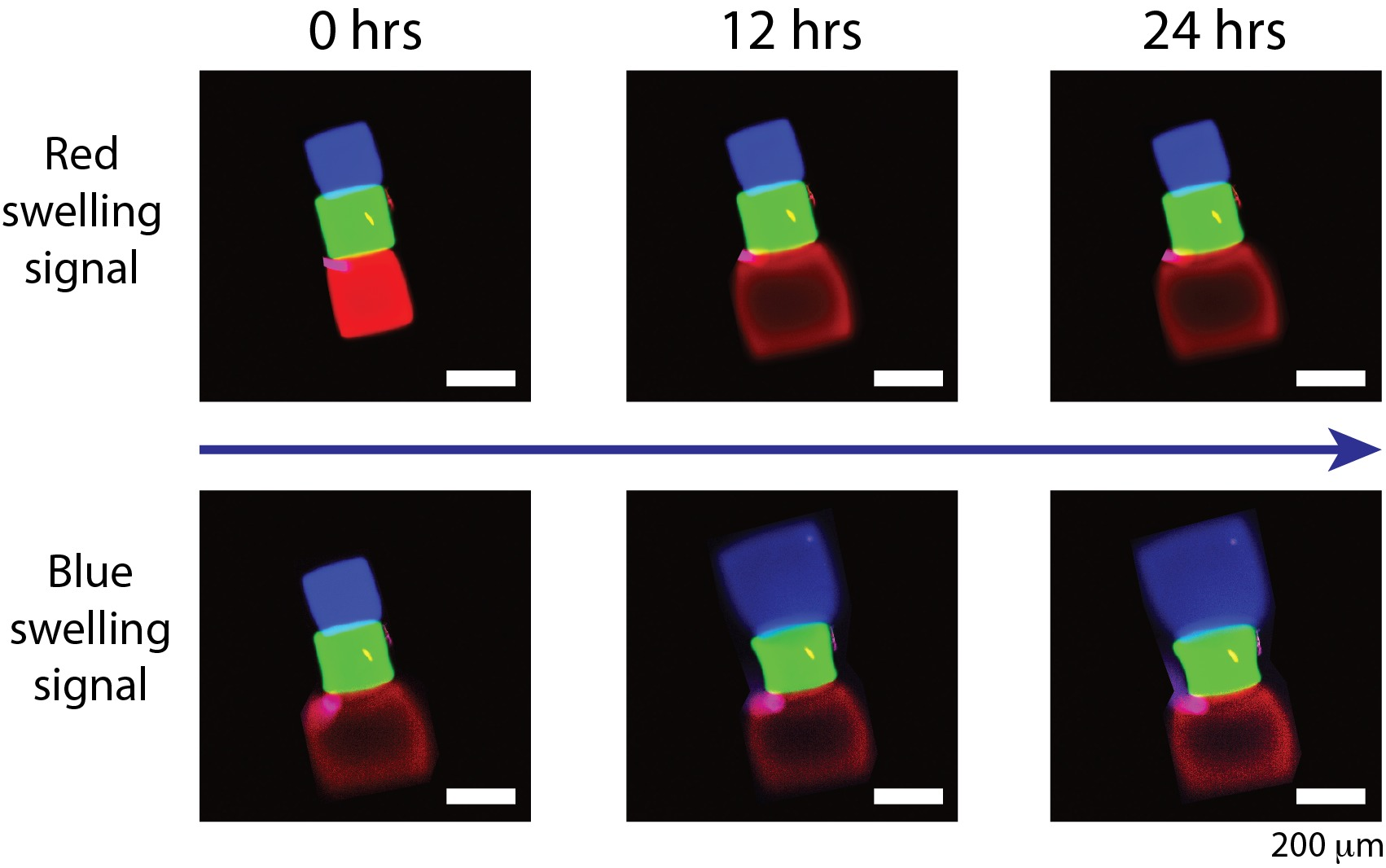

Supplement: S22 Fig — A) Original time-lapse micrograph of single-domain MAPDH hydrogel swelling. B) Automated micrograph analysis of single-domain MAPDH hydrogel swelling and measurement of length changes. C) Manual measurements of single-domain MAPDH hydrogel swelling and length change. (TIF) [file pone.0295923.s023.tif]

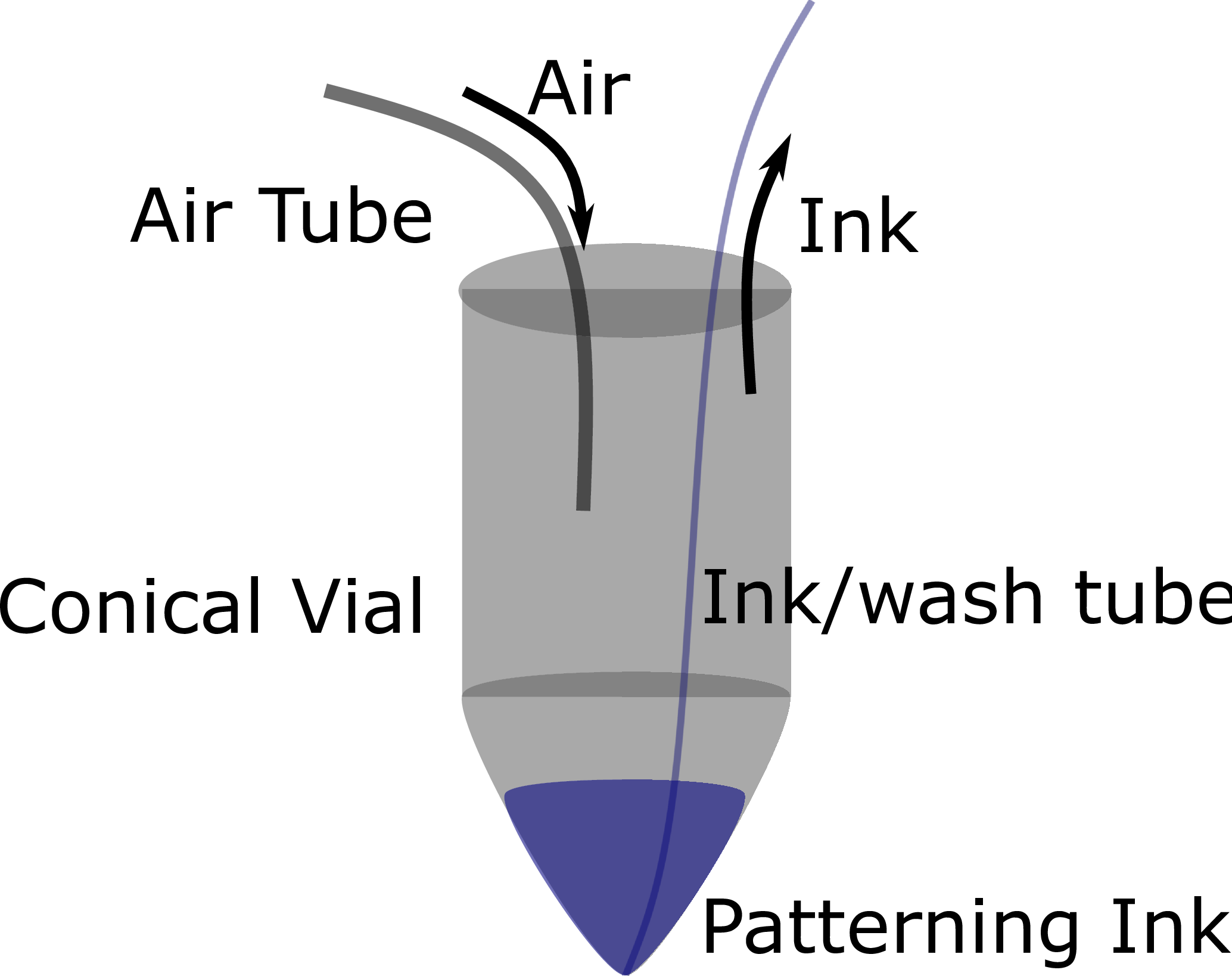

Supplement: S23 Fig — Micrographs of multi-domain hydrogel swelling after addition of red and blue swelling signals. (TIF) [file pone.0295923.s024.tif]
